# Distinct HLA Haplotypes Are Associated With an Altered Strength of SARS‐CoV‐2‐Specific T‐Cell Responses and Unfavorable Disease Courses

**DOI:** 10.1002/eji.202451497

**Published:** 2025-04-21

**Authors:** C. Dörnte, A. Datsi, V. Traska, J. Kostyra, M. Wagner, O. Brauns, C. Lamsfuß, H. Winkels, V. Balz, J. Enczmann, O. Adams, L. Mueller, H. Baurmann, B. Eiz‐Vesper, A. Bonifacius, R. V. Sorg, C. Dose, J. Schmitz, A. Richter, J. Fischer, M. Schuster

**Affiliations:** ^1^ Miltenyi Biotec B.V. & Co. KG Bergisch Gladbach Germany; ^2^ Clinic III for Internal Medicine University of Cologne Faculty of Medicine and University Hospital Cologne Cologne Germany; ^3^ Institute for Transplantation Diagnostics and Cell Therapeutics Medical Faculty and University Hospital Düsseldorf Heinrich Heine‐University Düsseldorf Düsseldorf Germany; ^4^ Center for Molecular Medicine Cologne (CMMC) University of Cologne Cologne Germany; ^5^ Institute for Virology Medical Faculty and University Hospital Düsseldorf Heinrich Heine‐University Düsseldorf Germany; ^6^ Institute of Transfusion Medicine and Transplant Engineering Medizinische Hochschule Hannover Hannover Germany

**Keywords:** APC, HLA haplotypes, NetMHCpan, SARS‐CoV‐2, T‐cell immunity

## Abstract

Infection with SARS‐CoV‐2 results in mild to severe COVID‐19 disease courses. Several studies showed the association of impaired T‐cell responses and certain HLA haplotypes with disease severity. However, it remained unclear if T‐cell activation was compromised due to a general reduction of presented epitopes or other intrinsic factors within APCs or T cells. Furthermore, a potential reduction of presented epitopes would suggest if an upcoming SARS‐CoV‐2 variant could escape T‐cell immunity. Hence, knowledge about the T‐cell epitope landscape of SARS‐CoV‐2 would allow to better understand mechanisms leading to severe disease and to estimate the potential stability of the T‐cell response in light of virus evolution, which might provide insights for future vaccine designs. Hence, in the present study, the T‐cell epitope landscape of SARS‐CoV‐2 was determined via *in vitro* T‐cell stimulation plus *in silico* prediction. HLAs associated with mild and severe disease courses showed almost the same potential in epitope presentation, suggesting intrinsic factors of APCs or T cells as contributors to the more severe disease courses. As T‐cell epitopes did also not originate from regions of SARS‐CoV‐2 having shown high mutation rates in the past, a relatively stable T‐cell response can be expected regarding new SARS‐CoV‐2 strains in the future. Analysis of the T‐cell epitope landscape of SARS‐CoV‐2 suggests T‐cell intrinsic factors as likely modulators of disease severity and that the capacity of MHC‐peptide presentation remains stable among circulating SARS‐CoV‐2 viral strains.

## Graphical abstract

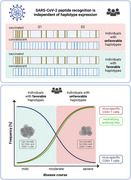

**Abbreviations**
APC: antigen‐presenting cells
CPE: cytopathic effect
HLA: human leukocyte antigen
MHC: major histocompatibility complex
TCR: T‐cell receptor

## 1 INTRODUCTION

There are two main approaches to predict T‐cell epitopes, and thereby strength and specificity. One approach is the identification and mapping of T‐cell epitopes, which are presented via major histocompatibility complex (MHC) class I and class II molecules. Such peptides have average lengths of 8 to 11 amino acids and 13 to 18 amino acids for MHC class I and MHC class II, respectively [1]. There are two main approaches to predict T‐cell epitopes. One is the *in silico* analysis of peptide binding to specific HLA molecules via software tools such as NetMHCpan. The other is the *in vitro* stimulation of T cells with peptides of interest and the correlation of an individual's HLA haplotype (i.e., the sum of all HLA alleles of that individual) with the T‐cell response. Feeding into both approaches, all potential peptides derived from an antigen from the N‐ to the C‐terminal part of the protein (referred to as sequential walk) provide information about the immunogenic regions of that specific antigen.

The underlying reasons for severe COVID‐19 disease courses are poorly understood. Accumulation of virus‐specific CD4^+^ T cells was reported in severe cases [2, 3, 4] and increased production of IFN‐γ by CD8^+^ T cells and a higher degree of T‐cell clonal expansion was described to be favorable for moderate COVID‐19 [5]. Additionally, the presence of bystander T‐cell activation in early anti‐viral defense was demonstrated in mild early‐onset disease [6], while elevated circulating plasmablasts and reduced germinal center responses were repeatedly observed in severe COVID‐19 [7]. To which extent the general strength of the T‐cell response might be dampened by the bystander T‐cell activation and its correlation with disease severity is still not fully understood. Since the underlying mechanisms leading to severe COVID‐19 infections are yet to be determined, the identification of individuals with a higher risk for severe disease is pending. Little work has been done to systematically associate peptide presentation with the severity of the disease and to compare the potential of HLA haplotypes from severe and mildly infected people with each other. Both analyses could support the identification of reliable clinical prediction markers.

In addition, novel virus strains can dampen the T‐cell responses gained via vaccination with first‐generation vaccines or natural infection with pre‐VOC viral strains. Certain strains are even able to completely escape it if they are presented in the context of certain HLA molecules [8, 9]. The extent to which this is supported by a dampened peptide presentation in different individuals or whether certain HLA molecules are predestined for this viral escape is still controversial. It is common understanding to correlate known T‐cell epitopes with areas undergoing high mutation rates in order to make assumptions about the degree to which T‐cell responses will be potentially diminished in the future.

In this study, T‐cell epitopes were identified and the T‐cell epitope landscape within various SARS‐CoV‐2 proteins was determined using groups of vaccinated and/or convalescent individuals. As these immunogenic protein regions did not overlap with areas, which show enhanced mutation rates, a relatively stable T‐cell response is expected toward upcoming virus strains. Furthermore, we could not find evidence that peptide presentation capacity from the immunogenic regions of HLA haplotypes from severe and mild diseases are strongly different. However, in line with previous studies [7], in long and more severe disease courses a bystander activation of unspecific CD8^+^ T cells, represented by waning frequencies of virus‐reactive CD8^+^ T cells, accompanied by significantly increased cell counts and a strong CD4^+^ T‐cell response were detected. Thus, we conclude that differences in disease courses (duration and severity) were likely not induced by the ability of certain HLA molecules to present specific peptides and to induce T‐cell responses, but rather the specificity of the T‐cell response directly.

Taken together, we suggest that the T‐cell response is likely to remain relatively stable in future SARS‐CoV‐2 virus strains. In addition, the main cause of severe disease courses is rather the result of intrinsic differences in T‐cell receptor (TCR)/HLA‐induced signaling and activation.

## 2 RESULTS

### 2.1 Composition of Cohort Study Groups and Epitope Analysis Strategy

**FIGURE 1:**
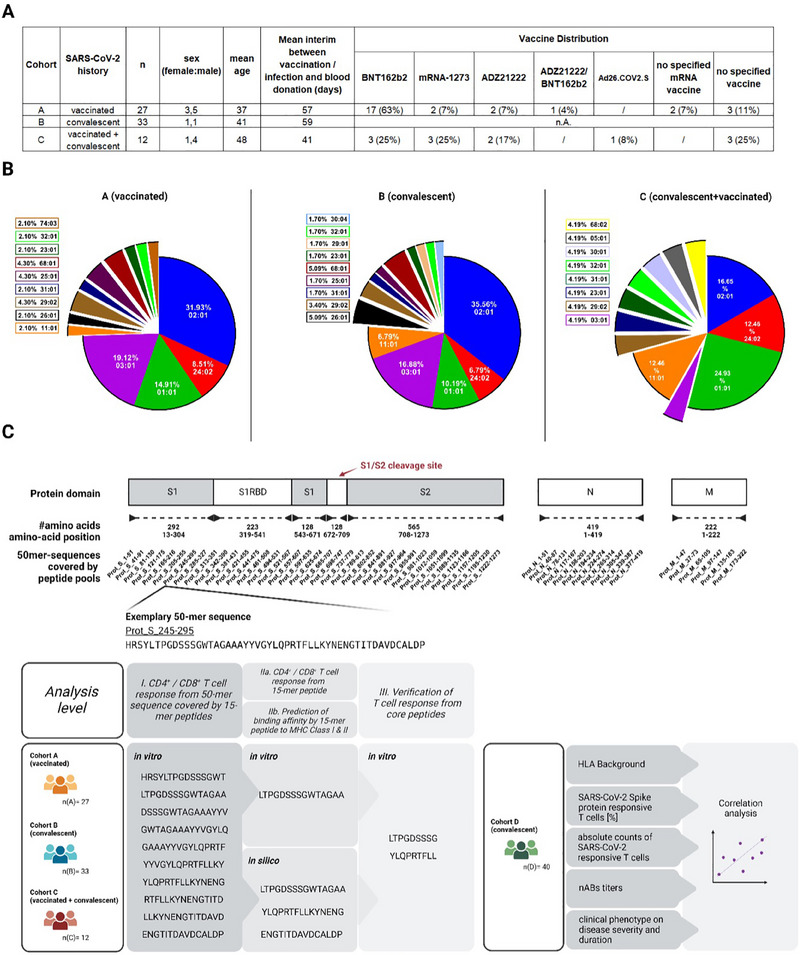
Experimental design and cohort description. (A) For cohorts A, B, and C, the cohort‐specific SARS‐CoV‐2 background, including the number of participants, the sex distribution ratio (female:male), the mean age of the participants, the interim days between vaccination or infection to the date of the blood donation as well as the administered vaccines are listed (B) Exemplary distribution of HLA‐A allotypes for cohorts A, B, and C. Shown is the frequency of each allele amongst the total number of HLA‐A allotypes expressed within the cohort. (C) Given the protein sequence of the SARS‐CoV‐2 spike, nucleocapsid, and membrane proteins, 53 peptide pools covering approximately 50 amino acid‐long, overlapping sequences were designed (top). These peptide pools include 10 consecutive 15‐mer peptides, with an 11 amino acid overlap to each other. Vaccinated (cohort A), convalescent (cohort B) as well as vaccinated and convalescent individuals (cohort C) were recruited to assess (I) the CD4^+^ and CD8^+^ T‐cell response against these aforementioned 50‐mer sequences, and (IIa) the CD4^+^ and CD8^+^ T‐cell response against 15‐mer peptides that are derived from immunogenic peptide pools by conducting *in vitro* stimulation approaches. Furthermore, (IIb) the binding affinity of peptides to MHC Class I and II molecules were *in silico* predicted using NetMHCpan with regard to the HLA background of the individual study subjects. Lastly, (III) T‐cell responses from core peptides were validated *in vitro*. Study subjects of cohort D, comprising independent convalescent individuals, were HLA phenotyped. The frequency as well as absolute number of SARS‐CoV‐2 responsive T cells, neutralizing antibody (nAB) titers, and their clinical phenotype were assessed. All parameters were included in correlation analyses.

To determine the T‐cell epitope landscape, three cohort groups were analyzed: (A) vaccinated (*n* = 27), (B) convalescent (*n* = 33), and (C) convalescent vaccinated (*n* = 12) study subjects (Figure 1). For all study subjects background information is provided (Figure 1A,B; Figure S1, Table S1).

We established an experimental workflow comprised of three analysis levels (Figure 1C, bottom left graphic): On the first level, T cells were stimulated *in vitro*. For this purpose, 53 peptide pools were used, and all pools together covered the entire S, N, and M antigens from their N‐ to the C‐termini. They comprised consecutive peptides, mostly 15‐mers which had 11 amino acids overlap with each other. Each single peptide pool covered a 50 amino acid‐long region of the indicated SARS‐CoV‐2 antigens (Figure 1C, “I”). This first analysis level provided information on whether and which 50‐amino acids long region were immunogenic and, thus, could mount a T‐cell response (Table 1). On the second analysis level, the available 15‐mer peptides for the previously identified immunogenic 50‐amino‐acid‐long regions were used for T‐cell stimulation to further narrow down a potential T‐cell epitope. In addition on the second level, an *in silico*, prediction of potential MHC‐binders was conducted using the software NetMHC(II)pan and correlated with *in vitro* findings (Figure 1C, “IIa/IIb”). Therewith, we determined theoretical binding affinities of 15mer single peptides to the HLA alleles of interest. Simultaneously the accompanying 9‐mer MHC binding core peptide sequences were defined.

**TABLE 1:** Number of reactive donors among activated T cells against 50 amino‐acid long regions of SARS‐CoV‐2 structural proteins.

Protein	Peptide Pool	Sequence covered by 15‐mer peptide pools	Number of reactive donors among CD8+TNFɑ+IFNɣ+ T cells (frequency of total)			Number of reactive donors among CD4+CD154+TNFɑ+ T cells (frequency of total)		
			Cohort A	Cohort B	Cohort C	Cohort A	Cohort B	Cohort C
Spike	Prot_S_1‐51	MFVFLVLLPLVSSQCVNLTTRTQLPPAYTNSFTRGVYYPDKVFRSSVLHST	8 (72.7)	3 (27.3)	0	3 (30)	7 (70)	0
	Prot_S_41‐91	KVFRSSVLHSTQDLFLPFFSNVTWFHAIHVSGTNGTKRFDNPVLPFNDGVY	4 (44.4)	5 (55.6)	0	1 (20)	4 (80)	0
	Prot_S_81‐130	NPVLPFNDGVYFASTEKSNIIRGWIFGTTLDSKTQSLLIVNNATNVVIKV	5 (55.6)	4 (44.4)	0	4 (50)	4 (50)	0
	Prot_S_121‐175	NNATNVVIKVCEFQFCNDPFLGVYYHKNNKSWMESEFRVYSSANNCTFEYVSQPF	11 (64.7)	5 (29.4)	1 (5.9)	2 (15.4)	10 (76.9)	1 (7.7)
	Prot_S_165‐216	NCTFEYVSQPFLMDLEGKQGNFKNLREFVFKNIDGYFKIYSKHTPINLVRDL	1 (25)	3 (75)	0	4 (50)	4 (50)	0
	Prot_S_205‐255	SKHTPINLVRDLPQGFSALEPLVDLPIGINITRFQTLLALHRSYLTPGDSS	1 (50)	1 (50)	0	2 (66.7)	1 (33.3)	0
	Prot_S_245‐295	HRSYLTPGDSSSGWTAGAAAYYVGYLQPRTFLLKYNENGTITDAVDCALDP	4 (66.7)	2 (33.3)	0	3 (75)	1 (25)	0
	Prot_S_285‐327	ITDAVDCALDPLSETKCTLKSFTVEKGIYQTSNFRVQPTESIV	0	0	1 (100)	1 (100)	0	0
	Prot_S_313‐351	YQTSNFRVQPTESIVRFPNITNLCPFGEVFNATRFASVY	0	1 (100)	0	0	1 (100)	0
	Prot_S_342‐390	FNATRFASVYAWNRKRISNCVADYSVLYNSASFSTFKCYGVSPTKLNDL	5 (62.5)	1 (12.5)	2 (25)	3 (20)	10 (66.7)	2 (13.3)
	Prot_S_381‐431	GVSPTKLNDLCFTNVYADSFVIRGDEVRQIAPGQTGKIADYNYKLPDDFTG	1 (33.3)	2 (66.7)	0	0	1 (100)	0
	Prot_S_421‐455	YNYKLPDDFTGCVIAWNSNNLDSKVGGNYNYLYRL	0	1 (100)	0	1 (50)	1 (50)	0
	Prot_S_441‐475	LDSKVGGNYNYLYRLFRKSNLKPFERDISTEIYQA	0	3 (75)	1 (25)	1 (20)	3 (60)	1 (20)
	Prot_S_461‐508	LKPFERDISTEIYQAGSTPCNGVEGFNCYFPLQSYGFQPTNGVGYQPY	0	4 (100)	0	0	1 (100)	0
	Prot_S_494‐531	SYGFQPTNGVGYQPYRVVVLSFELLHAPATVCGPKKST	1 (33.3)	2 (66.7)	0	0	1 (100)	0
	Prot_S_521‐567	PATVCGPKKSTNLVKNKCVNFNFNGLTGTGVLTESNKKFLPFQQFGR	1 (33.3)	2 (66.7)	0	3 (50)	3 (50)	0
	Prot_S_557‐607	KKFLPFQQFGRDIADTTDAVRDPQTLEILDITPCSFGGVSVITPGTNTSNQ	0	1 (100)	0	0	1 (100)	0
	Prot_S_597‐635	VITPGTNTSNQVAVLYQDVNCTEVPVAIHADQLTPTWRV	0	1 (100)	0	1 (100)	0	0
	Prot_S_625‐674	HADQLTPTWRVYSTGSNVFQTRAGCLIGAEHVNNSYECDIPIGAGICASY	1 (33.3)	2 (66.7)	0	2 (100)	0	0
	Prot_S_665‐707	PIGAGICASYQTQTNSPRRARSVASQSIIAYTMSLGAENSVAY	2 (33.3)	3 (50)	1 (16.7)	2 (66.7)	1 (33.3)	0
	Prot_S_698‐747	SLGAENSVAYSNNSIAIPTNFTISVTTEILPVSMTKTSVDCTMYICGDST	1 (16.7)	5 (83.3)	0	3 (100)	0	0
	Prot_S_737‐779	DCTMYICGDSTECSNLLLQYGSFCTQLNRALTGIAVEQDKNTQ	1 (33.3)	2 (66.7)	0	4 (66.7)	2 (33.3)	0
	Prot_S_769‐813	GIAVEQDKNTQEVFAQVKQIYKTPPIKDFGGFNFSQILPDPSKPS	1 (16.7)	4 (66.7)	1 (16.7)	3 (37.5)	3 (37.5)	2 (25)
	Prot_S_802‐852	FSQILPDPSKPSKRSFIEDLLFNKVTLADAGFIKQYGDCLGDIAARDLICA	2 (40)	3 (60)	0	3 (18.8)	10 (62.5)	3 (18.8)
	Prot_S_841‐891	LGDIAARDLICAQKFNGLTVLPPLLTDEMIAQYTSALLAGTITSGWTFGAG	1 (20)	4 (80)	0	4 (66.7)	1 (16.7)	1 (16.7)
	Prot_S_881‐927	TITSGWTFGAGAALQIPFAMQMAYRFNGIGVTQNVLYENQKLIANQF	4 (66.7)	2 (33.3)	0	0	6 (85.7)	1 (14.3)
	Prot_S_917‐964	YENQKLIANQFNSAIGKIQDSLSSTASALGKLQDVVNQNAQALNTLVK	0	2 (66.7)	1 (33.3)	1 (33.3)	1 (33.3)	1 (33.3)
	Prot_S_955‐991	NAQALNTLVKQLSSNFGAISSVLNDILSRLDKVEAEV	2 (40)	3 (60)	0	1 (50)	1 (50)	0
	Prot_S_981‐1023	LSRLDKVEAEVQIDRLITGRLQSLQTYVTQQLIRAAEIRASAN	3 (50)	3 (50)	0	3 (50)	3 (50)	0
	Prot_S_1012‐1059	LIRAAEIRASANLAATKMSECVLGQSKRVDFCGKGYHLMSFPQSAPHG	0	3 (100)	0	1 (33.3)	2 (66.7)	0
	Prot_S_1051‐1099	SFPQSAPHGVVFLHVTYVPAQEKNFTTAPAICHDGKAHFPREGVFVSNG	1 (20)	4 (80)	0	4 (66.7)	2 (33.3)	0
	Prot_S_1089‐1135	FPREGVFVSNGTHWFVTQRNFYEPQIITTDNTFVSGNCDVVIGIVNN	0	4 (100)	0	3 (100)	0	0
	Prot_S_1123‐1166	SGNCDVVIGIVNNTVYDPLQPELDSFKEELDKYFKNHTSPDVDL	0	3 (75)	1 (25)	0	1 (100)	0
	Prot_S_1157‐1205	KNHTSPDVDLGDISGINASVVNIQKEIDRLNEVAKNLNESLIDLQELGK	0	2 (100)	0	2 (100)	0	0
	Prot_S_1195‐1230	ESLIDLQELGKYEQYIKWPWYIWLGFIAGLIAIVMV	2 (40)	2 (40)	1 (20)	3 (75)	1 (25)	0
	Prot_S_1222‐1273	AGLIAIVMVTIMLCCMTSCCSCLKGCCSCGSCCKFDEDDSEPVLKGVKLHYT	3 (60)	2 (40)	0	1 (33.3)	2 (66.7)	0
Nucleocapsid	Prot_N_1‐51	MSDNGPQNQRNAPRITFGGPSDSTGSNQNGERSGARSKQRRPQGLPNNTAS	0	2 (100)	0	2 (100)	0	0
	Prot_N_40‐87	RRPQGLPNNTASWFTALTQHGKEDLKFPRGQGVPINTNSSPDDQIGYY	0	1 (100)	0	0	0	1 (100)
	Prot_N_78‐131	SSPDDQIGYYRRATRRIRGGDGKMKDLSPRWYFYYLGTGPEAGLPYGANK	0	3 (75)	1 (25)	1 (100)	0	0
	Prot_N_117‐167	PEAGLPYGANKDGIIWVATEGALNTPKDHIGTRNPANNAAIVLQLPQGTTL	0	0	0	2 (100)	0	0
	Prot_N_158‐203	VLQLPQGTTLPKGFYAEGSRGGSQASSRSSSRSRNSSRNSTPGSSR	0	1 (100)	0	0	0	0
	Prot_N_194‐234	SRNSTPGSSRGTSPARMAGNGGDAALALLLLDRLNQLESKM	0	2 (100)	0	4 (100)	0	0
	Prot_N_224‐274	LDRLNQLESKMSGKGQQQQGQTVTKKSAAEASKKPRQKRTATKAYNVTQAF	0	4 (100)	0	2 (100)	0	0
	Prot_N_265‐314	TKAYNVTQAFGRRGPEQTQGNFGDQELIRQGTDYKHWPQIAQFAPSASAF	0	4 (100)	0	2 (100)	0	0
	Prot_N_305‐347	AQFAPSASAFFGMSRIGMEVTPSGTWLTYTGAIKLDDKDPNFK	0	1 (50)	1 (50)	0	0	0
	Prot_N_339‐387	LDDKDPNFKDQVILLNKHIDAYKTFPPTEPKKDKKKKADETQALPQRQK	0	0	1 (100)	0	0	2 (100)
	Prot_N_377‐419	DETQALPQRQKKQQTVTLLPAADLDDFSKQLQQSMSSADSTQA	0	1 (100)	0	1 (100)	0	0
Membrane	Prot_M_1‐47	MADSNGTITVEELKKLLEQWNLVIGFLFLTWICLLQFAYANRNRFLY	0	5 (100)	0	4 (100)	0	0
	Prot_M_37‐73	FAYANRNRFLYIIKLIFLWLLWPVTLACFVLAAVYRI	0	2 (100)	0	3 (100)	0	0
	Prot_M_65‐105	FVLAAVYRINWITGGIAIAMACLVGLMWLSYFIASFRLFAR	0	1 (50)	1 (50)	1 (100)	0	0
	Prot_M_97‐147	IASFRLFARTRSMWSFNPETNILLNVPLHGTILTRPLLESELVIGAVILRG	0	4 (100)	0	4 (100)	0	0
	Prot_M_135‐183	ESELVIGAVILRGHLRIAGHHLGRCDIKDLPKEITVATSRTLSYYKLGA	0	6 (100)	0	7 (100)	0	0
	Prot_M_173‐222	SRTLSYYKLGASQRVAGDSGFAAYSRYRIGNYKLNTDHSSSSDNIALLVQ	0	2 (66.7)	1 (33.3)	9 (81.8)	0	2 (18.2)

*Note*: Rows refer to an individual 50 amino acid‐long region derived from the spike, nucleocapsid, or membrane protein (1st column). Each region is covered by ten consecutive 15‐mer single peptides. The individual name of the peptide pool (2nd column), as well as the specific sequence (3rd column), are indicated, followed by the number (and frequency of total) of reactive donors from cohorts A, B, and C among CD8^+^TNFɑ^+^IFNɣ^+^—And CD4^+^CD154^+^TNFɑ+ T cells.

The latter is verified on the third and last analysis level. Here, 9‐mer immunogenic core peptides, within 15‐mer verified and predicted immunogenic peptides (Tables 2 and 3), were *in vitro* validated for their potential to induce T‐cell responses, and NetMHC(II)pan‐predicted MHC restrictions were confirmed or refuted (Figure 1C, “III”; Table 4). An exemplary gating strategy or the flow cytometric identification of T‐cell reactivity upon stimulation with a 9‐mer core peptide (HLA‐A01:01_P2) and SARS‐CoV‐2 peptide pools (SARS‐CoV‐2 PepTivator S/M/N) is provided in Figure S2.

**TABLE 2:** T cell response‐inducing 15‐mer peptides identified from *in vitro* stimulation assays.

15‐mer peptide	Ancestor peptide pool	Peptide sequence	# Reactive donors	Predicted HLA restriction						
CoV_Prot_S_1‐14	CoV_Prot_S_1‐51	MFVFLVLLPLVSSQ	3	HLA‐C03:04	HLA‐C02:02					
CoV_Prot_S_1‐14	CoV_Prot_S_1‐51	MFVFLVLLPLVSSQ	1	DRB1_1501						
CoV_Prot_S_5‐19	CoV_Prot_S_1‐51	LVLLPLVSSQCVNLT	2	HLA‐B51:01	HLA‐C03:04					
CoV_Prot_S_10‐24	CoV_Prot_S_1‐51	LVSSQCVNLTTRTQL	1	HLA‐C07:04						
CoV_Prot_S_13‐27	CoV_Prot_S_1‐51	SQCVNLTTRTQLPPA	1	HLA‐B37:01						
CoV_Prot_S_13‐27	CoV_Prot_S_1‐51	SQCVNLTTRTQLPPA	1	DRB1_1301						
CoV_Prot_S_18‐32	CoV_Prot_S_1‐51	LTTRTQLPPAYTNSF	1	DRB1_0101						
CoV_Prot_S_24‐38	CoV_Prot_S_1‐51	LPPAYTNSFTRGVYY	2	HLA‐C15:02						
CoV_Prot_S_29‐43	CoV_Prot_S_1‐51	TNSFTRGVYYPDKVF	2	HLA‐C02:02	HLA‐C07:01					
CoV_Prot_S_29‐43	CoV_Prot_S_1‐51	TNSFTRGVYYPDKVF	1	DRB1_0401						
CoV_Prot_S_33‐47	CoV_Prot_S_1‐51	TRGVYYPDKVFRSSV	1	HLA‐B15:01						
CoV_Prot_S_33‐47	CoV_Prot_S_1‐51	TRGVYYPDKVFRSSV	2	DRB1_0401						
CoV_Prot_S_37‐51	CoV_Prot_S_1‐51	YYPDKVFRSSVLHST	1	DRB1_0401						
CoV_Prot_S_41‐55	CoV_Prot_S_1‐51	KVFRSSVLHSTQDLF	1	HLA‐B14:01						
CoV_Prot_S_44‐58	CoV_Prot_S_1‐51	RSSVLHSTQDLFLPF	2	HLA‐A26:01						
CoV_Prot_S_44‐58	CoV_Prot_S_41‐91	RSSVLHSTQDLFLPF	1	DRB1_0101						
CoV_Prot_S_49‐63	CoV_Prot_S_41‐91	HSTQDLFLPFFSNVT	1	HLA‐A26:01						
CoV_Prot_S_57‐71	CoV_Prot_S_41‐91	PFFSNVTWFHAIHVS	1	HLA‐C15:02						
CoV_Prot_S_57‐71	CoV_Prot_S_41‐91	PFFSNVTWFHAIHVS	1	DRB1_0101						
CoV_Prot_S_62‐76	CoV_Prot_S_41‐91	VTWFHAIHVSGTNGT	2	HLA‐C15:02						
CoV_Prot_S_62‐76	CoV_Prot_S_41‐91	VTWFHAIHVSGTNGT	1	DRB1_0101						
CoV_Prot_S_72‐86	CoV_Prot_S_41‐91	GTNGTKRFDNPVLPF	1	HLA‐B27:05						
CoV_Prot_S_72‐86	CoV_Prot_S_41‐91	GTNGTKRFDNPVLPF	1	DRB1_1501						
CoV_Prot_S_77‐91	CoV_Prot_S_41‐91	KRFDNPVLPFNDGVY	1	HLA‐B27:05						
CoV_Prot_S_81‐95	CoV_Prot_S_81‐130	NPVLPFNDGVYFAST	1	HLA‐B35:01						
CoV_Prot_S_117‐130	CoV_Prot_S_81‐130	LLIVNNATNVVIKV	1	HLA‐B51:01						
CoV_Prot_S_117‐130	CoV_Prot_S_81‐130	LLIVNNATNVVIKV	1	DRB1_0401						
CoV_Prot_S_121‐135	CoV_Prot_S_121‐175	NNATNVVIKVCEFQF	1	HLA‐B51:01						
CoV_Prot_S_129‐143	CoV_Prot_S_121‐175	KVCEFQFCNDPFLGV	1	HLA‐C07:02						
CoV_Prot_S_129‐143	CoV_Prot_S_121‐175	KVCEFQFCNDPFLGV	1	DRB1_1101						
CoV_Prot_S_133‐147	CoV_Prot_S_121‐175	FQFCNDPFLGVYYHK	3	DRB1_1501	DRB1_1501					
CoV_Prot_S_138‐152	CoV_Prot_S_121‐175	DPFLGVYYHKNNKSW	1	DRB1_1501						
CoV_Prot_S_150‐164	CoV_Prot_S_121‐175	KSWMESEFRVYSSAN	2	HLA‐A29:02	HLA‐A01:01					
CoV_Prot_S_150‐164	CoV_Prot_S_121‐175	KSWMESEFRVYSSAN	1	DRB1_1101						
CoV_Prot_S_154‐168	CoV_Prot_S_121‐175	ESEFRVYSSANNCTF	1	HLA‐C16:01						
CoV_Prot_S_157‐171	CoV_Prot_S_121‐175	FRVYSSANNCTFEYV	1	HLA‐B35:01						
CoV_Prot_S_157‐171	CoV_Prot_S_121‐175	FRVYSSANNCTFEYV	1	DRB1_1501						
CoV_Prot_S_161‐175	CoV_Prot_S_121‐175	SSANNCTFEYVSQPF	1	HLA‐C04:01						
CoV_Prot_S_165‐179	CoV_Prot_S_165‐216	NCTFEYVSQPFLMDL	3	HLA‐B40:01	HLA‐C04:01	HLA‐B40:02				
CoV_Prot_S_165‐179	CoV_Prot_S_165‐216	NCTFEYVSQPFLMDL	2	DRB1_1601	DRB1_1501					
CoV_Prot_S_170‐184	CoV_Prot_S_165‐216	YVSQPFLMDLEGKQG	1	HLA‐C02:02						
CoV_Prot_S_178‐192	CoV_Prot_S_165‐216	DLEGKQGNFKNLREF	1	HLA‐B15:01						
CoV_Prot_S_178‐192	CoV_Prot_S_165‐216	DLEGKQGNFKNLREF	1	DQA10102‐DQB10502						
CoV_Prot_S_189‐203	CoV_Prot_S_165‐216	LREFVFKNIDGYFKI	2	DRB1_1501						
CoV_Prot_S_194‐208	CoV_Prot_S_165‐216	FKNIDGYFKIYSKHT	2	DRB1_1501						
CoV_Prot_S_198‐212	CoV_Prot_S_165‐216	DGYFKIYSKHTPINL	2	DRB1_1501						
CoV_Prot_S_202‐216	CoV_Prot_S_165‐216	KIYSKHTPINLVRDL	2	DRB1_1501						
CoV_Prot_S_229‐243	CoV_Prot_S_205‐255	LPIGINITRFQTLLA	1	HLA‐B08:01						
CoV_Prot_S_233‐247	CoV_Prot_S_205‐255	INITRFQTLLALHRS	1	DQA10501‐DQB10501						
CoV_Prot_S_236‐250	CoV_Prot_S_205‐255	TRFQTLLALHRSYLT	1	DRB1_0101						
CoV_Prot_S_241‐255	CoV_Prot_S_205‐255	LLALHRSYLTPGDSS	1	DRB1_1501						
CoV_Prot_S_245‐259	CoV_Prot_S_245‐295	HRSYLTPGDSSSGWT	1	DQA10501‐DQB10301						
CoV_Prot_S_249‐263	CoV_Prot_S_245‐295	LTPGDSSSGWTAGAA	1	HLA‐A01:01						
CoV_Prot_S_253‐267	CoV_Prot_S_245‐295	DSSSGWTAGAAAYYV	1	HLA‐A01:01						
CoV_Prot_S_257‐271	CoV_Prot_S_245‐295	GWTAGAAAYYVGYLQ	1	HLA‐A01:01						
CoV_Prot_S_261‐275	CoV_Prot_S_245‐295	GAAAYYVGYLQPRTF	1	HLA‐C03:03						
CoV_Prot_S_265‐279	CoV_Prot_S_245‐295	YYVGYLQPRTFLLKY	3	HLA‐A02:01						
CoV_Prot_S_265‐279	CoV_Prot_S_245‐295	YYVGYLQPRTFLLKY	1	DRB1_1101						
CoV_Prot_S_269‐283	CoV_Prot_S_245‐295	YLQPRTFLLKYNENG	3	HLA‐A02:01						
CoV_Prot_S_269‐283	CoV_Prot_S_245‐295	YLQPRTFLLKYNENG	1	DRB1_1101						
CoV_Prot_S_285‐299	CoV_Prot_S_285‐327	ITDAVDCALDPLSET	1	HLA‐C05:01						
CoV_Prot_S_289‐303	CoV_Prot_S_285‐327	VDCALDPLSETKCTL	1	HLA‐C05:01						
CoV_Prot_S_304‐319	CoV_Prot_S_285‐327	KSFTVEKGIYQTSNFR	1	HLA‐C02:02						
CoV_Prot_S_342‐356	CoV_Prot_S_342‐390	FNATRFASVYAWNRK	3	DQA10501‐DQB10301	DRB1_1501					
CoV_Prot_S_345‐359	CoV_Prot_S_342‐390	TRFASVYAWNRKRIS	2	HLA‐A03:01	HLA‐B27:05					
CoV_Prot_S_345‐359	CoV_Prot_S_342‐390	TRFASVYAWNRKRIS	7	DRB1_1103	DRB1_1302	DRB1_1101	DRB1_1301	DRB1_1501	DRB1_1501	DRB1_1302
CoV_Prot_S_349‐363	CoV_Prot_S_342‐390	SVYAWNRKRISNCVA	5	DRB1_1101	DRB1_1301	DRB1_0401	DRB1_1302			
CoV_Prot_S_353‐367	CoV_Prot_S_342‐390	WNRKRISNCVADYSV	4	DRB1_0401	DRB1_1501	DRB1_1302				
CoV_Prot_S_369‐383	CoV_Prot_S_342‐390	YNSASFSTFKCYGVS	1	HLA‐C02:02						
CoV_Prot_S_369‐383	CoV_Prot_S_342‐390	YNSASFSTFKCYGVS	3	DRB1_1501	DRB1_0701					
CoV_Prot_S_373‐387	CoV_Prot_S_342‐390	SFSTFKCYGVSPTKL	5	HLA‐A24:02	HLA‐A03:01					
CoV_Prot_S_373‐387	CoV_Prot_S_342‐390	SFSTFKCYGVSPTKL	2	DRB1_1501						
CoV_Prot_S_376‐390	CoV_Prot_S_342‐390	TFKCYGVSPTKLNDL	4	HLA‐A24:02	HLA‐A03:01					
CoV_Prot_S_376‐390	CoV_Prot_S_342‐390	TFKCYGVSPTKLNDL	2	DRB1_1501						
CoV_Prot_S_385‐399	CoV_Prot_S_381‐431	TKLNDLCFTNVYADS	1	DRB1_0701						
CoV_Prot_S_445‐459	CoV_Prot_S_441‐475	VGGNYNYLYRLFRKS	1	HLA‐A23:01						
CoV_Prot_S_445‐459	CoV_Prot_S_441‐475	VGGNYNYLYRLFRKS	1	DRB1_0301						
CoV_Prot_S_457‐471	CoV_Prot_S_441‐475	RKSNLKPFERDISTE	1	DRB1_0301						
CoV_Prot_S_457‐471	CoV_Prot_S_441‐475	RKSNLKPFERDISTE	1	DRB1_0301						
CoV_Prot_S_461‐475	CoV_Prot_S_441‐475	LKPFERDISTEIYQA	1	DRB1_0401						
CoV_Prot_S_509‐523	CoV_Prot_S_494‐531	RVVVLSFELLHAPAT	1	DRB1_0101						
CoV_Prot_S_553‐567	CoV_Prot_S_521‐567	TESNKKFLPFQQFGR	1	HLA‐C07:01						
CoV_Prot_S_553‐567	CoV_Prot_S_521‐567	TESNKKFLPFQQFGR	1	DRB1_1302						
CoV_Prot_S_669‐683	CoV_Prot_S_665‐707	GICASYQTQTNSPRR	2	HLA‐A11:01						
CoV_Prot_S_685‐699	CoV_Prot_S_665‐707	RSVASQSIIAYTMSL	1	HLA‐A02:01						
CoV_Prot_S_698‐712	CoV_Prot_S_698‐747	SLGAENSVAYSNNSI	1	HLA‐C14:02						
CoV_Prot_S_702‐716	CoV_Prot_S_698‐747	ENSVAYSNNSIAIPT	1	HLA‐B55:01						
CoV_Prot_S_713‐727	CoV_Prot_S_698‐747	AIPTNFTISVTTEIL	1	HLA‐B51:01						
CoV_Prot_S_719‐733	CoV_Prot_S_698‐747	TISVTTEILPVSMTK	1	HLA‐A11:01						
CoV_Prot_S_722‐736	CoV_Prot_S_698‐747	VTTEILPVSMTKTSV	1	HLA‐A11:01						
CoV_Prot_S_725‐739	CoV_Prot_S_698‐747	EILPVSMTKTSVDCT	1	HLA‐A11:01						
CoV_Prot_S_753‐765	CoV_Prot_S_737‐779	LLQYGSFCTQLNR	1	DRB1_1501						
CoV_Prot_S_765‐779	CoV_Prot_S_737‐779	RALTGIAVEQDKNTQ	1	DRB1_1301						
CoV_Prot_S_777‐791	CoV_Prot_S_769‐813	NTQEVFAQVKQIYKT	2	DRB1_1301	DRB1_1501					
CoV_Prot_S_781‐795	CoV_Prot_S_769‐813	VFAQVKQIYKTPPIK	1	DRB1_1501						
CoV_Prot_S_788‐802	CoV_Prot_S_769‐813	IYKTPPIKDFGGFNF	1	DRB1_1301						
CoV_Prot_S_799‐813	CoV_Prot_S_769‐813	GFNFSQILPDPSKPS	1	HLA‐B15:01						
CoV_Prot_S_799‐813	CoV_Prot_S_769‐813	GFNFSQILPDPSKPS	1	DRB1_1301						
CoV_Prot_S_802‐816	CoV_Prot_S_802‐852	FSQILPDPSKPSKRS	8	DRB1_1501	DRB1_0401	DRB1_1501	DRB1_0301			
CoV_Prot_S_809‐823	CoV_Prot_S_802‐852	PSKPSKRSFIEDLLF	2	DRB1_1501						
CoV_Prot_S_813‐827	CoV_Prot_S_802‐852	SKRSFIEDLLFNKVT	1	HLA‐C07:01						
CoV_Prot_S_813‐827	CoV_Prot_S_802‐852	SKRSFIEDLLFNKVT	8	DQA10102‐DQB10501	DRB1_0401	DRB1_0101	DRB1_0301			
CoV_Prot_S_817‐831	CoV_Prot_S_802‐852	FIEDLLFNKVTLADA	2	DRB1_0801	DRB1_0401					
CoV_Prot_S_821‐835	CoV_Prot_S_802‐852	LLFNKVTLADAGFIK	1	HLA‐B08:01						
CoV_Prot_S_821‐835	CoV_Prot_S_802‐852	LLFNKVTLADAGFIK	2	DRB1_0101	DRB1_0301					
CoV_Prot_S_825‐839	CoV_Prot_S_802‐852	KVTLADAGFIKQYGD	1	HLA‐B18:01						
CoV_Prot_S_825‐839	CoV_Prot_S_802‐852	KVTLADAGFIKQYGD	1	DRB1_0801						
CoV_Prot_S_829‐843	CoV_Prot_S_802‐852	ADAGFIKQYGDCLGD	1	DRB1_0101						
CoV_Prot_S_838‐852	CoV_Prot_S_802‐852	GDCLGDIAARDLICA	1	HLA‐C12:03						
CoV_Prot_S_838‐852	CoV_Prot_S_802‐852	GDCLGDIAARDLICA	1	DRB1_0301						
CoV_Prot_S_844‐858	CoV_Prot_S_841‐891	IAARDLICAQKFNGL	1	HLA‐C08:02						
CoV_Prot_S_865‐879	CoV_Prot_S_841‐891	LTDEMIAQYTSALLA	2	HLA‐C08:02	HLA‐A01:01					
CoV_Prot_S_865‐879	CoV_Prot_S_841‐891	LTDEMIAQYTSALLA	1	DRB1_1501						
CoV_Prot_S_869‐883	CoV_Prot_S_841‐891	MIAQYTSALLAGTIT	1	DRB1_1501						
CoV_Prot_S_873‐887	CoV_Prot_S_841‐891	YTSALLAGTITSGWT	1	DRB1_1302						
CoV_Prot_S_885‐899	CoV_Prot_S_881‐927	GWTFGAGAALQIPFA	1	HLA‐C03:03						
CoV_Prot_S_885‐899	CoV_Prot_S_881‐927	GWTFGAGAALQIPFA	1	DRB1_0401						
CoV_Prot_S_889‐902	CoV_Prot_S_881‐927	GAGAALQIPFAMQM	1	HLA‐B15:01						
CoV_Prot_S_892‐906	CoV_Prot_S_881‐927	AALQIPFAMQMAYRF	3	HLA‐B56:01	HLA‐B15:01	HLA‐C03:03				
CoV_Prot_S_892‐906	CoV_Prot_S_881‐927	AALQIPFAMQMAYRF	1	DRB1_0101						
CoV_Prot_S_896‐910	CoV_Prot_S_881‐927	IPFAMQMAYRFNGIG	2	HLA‐B56:01	HLA‐C03:03					
CoV_Prot_S_896‐910	CoV_Prot_S_881‐927	IPFAMQMAYRFNGIG	1	DRB1_0101						
CoV_Prot_S_917‐931	CoV_Prot_S_917‐964	YENQKLIANQFNSAI	1	DRB1_1302						
CoV_Prot_S_921‐935	CoV_Prot_S_917‐964	KLIANQFNSAIGKIQ	1	DRB1_1302						
CoV_Prot_S_977‐991	CoV_Prot_S_955‐991	LNDILSRLDKVEAEV	1	HLA‐B51:01						
CoV_Prot_S_986‐1000	CoV_Prot_S_981‐1023	KVEAEVQIDRLITGR	1	HLA‐B40:01						
CoV_Prot_S_990‐1004	CoV_Prot_S_981‐1023	EVQIDRLITGRLQSL	1	HLA‐C03:04						
CoV_Prot_S_990‐1004	CoV_Prot_S_981‐1023	EVQIDRLITGRLQSL	1	DRB1_1501						
CoV_Prot_S_994‐1008	CoV_Prot_S_981‐1023	DRLITGRLQSLQTYV	2	HLA‐C03:04	HLA‐B27:05					
CoV_Prot_S_997‐1011	CoV_Prot_S_981‐1023	ITGRLQSLQTYVTQQ	2	HLA‐C15:05	HLA‐B27:05					
CoV_Prot_S_997‐1011	CoV_Prot_S_981‐1023	ITGRLQSLQTYVTQQ	1	DRB1_1501						
CoV_Prot_S_1000‐1014	CoV_Prot_S_981‐1023	RLQSLQTYVTQQLIR	1	DRB1_1501						
CoV_Prot_S_1055‐1068	CoV_Prot_S_1051‐1099	SAPHGVVFLHVTYV	1	DRB1_1201						
CoV_Prot_S_1056‐1070	CoV_Prot_S_1051‐1099	APHGVVFLHVTYVPA	2	DRB1_1201	DRB1_1501					
CoV_Prot_S_1097‐1111	CoV_Prot_S_1089‐1135	SNGTHWFVTQRNFYE	1	DRB1_0405						
CoV_Prot_S_1101‐1115	CoV_Prot_S_1089‐1135	HWFVTQRNFYEPQII	1	DRB1_0405						
CoV_Prot_S_1117‐1131	CoV_Prot_S_1089‐1135	TDNTFVSGNCDVVIG	1	HLA‐C05:01						
CoV_Prot_S_1141‐1155	CoV_Prot_S_1123‐1166	LQPELDSFKEELDKY	1	DRB1_1601						
CoV_Prot_S_1149‐1163	CoV_Prot_S_1123‐1166	KEELDKYFKNHTSPD	1	HLA‐B40:01						
CoV_Prot_S_1203‐1215	CoV_Prot_S_1195‐1230	LGKYEQYIKWPWY	1	HLA‐B44:02						
CoV_Prot_S_1205‐1219	CoV_Prot_S_1195‐1230	KYEQYIKWPWYIWLG	2	HLA‐B44:02	HLA‐A23:01					
CoV_Prot_S_1209‐1223	CoV_Prot_S_1195‐1230	YIKWPWYIWLGFIAG	2	HLA‐A02:01	HLA‐B35:01					
CoV_Prot_S_1214‐1228	CoV_Prot_S_1195‐1230	WYIWLGFIAGLIAIV	1	HLA‐A02:01						
CoV_Prot_S_1215‐1225	CoV_Prot_S_1195‐1230	YIWLGFIAGLI	1	HLA‐A02:01						
CoV_Prot_S_1217‐1231	CoV_Prot_S_1195‐1230	WLGFIAGLIAIVMVT	1	HLA‐A02:01						
CoV_Prot_S_1218‐1228	CoV_Prot_S_1195‐1230	LGFIAGLIAIV	1	HLA‐A02:01						
CoV_Prot_S_1220‐1230	CoV_Prot_S_1195‐1230	FIAGLIAIVMV	1	HLA‐A02:01						
CoV_Prot_Nuc_37‐51	CoV_Prot_Nuc_1‐51	SKQRRPQGLPNNTAS	1	DRB1_1501						
CoV_Prot_Nuc_49‐61	CoV_Prot_Nuc_40‐87	TASWFTALTQHGK	1	DQA10301‐DQB10201						
CoV_Prot_Nuc_73‐87	CoV_Prot_Nuc_40‐87	PINTNSSPDDQIGYY	1	DQA10501‐DQB10201						
CoV_Prot_N_102‐116	CoV_Prot_Nuc_78‐127	KDLSPRWYFYYLGTG	1	HLA‐B07:02						
CoV_Prot_N_105‐119	CoV_Prot_Nuc_78‐127	SPRWYFYYLGTGPEA	1	HLA‐B07:02						
CoV_Prot_Nuc_339‐353	CoV_Prot_Nuc_339‐387	LDDKDPNFKDQVILL	1	DQA10201‐DQB10201						
CoV_Prot_Nuc_341‐355	CoV_Prot_Nuc_339‐387	DKDPNFKDQVILLNK	2	DRB1_0408	DQA10201‐DQB10201					
CoV_Prot_N_360‐374	CoV_Prot_Nuc_339‐387	IDAYKTFPPTEPKKD	1	HLA‐A30:01						
CoV_Prot_N_405‐419	CoV_Prot_Nuc_377‐419	KQLQQSMSSADSTQA	1	HLA‐A01:01						
CoV_Prot_M_9‐23	CoV_Prot_M_1‐47	TVEELKKLLEQWNLV	1	HLA‐C01:02						
CoV_Prot_M_9‐23	CoV_Prot_M_1‐47	TVEELKKLLEQWNLV	1	DRB1_1101						
CoV_Prot_M_12‐26	CoV_Prot_M_1‐47	ELKKLLEQWNLVIGF	2	HLA‐A02:01	HLA‐A25:01					
CoV_Prot_M_21‐31	CoV_Prot_M_1‐47	NLVIGFLFLTW	1	HLA‐C01:02						
CoV_Prot_M_21‐31	CoV_Prot_M_1‐47	NLVIGFLFLTW	1	DQA10101‐DQB10501						
CoV_Prot_M_27‐37	CoV_Prot_M_1‐47	LFLTWICLLQF	1	HLA‐C02:02						
CoV_Prot_M_27‐37	CoV_Prot_M_1‐47	LFLTWICLLQF	1	DQA10101‐DQB10501						
CoV_Prot_M_42‐56	CoV_Prot_M_37‐73	RNRFLYIIKLIFLWL	1	HLA‐A23:01						
CoV_Prot_M_47‐57	CoV_Prot_M_37‐73	YIIKLIFLWLL	1	DRB1_1101						
CoV_Prot_M_60‐70	CoV_Prot_M_37‐73	VTLACFVLAAV	1	HLA‐A02:01						
CoV_Prot_M_137‐151	CoV_Prot_M_135‐183	ELVIGAVILRGHLRI	2	HLA‐A68:01	HLA‐A03:01					
CoV_Prot_M_144‐158	CoV_Prot_M_135‐183	ILRGHLRIAGHHLGR	1	HLA‐B08:01						
CoV_Prot_M_149‐163	CoV_Prot_M_135‐183	LRIAGHHLGRCDIKD	1	HLA‐A03:01						
CoV_Prot_M_149‐163	CoV_Prot_M_135‐183	LRIAGHHLGRCDIKD	3	DRB1_1301	DQA10201‐DQB10303					
CoV_Prot_M_157‐171	CoV_Prot_M_135‐183	GRCDIKDLPKEITVA	2	DRB1_0405						
CoV_Prot_M_165‐179	CoV_Prot_M_135‐183	PKEITVATSRTLSYY	1	HLA‐C02:02						
CoV_Prot_M_169‐183	CoV_Prot_M_173‐222	TVATSRTLSYYKLGA	1	DQA10201‐DQB10302						
CoV_Prot_M_173‐187	CoV_Prot_M_173‐222	SRTLSYYKLGASQRV	5	DRB1_1501	DRB1_0701	DQA10301‐DQB10301				
CoV_Prot_M_177‐191	CoV_Prot_M_173‐222	SYYKLGASQRVAGDS	4	DRB1_0701	DQA10301‐DQB10301					
CoV_Prot_M_193‐207	CoV_Prot_M_173‐222	FAAYSRYRIGNYKLN	3	HLA‐C02:02	HLA‐C14:02	HLA‐A30:01				
CoV_Prot_M_197‐211	CoV_Prot_M_173‐222	SRYRIGNYKLNTDHS	1	HLA‐A30:01						
CoV_Prot_M_197‐211	CoV_Prot_M_173‐222	SRYRIGNYKLNTDHS	1	DRB1_1501						
CoV_Prot_M_201‐215	CoV_Prot_M_173‐222	IGNYKLNTDHSSSSD	3	DRB1_0405	DRB1_0408	DRB1_0401				
CoV_Prot_M_208‐222	CoV_Prot_M_173‐222	TDHSSSSDNIALLVQ	1	HLA‐A01:01						
CoV_Prot_M_208‐222	CoV_Prot_M_173‐222	TDHSSSSDNIALLVQ	1	DRB1_0401						

*Note*: Rows refer to individual 15‐mer peptides derived from the SARS‐CoV‐2 Spike (S), Nucleocapsid (N), or Membrane (M) protein (1st column), as well as their ancestor peptide pools (2nd column). The specific peptide sequence (3rd column) and the number of reactive donors found four each of the peptides after *in vitro* stimulation (4th column) are indicated. Likewise, the likely HLA restriction of the peptides, that were predicted by NetMHCpan given the HLA allotypes by reactive donors, are listed (5th column).

**TABLE 3:** 15‐mer peptides with predicted immunogenic capacity.

15‐mer peptide	Ancestor peptide pool	Peptide sequence	Predicted HLA restriction						
CoV_Prot_S_13‐27	CoV_Prot_S_1‐51	SQCVNLTTRTQLPPA	DRB1_1401						
CoV_Prot_S_18‐32	CoV_Prot_S_1‐51	LTTRTQLPPAYTNSF	HLA‐C07:02	HLA‐B35:03	HLA‐C07:01				
CoV_Prot_S_21‐35	CoV_Prot_S_1‐51	RTQLPPAYTNSFTRG	HLA‐B35:03						
CoV_Prot_S_24‐38	CoV_Prot_S_1‐51	LPPAYTNSFTRGVYY	HLA‐B35:03	HLA‐C12:03	HLA‐A29:02	HLA‐C15:02			
CoV_Prot_S_29‐43	CoV_Prot_S_1‐51	TNSFTRGVYYPDKVF	HLA‐C12:03	HLA‐A29:02					
CoV_Prot_S_33‐47	CoV_Prot_S_1‐51	TRGVYYPDKVFRSSV	HLA‐A31:01	DRB1_0101	DRB1_1302	DRB1_1101	DRB1_0301		
CoV_Prot_S_37‐51	CoV_Prot_S_1‐51	YYPDKVFRSSVLHST	HLA‐B14:01	DRB1_1501					
CoV_Prot_S_41‐55	CoV_Prot_S_41‐91	KVFRSSVLHSTQDLF	HLA‐A03:01						
CoV_Prot_S_44‐58	CoV_Prot_S_41‐91	PFFSNVTWFHAIHVS	DRB1_1501						
CoV_Prot_S_65‐79	CoV_Prot_S_41‐91	FHAIHVSGTNGTKRF	DRB1_1302						
CoV_Prot_S_72‐86	CoV_Prot_S_41‐91	GTNGTKRFDNPVLPF	HLA‐C04:01	HLA‐C07:02					
CoV_Prot_S_77‐91	CoV_Prot_S_41‐91	KRFDNPVLPFNDGVY	HLA‐C04:01	HLA‐C07:02					
CoV_Prot_S_81‐95	CoV_Prot_S_81‐130	NPVLPFNDGVYFAST	HLA‐B35:03	DRB1_1501					
CoV_Prot_S_85‐99	CoV_Prot_S_81‐130	PFNDGVYFASTEKSN	HLA‐A03:01	HLA‐DQA10401‐DQB10601	HLA‐DQA10401‐DQB10402	HLA‐DQA10103‐DQB10603	DRB1_0404		
CoV_Prot_S_89‐103	CoV_Prot_S_81‐130	GVYFASTEKSNIIRG	HLA‐A03:01						
CoV_Prot_S_93‐107	CoV_Prot_S_81‐130	ASTEKSNIIRGWIFG	HLA‐C15:02						
CoV_Prot_S_105‐119	CoV_Prot_S_81‐130	IFGTTLDSKTQSLLI	HLA‐A02:01	HLA‐C07:04	HLA‐C04:01	HLA‐B08:01	DRB1_0301		
CoV_Prot_S_109‐123	CoV_Prot_S_81‐130	TLDSKTQSLLIVNNA	HLA‐A02:01	HLA‐C07:04	HLA‐C04:01	HLA‐B08:01			
CoV_Prot_S_121‐135	CoV_Prot_S_121‐175	NNATNVVIKVCEFQF	HLA‐C12:03	HLA‐C12:03	HLA‐B51:01				
CoV_Prot_S_138‐152	CoV_Prot_S_121‐175	DPFLGVYYHKNNKSW	HLA‐A03:01	HLA‐A24:02	HLA‐DQA10103‐DQB10402	DRB1_1301			
CoV_Prot_S_150‐164	CoV_Prot_S_121‐175	KSWMESEFRVYSSAN	HLA‐B50:01	DRB1_0301	DRB1_1101				
CoV_Prot_S_154‐168	CoV_Prot_S_121‐175	ESEFRVYSSANNCTF	HLA‐B50:01	DRB1_1501	DRB1_0404				
CoV_Prot_S_157‐171	CoV_Prot_S_121‐175	FRVYSSANNCTFEYV	HLA‐C16:01	HLA‐A01:01					
CoV_Prot_S_161‐175	CoV_Prot_S_121‐175	SSANNCTFEYVSQPF	HLA‐A01:01						
CoV_Prot_S_165‐179	CoV_Prot_S_165‐216	NCTFEYVSQPFLMDL	HLA‐C07:02						
CoV_Prot_S_181‐195	CoV_Prot_S_165‐216	GKQGNFKNLREFVFK	DRB1_1201						
CoV_Prot_S_198‐212	CoV_Prot_S_165‐216	DGYFKIYSKHTPINL	HLA‐C03:03	HLA‐C07:04	DRB1_0801	HLA‐DQA10301‐DQB10503	DRB1_1101		
CoV_Prot_S_202‐216	CoV_Prot_S_165‐216	KIYSKHTPINLVRDL	HLA‐B07:02	HLA‐C03:03	HLA‐C07:04				
CoV_Prot_S_205‐219	CoV_Prot_S_205‐255	SKHTPINLVRDLPQG	HLA‐DQA10103‐DQB10603	HLA‐DQA10101‐DQB10603	HLA‐DQA10101‐DQB10301	HLA‐DQA10101‐DQB10501			
CoV_Prot_S_217‐231	CoV_Prot_S_205‐255	PQGFSALEPLVDLPI	HLA‐C03:03						
CoV_Prot_S_221‐235	CoV_Prot_S_205‐255	SALEPLVDLPIGINI	HLA‐C03:03						
CoV_Prot_S_233‐247	CoV_Prot_S_205‐255	INITRFQTLLALHRS	HLA‐C07:01						
CoV_Prot_S_236‐250	CoV_Prot_S_205‐255	TRFQTLLALHRSYLT	HLA‐C07:01						
CoV_Prot_S_245‐259	CoV_Prot_S_245‐295	HRSYLTPGDSSSGWT	HLA‐A02:01						
CoV_Prot_S_249‐263	CoV_Prot_S_245‐295	LTPGDSSSGWTAGAA	HLA‐C12:03						
CoV_Prot_S_253‐267	CoV_Prot_S_245‐295	DSSSGWTAGAAAYYV	HLA‐DQA10501‐DQB10301						
CoV_Prot_S_257‐271	CoV_Prot_S_245‐295	GWTAGAAAYYVGYLQ	HLA‐DQA10501‐DQB10301						
CoV_Prot_S_265‐279	CoV_Prot_S_245‐295	YYVGYLQPRTFLLKY	HLA‐A02:01						
CoV_Prot_S_269‐283	CoV_Prot_S_245‐295	YLQPRTFLLKYNENG	HLA‐A02:01						
CoV_Prot_S_273‐287	CoV_Prot_S_245‐295	RTFLLKYNENGTITD	DRB1_0301						
CoV_Prot_S_302‐316	CoV_Prot_S_285‐327	TLKSFTVEKGIYQTS	DRB1_1101						
CoV_Prot_S_322‐335	CoV_Prot_S_313‐351	PTESIVRFPNITNL	HLA‐C07:02						
CoV_Prot_S_323‐335	CoV_Prot_S_313‐351	TESIVRFPNITNL	HLA‐C07:02						
CoV_Prot_S_324‐338	CoV_Prot_S_313‐351	ESIVRFPNITNLCPF	HLA‐C07:02						
CoV_Prot_S_326‐338	CoV_Prot_S_313‐351	IVRFPNITNLCPF	HLA‐C07:02						
CoV_Prot_S_342‐356	CoV_Prot_S_342‐390	FNATRFASVYAWNRK	HLA‐C07:01	HLA‐B27:05	HLA‐DQA10401‐DQB10402	HLA‐DQA10103‐DQB10402			
CoV_Prot_S_345‐359	CoV_Prot_S_342‐390	TRFASVYAWNRKRIS	HLA‐A03:01	HLA‐A74:03	DRB1_1301				
CoV_Prot_S_349‐363	CoV_Prot_S_342‐390	SVYAWNRKRISNCVA	HLA‐A03:01	HLA‐A74:03					
CoV_Prot_S_357‐371	CoV_Prot_S_342‐390	RISNCVADYSVLYNS	HLA‐A01:01						
CoV_Prot_S_362‐376	CoV_Prot_S_313‐351	VADYSVLYNSASFST	DRB1_0401	DRB1_0101	DRB1_1101				
CoV_Prot_S_364‐378	CoV_Prot_S_342‐390	DYSVLYNSASFSTFK	HLA‐A11:01	DRB1_1302					
CoV_Prot_S_369‐383	CoV_Prot_S_342‐390	YNSASFSTFKCYGVS	HLA‐A11:01	DRB1_1501					
CoV_Prot_S_373‐387	CoV_Prot_S_342‐390	SFSTFKCYGVSPTKL	HLA‐A03:01						
CoV_Prot_S_376‐390	CoV_Prot_S_342‐390	TFKCYGVSPTKLNDL	HLA‐A03:01						
CoV_Prot_S_397‐411	CoV_Prot_S_381‐431	ADSFVIRGDEVRQIA	HLA‐C06:02						
CoV_Prot_S_401‐415	CoV_Prot_S_381‐431	VIRGDEVRQIAPGQT	HLA‐C06:02						
CoV_Prot_S_405‐419	CoV_Prot_S_381‐431	DEVRQIAPGQTGKIA	HLA‐A03:01						
CoV_Prot_S_413‐427	CoV_Prot_S_381‐431	GQTGKIADYNYKLPD	HLA‐A02:01						
CoV_Prot_S_417‐431	CoV_Prot_S_381‐431	KIADYNYKLPDDFTG	HLA‐A02:01						
CoV_Prot_S_430‐444	CoV_Prot_S_421‐455	TGCVIAWNSNNLDSK	HLA‐DQA10103‐DQB10501	HLA‐DQA10101‐DQB10501	DRB1_1501				
CoV_Prot_S_438‐452	CoV_Prot_S_421‐455	SNNLDSKVGGNYNYL	HLA‐C07:02						
CoV_Prot_S_441‐455	CoV_Prot_S_421‐455	LDSKVGGNYNYLYRL	HLA‐C07:02						
CoV_Prot_S_445‐459	CoV_Prot_S_441‐475	VGGNYNYLYRLFRKS	DRB1_1101						
CoV_Prot_S_453‐467	CoV_Prot_S_441‐475	YRLFRKSNLKPFERD	HLA‐A31:01						
CoV_Prot_S_461‐475	CoV_Prot_S_441‐475	LKPFERDISTEIYQA	HLA‐B52:01	HLA‐B50:01	DRB1_0101	HLA‐B52:01	HLA‐B56:01		
CoV_Prot_S_464‐478	CoV_Prot_S_461‐508	FERDISTEIYQAGST	HLA‐B52:01						
CoV_Prot_S_486‐500	CoV_Prot_S_461‐508	FNCYFPLQSYGFQPT	HLA‐C07:02						
CoV_Prot_S_489‐503	CoV_Prot_S_461‐508	YFPLQSYGFQPTNGV	HLA‐C07:02						
CoV_Prot_S_494‐508	CoV_Prot_S_461‐508	SYGFQPTNGVGYQPY	HLA‐B15:01	DRB1_1302					
CoV_Prot_S_500‐513	CoV_Prot_S_494‐531	TNGVGYQPYRVVVL	HLA‐C01:02						
CoV_Prot_S_501‐515	CoV_Prot_S_494‐531	NGVGYQPYRVVVLSF	HLA‐C01:02						
CoV_Prot_S_502‐515	CoV_Prot_S_494‐531	GVGYQPYRVVVLSF	HLA‐C01:02						
CoV_Prot_S_505‐519	CoV_Prot_S_494‐531	YQPYRVVVLSFELLH	HLA‐B52:01	HLA‐C01:02	HLA‐C01:02	HLA‐B08:01			
CoV_Prot_S_521‐535	CoV_Prot_S_521‐567	PATVCGPKKSTNLVK	HLA‐C01:02						
CoV_Prot_S_526‐539	CoV_Prot_S_521‐567	GPKKSTNLVKNKCV	HLA‐A03:01						
CoV_Prot_S_529‐543	CoV_Prot_S_521‐567	KSTNLVKNKCVNFNF	HLA‐A03:01	DRB1_1302					
CoV_Prot_S_541‐555	CoV_Prot_S_521‐567	FNFNGLTGTGVLTES	DRB1_0101						
CoV_Prot_S_543‐557	CoV_Prot_S_521‐567	FNGLTGTGVLTESNK	HLA‐DQA10103‐DQB10601						
CoV_Prot_S_553‐567	CoV_Prot_S_521‐567	TESNKKFLPFQQFGR	DRB1_1501						
CoV_Prot_S_557‐571	CoV_Prot_S_557‐607	KKFLPFQQFGRDIAD	DRB1_1501						
CoV_Prot_S_573‐587	CoV_Prot_S_557‐607	TDAVRDPQTLEILDI	HLA‐C07:02						
CoV_Prot_S_581‐595	CoV_Prot_S_557‐607	TLEILDITPCSFGGV	HLA‐C05:01						
CoV_Prot_S_621‐635	CoV_Prot_S_597‐635	PVAIHADQLTPTWRV	DRB1_0301						
CoV_Prot_S_656‐670	CoV_Prot_S_597‐635	VNNSYECDIPIGAGI	HLA‐B50:01	DRB1_0301	DRB1_0401				
CoV_Prot_S_660‐674	CoV_Prot_S_597‐635	YECDIPIGAGICASY	HLA‐B50:01	HLA‐B56:01					
CoV_Prot_S_673‐687	CoV_Prot_S_665‐707	SYQTQTNSPRRARSV	HLA‐A68:01						
CoV_Prot_S_678‐692	CoV_Prot_S_625‐674	TNSPRRARSVASQSI	HLA‐B07:02	HLA‐B07:02	HLA‐B56:01				
CoV_Prot_S_683‐696	CoV_Prot_S_625‐674	RARSVASQSIIAYT	HLA‐C12:02	HLA‐C12:03	DRB1_0301				
CoV_Prot_S_685‐699	CoV_Prot_S_625‐674	RSVASQSIIAYTMSL	HLA‐C12:02	HLA‐C12:03					
CoV_Prot_S_689‐703	CoV_Prot_S_665‐707	SQSIIAYTMSLGAEN	HLA‐DQA10102‐DQB10503						
CoV_Prot_S_702‐716	CoV_Prot_S_698‐747	ENSVAYSNNSIAIPT	HLA‐DQA10103‐DQB10402	HLA‐DQA10103‐DQB10601	HLA‐DQA10102‐DQB10503	DRB1_1501			
CoV_Prot_S_706‐720	CoV_Prot_S_698‐747	AYSNNSIAIPTNFTI	HLA‐B52:01						
CoV_Prot_S_709‐723	CoV_Prot_S_698‐747	NNSIAIPTNFTISVT	HLA‐B52:01	HLA‐B55:01	HLA‐B51:01				
CoV_Prot_S_713‐727	CoV_Prot_S_698‐747	AIPTNFTISVTTEIL	HLA‐C05:01	HLA‐B51:01					
CoV_Prot_S_719‐733	CoV_Prot_S_698‐747	TISVTTEILPVSMTK	HLA‐A03:01	HLA‐DQA10501‐DQB10201					
CoV_Prot_S_722‐736	CoV_Prot_S_698‐747	VTTEILPVSMTKTSV	HLA‐A03:01						
CoV_Prot_S_725‐739	CoV_Prot_S_698‐747	EILPVSMTKTSVDCT	HLA‐A03:01						
CoV_Prot_S_745‐759	CoV_Prot_S_737‐779	DSTECSNLLLQYGSF	HLA‐C05:01						
CoV_Prot_S_749‐763	CoV_Prot_S_737‐779	CSNLLLQYGSFCTQL	HLA‐C14:02	DRB1_1501					
CoV_Prot_S_753‐765	CoV_Prot_S_737‐779	LLQYGSFCTQLNR	HLA‐A11:01	HLA‐C14:02					
CoV_Prot_S_756‐767	CoV_Prot_S_737‐779	YGSFCTQLNRAL	HLA‐A11:01						
CoV_Prot_S_757‐770	CoV_Prot_S_737‐779	GSFCTQLNRALTGI	HLA‐A11:01						
CoV_Prot_S_765‐779	CoV_Prot_S_737‐779	RALTGIAVEQDKNTQ	HLA‐DQA10401‐DQB10402	HLA‐DQA10501‐DQB10201	HLA‐DQA10102‐DQB10602	HLA‐DQA10501‐DQB10602			
CoV_Prot_S_769‐783	CoV_Prot_S_769‐813	GIAVEQDKNTQEVFA	HLA‐B38:01	DRB1_0301					
CoV_Prot_S_773‐787	CoV_Prot_S_769‐813	EQDKNTQEVFAQVKQ	HLA‐B38:01						
CoV_Prot_S_777‐791	CoV_Prot_S_769‐813	NTQEVFAQVKQIYKT	HLA‐A29:01	HLA‐C14:02	DRB1_1401				
CoV_Prot_S_781‐795	CoV_Prot_S_769‐813	VFAQVKQIYKTPPIK	HLA‐A11:01	HLA‐A03:01	HLA‐C14:02				
CoV_Prot_S_785‐799	CoV_Prot_S_769‐813	VKQIYKTPPIKDFGG	HLA‐A11:01	HLA‐A03:01	HLA‐C07:01				
CoV_Prot_S_788‐802	CoV_Prot_S_769‐813	IYKTPPIKDFGGFNF	HLA‐C07:01						
CoV_Prot_S_792‐806	CoV_Prot_S_769‐813	PPIKDFGGFNFSQIL	DRB1_1501						
CoV_Prot_S_796‐810	CoV_Prot_S_769‐813	DFGGFNFSQILPDPS	HLA‐DQA10401‐DQB10402						
CoV_Prot_S_802‐816	CoV_Prot_S_802‐852	FSQILPDPSKPSKRS	HLA‐DQA10301‐DQB10503	DRB1_1302	DRB1_0401	DRB1_1301	DRB1_1601		
CoV_Prot_S_817‐831	CoV_Prot_S_802‐852	FIEDLLFNKVTLADA	HLA‐A02:01	HLA‐B08:01					
CoV_Prot_S_821‐835	CoV_Prot_S_802‐852	LLFNKVTLADAGFIK	HLA‐A02:01						
CoV_Prot_S_825‐839	CoV_Prot_S_802‐852	KVTLADAGFIKQYGD	HLA‐B44:02						
CoV_Prot_S_829‐843	CoV_Prot_S_802‐852	ADAGFIKQYGDCLGD	HLA‐B44:02	HLA‐DQA10103‐DQB10501	DRB1_1501				
CoV_Prot_S_841‐855	CoV_Prot_S_841‐891	LGDIAARDLICAQKF	HLA‐C05:01						
CoV_Prot_S_847‐861	CoV_Prot_S_841‐891	RDLICAQKFNGLTVL	HLA‐B14:02						
CoV_Prot_S_852‐866	CoV_Prot_S_841‐891	AQKFNGLTVLPPLLT	HLA‐B14:02						
CoV_Prot_S_860‐874	CoV_Prot_S_841‐891	VLPPLLTDEMIAQYT	DRB1_0301						
CoV_Prot_S_865‐879	CoV_Prot_S_841‐891	LTDEMIAQYTSALLA	HLA‐B50:01	HLA‐C03:04	HLA‐DQA10101‐DQB10501	DRB1_1501			
CoV_Prot_S_869‐883	CoV_Prot_S_841‐891	MIAQYTSALLAGTIT	HLA‐B08:01	HLA‐B50:01	HLA‐C03:04	HLA‐DQA10401‐DQB10601	HLA‐DQA10102‐DQB10602		
CoV_Prot_S_881‐895	CoV_Prot_S_881‐927	TITSGWTFGAGAALQ	HLA‐C03:04	HLA‐C03:03					
CoV_Prot_S_885‐899	CoV_Prot_S_881‐927	GWTFGAGAALQIPFA	HLA‐C03:04	HLA‐C03:03	HLA‐DQA10501‐DQB10301	DRB1_0101			
CoV_Prot_S_889‐902	CoV_Prot_S_881‐927	GAGAALQIPFAMQM	HLA‐B52:01						
CoV_Prot_S_892‐906	CoV_Prot_S_881‐927	AALQIPFAMQMAYRF	HLA‐B52:01						
CoV_Prot_S_896‐910	CoV_Prot_S_881‐927	IPFAMQMAYRFNGIG	DRB1_1101						
CoV_Prot_S_902‐915	CoV_Prot_S_881‐927	MAYRFNGIGVTQNV	HLA‐B39:06						
CoV_Prot_S_904‐918	CoV_Prot_S_881‐927	YRFNGIGVTQNVLYE	HLA‐B39:06						
CoV_Prot_S_912‐923	CoV_Prot_S_881‐927	TQNVLYENQKLI	HLA‐A02:01	DRB1_1501					
CoV_Prot_S_915‐927	CoV_Prot_S_881‐927	VLYENQKLIANQF	HLA‐A02:01	HLA‐B15:01					
CoV_Prot_S_933‐947	CoV_Prot_S_917‐964	KIQDSLSSTASALGK	HLA‐A11:01						
CoV_Prot_S_937‐951	CoV_Prot_S_917‐964	SLSSTASALGKLQDV	HLA‐A11:01						
CoV_Prot_S_945‐959	CoV_Prot_S_917‐964	LGKLQDVVNQNAQAL	HLA‐C08:02	HLA‐C03:04					
CoV_Prot_S_948‐962	CoV_Prot_S_917‐964	LQDVVNQNAQALNTL	HLA‐C08:02	HLA‐C03:04	DRB1_0301	DRB1_1302			
CoV_Prot_S_959‐973	CoV_Prot_S_955‐991	LNTLVKQLSSNFGAI	DRB1_1501						
CoV_Prot_S_963‐976	CoV_Prot_S_955‐991	VKQLSSNFGAISSV	HLA‐C15:02						
CoV_Prot_S_967‐981	CoV_Prot_S_955‐991	SSNFGAISSVLNDIL	HLA‐DQA10501‐DQB10201						
CoV_Prot_S_972‐984	CoV_Prot_S_955‐991	AISSVLNDILSRL	HLA‐A11:01	HLA‐A02:01					
CoV_Prot_S_975‐987	CoV_Prot_S_955‐991	SVLNDILSRLDKV	HLA‐A11:01	HLA‐A02:01					
CoV_Prot_S_981‐995	CoV_Prot_S_981‐1023	LSRLDKVEAEVQIDR	HLA‐A02:01						
CoV_Prot_S_986‐1000	CoV_Prot_S_981‐1023	KVEAEVQIDRLITGR	DRB1_0301						
CoV_Prot_S_994‐1008	CoV_Prot_S_981‐1023	DRLITGRLQSLQTYV	HLA‐C07:02						
CoV_Prot_S_997‐1011	CoV_Prot_S_981‐1023	ITGRLQSLQTYVTQQ	HLA‐C07:02						
CoV_Prot_S_1000‐1014	CoV_Prot_S_981‐1023	RLQSLQTYVTQQLIR	HLA‐B52:01						
CoV_Prot_S_1004‐1018	CoV_Prot_S_981‐1023	LQTYVTQQLIRAAEI	HLA‐B52:01						
CoV_Prot_S_1009‐1023	CoV_Prot_S_981‐1023	TQQLIRAAEIRASAN	HLA‐DQA10103‐DQB10603	HLA‐DQA10101‐DQB10603	HLA‐DQA10102‐DQB10602	HLA‐DQA10301‐DQB10602	HLA‐DQA10102‐DQB10503	HLA‐DQA10301‐DQB10503	
CoV_Prot_S_1012‐1026	CoV_Prot_S_1012‐1059	LIRAAEIRASANLAA	HLA‐B39:06	HLA‐DQA10102‐DQB10602					
CoV_Prot_S_1014‐1028	CoV_Prot_S_1012‐1059	RAAEIRASANLAATK	HLA‐B39:06	HLA‐DQA10301‐DQB10602	HLA‐DQA10102‐DQB10503	HLA‐DQA10301‐DQB10503	DRB1_1501	IRASANLAA	DRB1_0404
CoV_Prot_S_1045‐1059	CoV_Prot_S_1012‐1059	KGYHLMSFPQSAPHG	HLA‐A02:01						
CoV_Prot_S_1061‐1075	CoV_Prot_S_1051‐1099	VFLHVTYVPAQEKNF	HLA‐A11:01	HLA‐A03:01					
CoV_Prot_S_1066‐1080	CoV_Prot_S_1051‐1099	TYVPAQEKNFTTAPA	HLA‐B45:01						
CoV_Prot_S_1069‐1083	CoV_Prot_S_1051‐1099	PAQEKNFTTAPAICH	HLA‐B45:01						
CoV_Prot_S_1073‐1087	CoV_Prot_S_1051‐1099	KNFTTAPAICHDGKA	HLA‐DQA10103‐DQB10601						
CoV_Prot_S_1077‐1091	CoV_Prot_S_1051‐1099	TAPAICHDGKAHFPR	DRB1_0301	DRB1_1401					
CoV_Prot_S_1093‐1107	CoV_Prot_S_1089‐1135	GVFVSNGTHWFVTQR	HLA‐A11:01	HLA‐A03:01					
CoV_Prot_S_1097‐1111	CoV_Prot_S_1089‐1135	SNGTHWFVTQRNFYE	HLA‐A11:01	HLA‐A03:01	DRB1_0301	HLA‐DQA10102‐DQB10503			
CoV_Prot_S_1131‐1145	CoV_Prot_S_1123‐1166	GIVNNTVYDPLQPEL	HLA‐C04:01	HLA‐C05:01	HLA‐C06:02	HLA‐C01:02			
CoV_Prot_S_1136‐1149	CoV_Prot_S_1123‐1166	TVYDPLQPELDSFK	HLA‐C04:01	HLA‐C05:01	HLA‐C06:02	HLA‐C01:02			
CoV_Prot_S_1167‐1181	CoV_Prot_S_1157‐1205	GDISGINASVVNIQK	HLA‐DQA10401‐DQB10601	HLA‐DQA10103‐DQB10601	HLA‐DQA10501‐DQB10301				
CoV_Prot_S_1171‐1185	CoV_Prot_S_1157‐1205	GINASVVNIQKEIDR	DRB1_0801						
CoV_Prot_S_1175‐1189	CoV_Prot_S_1157‐1205	SVVNIQKEIDRLNEV	HLA‐B40:01						
CoV_Prot_S_1179‐1193	CoV_Prot_S_1157‐1205	IQKEIDRLNEVAKNL	HLA‐B40:01						
CoV_Prot_S_1181‐1195	CoV_Prot_S_1157‐1205	KEIDRLNEVAKNLNE	HLA‐B40:01						
CoV_Prot_S_1186‐1200	CoV_Prot_S_1157‐1205	LNEVAKNLNESLIDL	HLA‐C12:02						
CoV_Prot_S_1189‐1203	CoV_Prot_S_1157‐1205	VAKNLNESLIDLQEL	HLA‐C12:02						
CoV_Prot_S_1197‐1211	CoV_Prot_S_1195‐1230	LIDLQELGKYEQYIK	HLA‐B44:02	HLA‐B18:01	DRB1_0801	DRB1_0401			
CoV_Prot_S_1217‐1231	CoV_Prot_S_1195‐1230	WLGFIAGLIAIVMVT	HLA‐DQA10501‐DQB10301						
CoV_Prot_S_1255‐1268	CoV_Prot_S_1222‐1273	KFDEDDSEPVLKGV	HLA‐B38:01	DRB1_0401					
CoV_Prot_S_1257‐1271	CoV_Prot_S_1222‐1273	DEDDSEPVLKGVKLH	HLA‐B35:03						
CoV_Prot_S_1259‐1273	CoV_Prot_S_1222‐1273	DDSEPVLKGVKLHYT	HLA‐C02:02	HLA‐B15:01	HLA‐B35:03	HLA‐DQA10103‐DQB10601			
CoV_Prot_Nuc_5‐19	CoV_Prot_N_1‐51	GPQNQRNAPRITFGG	HLA‐DQA10102‐DQB10602	HLA‐B38:01					
CoV_Prot_Nuc_10‐24	CoV_Prot_N_1‐51	RNAPRITFGGPSDST	HLA‐DQA10401‐DQB10601						
CoV_Prot_Nuc_13‐27	CoV_Prot_N_1‐51	PRITFGGPSDSTGSN	HLA‐DQA10401‐DQB10601						
CoV_Prot_Nuc_37‐51	CoV_Prot_N_1‐51	SKQRRPQGLPNNTAS	HLA‐B07:02						
CoV_Prot_Nuc_54‐68	CoV_Prot_N_40‐87	TALTQHGKEDLKFPR	HLA‐B38:01						
CoV_Prot_Nuc_57‐71	CoV_Prot_N_40‐87	TQHGKEDLKFPRGQG	HLA‐B38:01						
CoV_Prot_Nuc_78‐92	CoV_Prot_N_78‐127	SSPDDQIGYYRRATR	HLA‐C04:01						
CoV_Prot_Nuc_81‐95	CoV_Prot_N_78‐127	DDQIGYYRRATRRIR	HLA‐A24:02						
CoV_Prot_Nuc_85‐99	CoV_Prot_N_78‐127	GYYRRATRRIRGGDG	HLA‐A24:02						
CoV_Prot_Nuc_113‐127	CoV_Prot_N_78‐127	LGTGPEAGLPYGANK	HLA‐DQA10401‐DQB10601						
CoV_Prot_Nuc_149‐163	CoV_Prot_N_117‐167	RNPANNAAIVLQLPQ	HLA‐DQA10103‐DQB10603						
CoV_Prot_Nuc_158‐172	CoV_Prot_N_158‐203	VLQLPQGTTLPKGFY	HLA‐C07:02						
CoV_Prot_Nuc_197‐211	CoV_Prot_N_194‐234	STPGSSRGTSPARMA	HLA‐DQA10101‐DQB10603	HLA‐DQA10101‐DQB10301					
CoV_Prot_Nuc_217‐230	CoV_Prot_N_194‐234	AALALLLLDRLNQL	HLA‐A02:01	DRB1_1101					
CoV_Prot_Nuc_220‐234	CoV_Prot_N_194‐234	ALLLLDRLNQLESKM	HLA‐A02:01						
CoV_Prot_Nuc_243‐256	CoV_Prot_N_224‐274	GQTVTKKSAAEASK	HLA‐DQA10103‐DQB10402	HLA‐DQA10501‐DQB10301					
CoV_Prot_Nuc_253‐267	CoV_Prot_N_224‐274	EASKKPRQKRTATKA	HLA‐B07:02						
CoV_Prot_Nuc_257‐271	CoV_Prot_N_224‐274	KPRQKRTATKAYNVT	HLA‐B07:02						
CoV_Prot_Nuc_261‐274	CoV_Prot_N_224‐274	KRTATKAYNVTQAF	HLA‐C03:03	HLA‐C06:02					
CoV_Prot_Nuc_265‐278	CoV_Prot_N_265‐314	TKAYNVTQAFGRRG	HLA‐C12:02						
CoV_Prot_Nuc_293‐307	CoV_Prot_N_265‐314	RQGTDYKHWPQIAQF	HLA‐B38:01	HLA‐C07:02					
CoV_Prot_Nuc_297‐311	CoV_Prot_N_265‐314	DYKHWPQIAQFAPSA	HLA‐B38:01	HLA‐C07:02					
CoV_Prot_Nuc_300‐314	CoV_Prot_N_265‐314	HWPQIAQFAPSASAF	HLA‐DQA10103‐DQB10402						
CoV_Prot_Nuc_318‐331	CoV_Prot_N_305‐347	SRIGMEVTPSGTWL	HLA‐B44:03						
CoV_Prot_Nuc_321‐335	CoV_Prot_N_305‐347	GMEVTPSGTWLTYTG	HLA‐B44:03						
CoV_Prot_Nuc_333‐347	CoV_Prot_N_305‐347	YTGAIKLDDKDPNFK	HLA‐C04:01						
CoV_Prot_Nuc_357‐371	CoV_Prot_N_339‐387	YKTFPPTEPKKDKKK	HLA‐A30:01						
CoV_Prot_Nuc_357‐371	CoV_Prot_N_339‐387	IDAYKTFPPTEPKKD	DRB1_0408	DRB1_0701					
CoV_Prot_M_1‐15	CoV_Prot_M_1‐47	MADSNGTITVEELKK	HLA‐DQA10103‐DQB10603						
CoV_Prot_M_9‐23	CoV_Prot_M_1‐47	TVEELKKLLEQWNLV	HLA‐A02:01						
CoV_Prot_M_12‐26	CoV_Prot_M_1‐47	ELKKLLEQWNLVIGF	HLA‐B52:01	HLA‐A02:01					
CoV_Prot_M_14‐29	CoV_Prot_M_1‐47	KKLLEQWNLVIGFLFL	HLA‐B52:01	HLA‐A02:01					
CoV_Prot_M_34‐47	CoV_Prot_M_1‐47	LLQFAYANRNRFLY	HLA‐C03:03	HLA‐C12:03	HLA‐C02:02	DRB1_1101			
CoV_Prot_M_37‐51	CoV_Prot_M_37‐73	FAYANRNRFLYIIKL	HLA‐C12:02	HLA‐C03:03					
CoV_Prot_M_39‐53	CoV_Prot_M_37‐73	YANRNRFLYIIKLIF	HLA‐A29:01	HLA‐DQA10101‐DQB10501					
CoV_Prot_M_42‐56	CoV_Prot_M_37‐73	RNRFLYIIKLIFLWL	DRB1_1101	DRB1_0301					
CoV_Prot_M_65‐76	CoV_Prot_M_65‐105	FVLAAVYRINWI	HLA‐A02:01						
CoV_Prot_M_72‐85	CoV_Prot_M_65‐105	RINWITGGIAIAMA	HLA‐DQA10501‐DQB10301	HLA‐DQA10501‐DQB10201					
CoV_Prot_M_89‐103	CoV_Prot_M_65‐105	GLMWLSYFIASFRLF	HLA‐A23:01						
CoV_Prot_M_93‐105	CoV_Prot_M_65‐105	LSYFIASFRLFAR	HLA‐A23:01						
CoV_Prot_M_97‐111	CoV_Prot_M_97‐147	IASFRLFARTRSMWS	DRB1_0801						
CoV_Prot_M_102‐116	CoV_Prot_M_97‐147	LFARTRSMWSFNPET	HLA‐C07:01						
CoV_Prot_M_105‐119	CoV_Prot_M_97‐147	RTRSMWSFNPETNIL	HLA‐C04:01						
CoV_Prot_M_110‐124	CoV_Prot_M_97‐147	WSFNPETNILLNVPL	HLA‐C04:01	DRB1_0101					
CoV_Prot_M_117‐131	CoV_Prot_M_97‐147	NILLNVPLHGTILTR	HLA‐C01:02						
CoV_Prot_M_120‐134	CoV_Prot_M_97‐147	LNVPLHGTILTRPLL	HLA‐C01:02						
CoV_Prot_M_129‐143	CoV_Prot_M_97‐147	LTRPLLESELVIGAV	HLA‐B45:01						
CoV_Prot_M_133‐147	CoV_Prot_M_97‐147	LLESELVIGAVILRG	HLA‐B45:01	DRB1_0301					
CoV_Prot_M_135‐148	CoV_Prot_M_135‐183	ESELVIGAVILRGH	HLA‐DQA10401‐DQB10601	HLA‐DQA10102‐DQB10602					
CoV_Prot_M_165‐179	CoV_Prot_M_135‐183	PKEITVATSRTLSYY	HLA‐C03:03	HLA‐A01:01	HLA‐DQA10103‐DQB10402	DRB1_0101	DRB1_0701		
CoV_Prot_M_169‐183	CoV_Prot_M_135‐183	TVATSRTLSYYKLGA	HLA‐C07:02	HLA‐A01:01	HLA‐C02:02				
CoV_Prot_M_173‐187	CoV_Prot_M_173‐222	SRTLSYYKLGASQRV	HLA‐C07:02	HLA‐C06:02					
CoV_Prot_M_177‐191	CoV_Prot_M_173‐222	SYYKLGASQRVAGDS	HLA‐DQA10501‐DQB10301	DRB1_0101					
CoV_Prot_M_201‐215	CoV_Prot_M_173‐222	IGNYKLNTDHSSSSD	HLA‐DQA10103‐DQB10402	HLA‐DQA10101‐DQB10603	HLA‐DQA10103‐DQB10603	HLA‐DQA10102‐DQB10201			
CoV_Prot_M_205‐219	CoV_Prot_M_173‐222	KLNTDHSSSSDNIAL	HLA‐DQA10101‐DQB10603						

*Note*: Rows refer to individual 15‐mer peptides derived from the SARS‐CoV‐2 Spike (S), Nucleocapsid (N), or Membrane (M) protein (1st column), as well as their ancestor peptide pools (2nd column) and the specific peptide sequence (3rd column). Likewise, the likely HLA restriction of the peptides, that were predicted by NetMHCpan given the HLA allotypes by reactive donors, are listed (4th column).

**TABLE 4:** Validation of core peptides’ immunogenicity.

					PBMC						Expanded cells					
Core peptide ID	Ancestor 15mer peptide	Ancestor peptide pool	HLA‐restric‐tion	Core peptide sequence	#Tests total	#CD4+/ CD8+ res‐ponse	#CD4+ res‐ponse	#CD8+ res‐ponse	#Total postive hits	Total postive hits [%]	#Tests total	#CD4+/ CD8+ res‐ponse	#CD4+ res‐ponse	#CD8+ res‐ponse	#Total postive hits	Total postive hits [%]
DRB1_0101_P1	CoV_Prot_S_821‐835	CoV_Prot_S_802‐852	DRB1_0101	VTLADAGFI	1	0	0	1	1	1/1 (100)	1	0	0	0	0	0/1
DRB1_0101_P2	CoV_Prot_S_829‐843	CoV_Prot_S_802‐852	DRB1_0101	FIKQYGDCL	1	0	0	1	1	1/1 (100)	1	0	0	0	0	0/1
DRB1_0101_P3	CoV_Prot_S_896‐910	CoV_Prot_S_881‐927	DRB1_0101	FAMQMAYRF	1	0	0	1	1	1/1 (100)	1	0	0	0	0	0/1
DRB1_0101_P4	CoV_Prot_S_813‐827	CoV_Prot_S_802‐852	DRB1_0101	IEDLLFNKV	1	0	0	1	1	1/1 (100)	1	0	1	0	1	1/1 (100)
DRB1_0301_0401_P1	CoV_Prot_S_445‐459	CoV_Prot_S_441‐475	DRB1_0301	YNYLYRLFR	1	0	1	0	1	1/1 (100)	2	0	0	0	0	0/2
DRB1_0301_0401_P1	CoV_Prot_S_445‐459	CoV_Prot_S_441‐475	DRB1_0401	YNYLYRLFR	1	0	0	0	0	0/1	4	0	0	0	0	0/4
DRB1_0301_P1	CoV_Prot_S_457‐471	CoV_Prot_S_441‐475	DRB1_0301	LKPFERDIS	1	0	0	0	0	0/1	2	0	0	0	0	0/2
DRB1_0301_P2	CoV_Prot_S_765‐779	CoV_Prot_S_737‐779	DRB1_0301	LTGIAVEQD	2	0	0	0	0	0/2	3	0	1	0	1	1/3 (33,33)
DRB1_0401_0701_P1	CoV_Prot_M_201‐215	CoV_Prot_M_173‐222	DRB1_0401	YKLNTDHSS	1	0	0	0	0	0/1	3	0	0	1	1	1/3 (33)
DRB1_0401_0701_P1	CoV_Prot_M_201‐215	CoV_Prot_M_173‐222	DRB1_0701	YKLNTDHSS	1	0	0	0	0	0/1	3	0	0	1	1	1/3 (33)
DRB1_0401_1501_P1	CoV_Prot_S_799‐813	CoV_Prot_S_769‐813	DRB1_0401	ILPDPSKPS	1	0	0	0	0	0/1	3	0	0	2	2	2/3 (66,67)
DRB1_0401_1501_P1	CoV_Prot_S_799‐813	CoV_Prot_S_769‐813	DRB1_1501	ILPDPSKPS	5	0	0	1	1	1/5 (20)	6	0	0	0	0	0/6
DRB1_0401_HLA‐A02:01_P1	CoV_Prot_S_817‐831	CoV_Prot_S_802‐852	DRB1_0401	LLFNKVTLA	1	0	0	0	0	0/1	3	0	0	1	1	1/3 (33)
DRB1_0401_HLA‐A02:01_P1	CoV_Prot_S_817‐831	CoV_Prot_S_802‐852	HLA‐A02:01	LLFNKVTLA	9	0	0	0	0	0/9	8	0	1	1	2	2/8 (25)
DRB1_0401_P1	CoV_Prot_S_813‐827	CoV_Prot_S_802‐852	DRB1_0401	FIEDLLFNK	1	0	0	0	0	0/1	3	0	0	1	1	1/3 (33)
DRB1_0701_P2	CoV_Prot_M_173‐187	CoV_Prot_M_173‐222	DRB1_0701	YKLGASQRV	1	0	0	0	0	0/1	2	0	0	1	1	1/2 (50)
DRB1_1101_HLA‐A02:01_P1	CoV_Prot_S_265‐279	CoV_Prot_S_245‐295	DRB1_1101	YLQPRTFLL	3	0	0	0	0	0/3	3	0	0	2	2	2/3 (67)
DRB1_1101_HLA‐A02:01_P1	CoV_Prot_S_265‐279	CoV_Prot_S_245‐295	HLA‐A02:01	YLQPRTFLL	10	0	0	1	1	1/10 (10)	10	0	0	8	8	8/10 (80)
DRB1_1101_P1	CoV_Prot_M_009‐023	CoV_Prot_M_1‐47	DRB1_1101	VEELKKLLE	2	0	1	0	1	1/2 (50)	2	0	0	0	0	0/2
DRB1_1101_P2	CoV_Prot_S_269‐283	CoV_Prot_S_245‐295	DRB1_1101	FLLKYNENG	3	0	0	0	0	0/3	3	0	0	0	0	0/3
DRB1_1101_P3	CoV_Prot_S_150‐164	CoV_Prot_S_121‐175	DRB1_1101	MESEFRVYS	2	0	0	0	0	0/2	2	0	0	1	1	1/2 (50)
DRB1_1101_P4	CoV_Prot_S_349‐363	CoV_Prot_S_342‐390	DRB1_1101	YAWNRKRIS	2	0	0	0	0	0/2	1	1	0	0	1	1/1 (100)
DRB1_1101_P6	CoV_Prot_S_253‐267	CoV_Prot_S_245‐295	DRB1_1101	WTAGAAAYY	3	0	0	0	0	0/3	3	0	0	0	0	0/3
DRB1_1301_HLA‐C14:02_P1	CoV_Prot_S_781‐795	CoV_Prot_S_769‐813	DRB1_1301	VFAQVKQIY	1	1	0	0	1	1/1 (100)	1	0	0	0	0	0/1
DRB1_1301_HLA‐C14:02_P1	CoV_Prot_S_781‐795	CoV_Prot_S_769‐813	HLA‐C14:02	VFAQVKQIY	2	1	0	0	1	1/2 (50)	2	0	0	0	0	0/2
DRB1_1501_HLA‐C03:03_P1	CoV_Prot_S_202‐216	CoV_Prot_S_165‐216	DRB1_1501	YSKHTPINL	1	0	0	1	1	1/1 (100)	1	0	0	0	0	0/1
DRB1_1501_HLA‐C03:03_P1	CoV_Prot_S_198‐212	CoV_Prot_S_165‐216	HLA‐C03:03	YSKHTPINL	2	0	0	0	0	0/2	1	0	0	0	0	0/1
DRB1_1501_P1	CoV_Prot_M_173‐187	CoV_Prot_M_173‐222	DRB1_1501	LSYYKLGAS	5	2	0	0	2	2/5 (40)	6	1	1	0	2	2/6 (33)
DRB1_1501_P10	CoV_Prot_S_194‐208	CoV_Prot_S_165‐216	DRB1_1501	IDGYFKIYS	5	0	0	1	1	1/5 (20)	6	0	0	0	0	0/6
DRB1_1501_P11	CoV_Prot_S_353‐367	CoV_Prot_S_342‐390	DRB1_1501	ISNCVADYS	5	0	1	1	2	2/5 (40)	5	0	0	0	0	0/5
DRB1_1501_P12	CoV_Prot_S_198‐212	CoV_Prot_S_165‐216	DRB1_1501	FKIYSKHTP	5	0	0	1	1	1/5 (20)	6	0	0	0	0	0/6
DRB1_1501_P13	CoV_Prot_S_345‐359	CoV_Prot_S_342‐390	DRB1_1501	VYAWNRKRI	5	0	1	1	2	2/5 (40)	6	0	1	0	1	1/6 (17)
DRB1_1501_P14	CoV_Prot_S_753‐765	CoV_Prot_S_737‐779	DRB1_1501	YGSFCTQLN	5	0	0	1	1	1/5 (20)	6	1	0	0	1	1/6 (17)
DRB1_1501_P15	CoV_Prot_S_990‐1004	CoV_Prot_S_981‐1023	DRB1_1501	IDRLITGRL	5	0	0	0	0	0/5	6	1	0	0	1	1/6 (17)
DRB1_1501_P16	CoV_Prot_S_997‐1011	CoV_Prot_S_981‐1023	DRB1_1501	LQSLQTYVT	5	1	0	1	2	2/5 (40)	6	0	0	1	1	1/6 (17)
DRB1_1501_P17	CoV_Prot_S_1056‐1070	CoV_Prot_S_1051‐1099	DRB1_1501	VVFLHVTYV	5	0	0	0	0	0/5	6	0	0	1	1	1/6 (17)
DRB1_1501_P18	CoV_Prot_S_342‐356	CoV_Prot_S_342‐390	DRB1_1501	FASVYAWNR	5	0	0	0	0	0/5	5	0	1	0	1	2/6 (33)
DRB1_1501_P19	CoV_Prot_N_49‐61	CoV_Prot_N_40‐87	DRB1_1501	WFTALTQHG	5	0	0	0	0	0/5	6	0	1	0	1	1/6 (16,67)
DRB1_1501_P2	CoV_Prot_M_197‐211	CoV_Prot_M_173‐222	DRB1_1501	IGNYKLNTD	5	1	3	0	4	4/5 (80)	5	1	0	1	2	2/5 (40)
DRB1_1501_P3	CoV_Prot_N_037‐051	CoV_Prot_N_1‐51	DRB1_1501	PQGLPNNTA	5	0	0	0	0	0/5	6	0	1	1	2	2/6 (33)
DRB1_1501_P4	CoV_Prot_S_001‐014	CoV_Prot_S_1‐51	DRB1_1501	LVLLPLVSS	5	0	1	1	2	2/5 (40)	6	0	0	0	0	0/6
DRB1_1501_P5	CoV_Prot_S_369‐383	CoV_Prot_S_342‐390	DRB1_1501	FSTFKCYGV	5	0	0	0	0	0/5	6	0	0	0	0	0/6
DRB1_1501_P6	CoV_Prot_S_376‐390	CoV_Prot_S_342‐390	DRB1_1501	FKCYGVSPT	5	0	0	0	0	0/5	5	0	0	0	0	0/5
DRB1_1501_P7	CoV_Prot_S_072‐086	CoV_Prot_S_41‐91	DRB1_1501	TKRFDNPVL	5	0	0	0	0	0/5	6	0	0	0	0	0/6
DRB1_1501_P8	CoV_Prot_S_138‐152	CoV_Prot_S_121‐175	DRB1_1501	VYYHKNNKS	5	0	0	0	0	0/5	6	0	0	0	0	0/6
DRB1_1501_P9	CoV_Prot_S_189‐203	CoV_Prot_S_165‐216	DRB1_1501	FVFKNIDGY	5	0	0	0	0	0/5	6	0	0	0	0	0/6
HLA‐A01:01_P1	CoV_Prot_M_208‐222	CoV_Prot_M_173‐222	HLA‐A01:01	SSDNIALLV	7	0	0	0	0	0/7	7	0	1	1	2	2/7 (29)
HLA‐A01:01_P2	CoV_Prot_N_405‐419	CoV_Prot_N_377‐419	HLA‐A01:01	MSSADSTQA	12	7	2	0	9	9/12 (75)	7	0	1	2	3	3/7 (43)
HLA‐A01:01_P3	CoV_Prot_S_157‐171	CoV_Prot_S_121‐175	HLA‐A01:01	SANNCTFEY	4	1	0	1	2	2/4 (50)	5	0	0	1	1	1/5 (20)
HLA‐A01:01_P4	CoV_Prot_M_165‐179	CoV_Prot_M_135‐183	HLA‐A01:01	ATSRTLSYY	6	0	1	0	1	1/6 (16,67)	6	0	2	0	2	2/6 (33,33)
HLA‐A02:01_HLAC04:01_P1	CoV_Prot_S_105‐119	CoV_Prot_S_81‐130	HLA‐A02:01	TLDSKTQSL	10	0	0	0	0	0/10	10	1	0	3	4	4/10 (40)
HLA‐A02:01_HLAC04:01_P1	CoV_Prot_S_105‐119	CoV_Prot_S_81‐130	HLA‐C04:01	TLDSKTQSL	1	0	0	1	1	1/1 (100)	1	0	0	0	0	0/1
HLA‐A02:01_P1	CoV_Prot_M_012‐026	CoV_Prot_M_1‐47	HLA‐A02:01	KLLEQWNLV	6	0	0	0	0	0/6	6	0	0	1	1	1/6 (17)
HLA‐A02:01_P10	CoV_Prot_S_972‐984	CoV_Prot_S_955‐991	HLA‐A02:01	VLNDILSRL	10	0	0	0	0	0/10	10	0	0	3	3	3/10 (30)
HLA‐A02:01_P11	CoV_Prot_S_981‐995	CoV_Prot_S_981‐1023	HLA‐A02:01	RLDKVEAEV	5	0	0	0	0	0/5	4	1	0	1	2	2/4 (50)
HLA‐A02:01_P12	CoV_Prot_S_1045‐1059	CoV_Prot_S_1012‐1059	HLA‐A02:01	HLMSFPQSA	10	0	0	1	1	1/10 (10)	10	0	0	1	1	1/10 (10)
HLA‐A02:01_P13	CoV_Prot_S_1209‐1223	CoV_Prot_S_1195‐1230	HLA‐A02:01	IKWPWYIWL	9	0	0	0	0	0/9	9	0	0	1	1	1/9 (11,11)
HLA‐A02:01_P14	CoV_Prot_S_245‐259	CoV_Prot_S_245‐295	HLA‐A02:01	YLTPGDSSS	9	0	0	0	0	0/9	9	0	1	1	2	2/9 (22,22)
HLA‐A02:01_P2	CoV_Prot_M_060‐070	CoV_Prot_M_37‐73	HLA‐A02:01	TLACFVLAA	5	0	1	0	1	1/5 (20)	6	0	0	2	2	2/6 (33)
HLA‐A02:01_P3	CoV_Prot_S_685‐699	CoV_Prot_S_665‐707	HLA‐A02:01	SIIAYTMSL	5	0	1	0	1	1/5 (20)	5	0	0	2	2	2/5 (40)
HLA‐A02:01_P6	CoV_Prot_M_65‐76	CoV_Prot_M_65‐105	HLA‐A02:01	FVLAAVYRI	6	0	0	0	0	0/6	6	0	0	0	0	0/6
HLA‐A02:01_P7	CoV_Prot_N_217‐230	CoV_Prot_N_194‐234	HLA‐A02:01	LLLDRLNQL	6	0	0	0	0	0/6	7	0	1	0	1	1/7 (14,29)
HLA‐A02:01_P8	CoV_Prot_S_417‐431	CoV_Prot_S_381‐431	HLA‐A02:01	KIADYNYKL	10	0	0	0	0	0/10	9	0	0	0	0	0/9
HLA‐A02:01_P9	CoV_Prot_S_915‐927	CoV_Prot_S_881‐927	HLA‐A02:01	VLYENQKLI	5	1	1	0	2	2/5 (40)	5	0	0	2	2	2/5 (40)
HLA‐A03:01_HLA‐A11:01_P1	CoV_Prot_S_1061‐1075	CoV_Prot_S_1051‐1099	HLA‐A03:01	VTYVPAQEK	6	0	0	0	0	0/6	8	0	0	5	5	5/8 (62,5)
HLA‐A03:01_HLA‐A11:01_P1	CoV_Prot_S_1061‐1075	CoV_Prot_S_1051‐1099	HLA‐A11:01	VTYVPAQEK	1	0	0	0	0	0/1	1	0	0	1	1	1/1 (100)
HLA‐A03:01_P1	CoV_Prot_M_149‐163	CoV_Prot_M_135‐183	HLA‐A03:01	RIAGHHLGR	6	1	0	0	1	1/6 (17)	7	1	0	1	2	2/7 (29)
HLA‐A03:01_P2	CoV_Prot_S_345‐359	CoV_Prot_S_342‐390	HLA‐A03:01	SVYAWNRKR	8	0	0	1	1	1/8 (13)	11	2	0	2	4	4/11 (36)
HLA‐A03:01_P3	CoV_Prot_S_376‐390	CoV_Prot_S_342‐390	HLA‐A03:01	KCYGVSPTK	6	0	0	4	4	4/6 (66,67)	8	1	0	7	8	8/8 (100)
HLA‐A03:01_P4	CoV_Prot_S_526‐539	CoV_Prot_S_521‐567	HLA‐A03:01	KSTNLVKNK	7	0	0	1	1	1/7 (14,29)	10	0	0	2	2	2/10 (20)
HLA‐A03:01_P5	CoV_Prot_S_41‐55	CoV_Prot_S_41‐91	HLA‐A03:01	KVFRSSVLH	6	0	0	0	0	0/6	7	0	0	2	2	2/7 (28,57)
HLA‐A03:01_P6	CoV_Prot_S_138‐152	CoV_Prot_S_121‐175	HLA‐A03:01	GVYYHKNNK	8	1	0	0	1	1/8 (12,5)	10	1	0	0	1	1/10 (10)
HLA‐A11:01_P1	CoV_Prot_S_669‐683	CoV_Prot_S_665‐707	HLA‐A11:01	QTQTNSPRR	1	0	1	0	1	1/1 (100)	1	0	0	0	0	0/1
HLA‐A11:01_P2	CoV_Prot_S_719‐733	CoV_Prot_S_698‐747	HLA‐A11:01	EILPVSMTK	1	0	0	0	0	0/1	1	0	0	1	1	1/1 (100)
HLA‐A11:01_P3	CoV_Prot_S_753‐765	CoV_Prot_S_737‐779	HLA‐A11:01	GSFCTQLNR	1	0	0	0	0	0/1	1	0	0	1	1	1/1 (100)
HLA‐A11:01_P4	CoV_Prot_S_546‐560	CoV_Prot_S_521‐567	HLA‐A11:01	GVLTESNKK	2	0	0	0	0	0/2	1	0	0	0	0	0/1
HLA‐A11:01_P5	CoV_Prot_S_781‐795	CoV_Prot_S_769‐813	HLA‐A11:01	QIYKTPPIK	1	0	0	0	0	0/1	1	0	0	1	1	1/1 (100)
HLA‐A11:01_P6	CoV_Prot_S_1093‐1107	CoV_Prot_S_1089‐1135	HLA‐A11:01	GTHWFVTQR	1	0	0	0	0	0/1	1	0	0	0	0	0/1
HLA‐A11:01_P7	CoV_Prot_S_364‐378	CoV_Prot_S_342‐390	HLA‐A11:01	NSASFSTFK	1	0	0	0	0	0/1	1	0	0	0	0	0/1
HLA‐A23:01_P1	CoV_Prot_M_042‐056	CoV_Prot_M_37‐73	HLA‐A23:01	LYIIKLIFL	1	0	0	0	0	0/1	1	0	0	0	0	0/1
HLA‐A23:01_P2	CoV_Prot_M_89‐103	CoV_Prot_M_65‐105	HLA‐A23:01	YFIASFRLF	1	0	0	0	0	0/1	1	0	0	0	0	0/1
HLA‐A25:01_P1	CoV_Prot_M_12‐26	CoV_Prot_M_1‐47	HLA‐A25:01	ELKKLLEQW	1	0	0	0	0	0/1	2	0	0	0	0	0/2
HLA‐A26:01_P1	CoV_Prot_S_044‐058	CoV_Prot_S_41‐91	HLA‐A26:01	STQDLFLPF	1	0	0	0	0	0/1	1	0	0	0	0	0/1
HLA‐A29:02_HLA‐C02:02_P1	CoV_Prot_S_024‐038	CoV_Prot_S_1‐51	HLA‐A29:02	NSFTRGVYY	1	0	0	0	0	0/1	1	0	0	0	0	0/1
HLA‐A29:02_HLA‐C02:02_P1	CoV_Prot_S_024‐038	CoV_Prot_S_1‐51	HLA‐C02:02	NSFTRGVYY	1	0	0	0	0	0/1	2	0	0	0	0	0/2
HLA‐A68:01_P1	CoV_Prot_M_137‐151	CoV_Prot_M_135‐183	HLA‐A68:01	LVIGAVILR	2	0	0	0	0	0/2	1	0	0	0	0	0/1
HLA‐A68:01_P2	CoV_Prot_S_673‐687	CoV_Prot_S_665‐707	HLA‐A68:01	QTNSPRRAR	2	0	0	0	0	0/2	2	0	0	0	0	0/2
HLA‐B07:02_P1	CoV_Prot_N_102‐116	CoV_Prot_N_78‐127	HLA‐B07:02	SPRWYFYYL	5	0	0	0	0	0/5	6	1	1	2	4	4/6 (67)
HLA‐B07:02_P2	CoV_Prot_N_37‐51	CoV_Prot_N_1‐51	HLA‐B07:02	RPQGLPNNT	6	0	0	0	0	0/6	6	0	0	0	0	0/6
HLA‐B07:02_P3	CoV_Prot_N_253‐267	CoV_Prot_N_224‐274	HLA‐B07:02	KPRQKRTAT	6	0	0	0	0	0/6	7	0	1	1	2	2/7 (28,57)
HLA‐B07:02_P4	CoV_Prot_S_678‐692	CoV_Prot_S_665‐707	HLA‐B07:02	SPRRARSVA	8	0	0	1	1	1/8 (12,5)	7	0	0	4	4	4/7 (57,14)
HLA‐B07:02_P5	CoV_Prot_S_202‐216	CoV_Prot_S_165‐216	HLA‐B07:02	TPINLVRDL	7	0	0	0	0	0/7	8	0	0	0	0	0/8
HLA‐B08:01_P1	CoV_Prot_M_144‐158	CoV_Prot_M_135‐183	HLA‐B08:01	HLRIAGHHL	4	0	0	0	0	0/4	5	0	1	0	1	1/5 (20)
HLA‐B08:01_P2	CoV_Prot_S_869‐883	CoV_Prot_S_841‐891	HLA‐B08:01	MIAQYTSAL	4	0	0	0	0	0/4	6	0	1	1	2	2/6 (33,33)
HLA‐B14:01_P1	CoV_Prot_S_37‐51	CoV_Prot_S_1‐51	HLA‐B14:01	DKVFRSSVL	1	0	0	0	0	0/1	1	0	0	0	0	0/1
HLA‐B14:01_P2	CoV_Prot_S_41‐55	CoV_Prot_S_41‐91	HLA‐B14:01	FRSSVLHST	1	0	0	0	0	0/1	1	0	0	0	0	0/1
HLA‐B15:01_P1	CoV_Prot_S_494‐508	CoV_Prot_S_461‐508	HLA‐B15:01	FQPTNGVGY	2	0	0	0	0	0/2	2	0	0	2	2	2/2 (100)
HLA‐B15:01_P2	CoV_Prot_S_799‐813	CoV_Prot_S_769‐813	HLA‐B15:01	SQILPDPSK	1	0	1	0	1	1/1 (100)	1	0	0	1	1	1/1 (100)
HLA‐B15:01_P3	CoV_Prot_S_178‐192	CoV_Prot_S_165‐216	HLA‐B15:01	GNFKNLREF	2	0	0	0	0	0/2	2	0	0	0	0	0/2
HLA‐B27:05_HLA‐C07:02_P1	CoV_Prot_S_994‐1008	CoV_Prot_S_981‐1023	HLA‐B27:05	GRLQSLQTY	1	0	0	0	0	0/1	1	0	0	1	1	1/1 (100)
HLA‐B27:05_HLA‐C07:02_P1	CoV_Prot_S_994‐1008	CoV_Prot_S_981‐1023	HLA‐C07:02	GRLQSLQTY	7	0	0	1	1	1/7 (14)	9	0	0	2	2	2/9 (22)
HLA‐B27:05_P1	CoV_Prot_S_077‐091	CoV_Prot_S_41‐91	HLA‐B27:05	KRFDNPVLP	1	1	0	0	1	1/1 (100)	1	0	0	0	0	0/1
HLA‐B27:05_P2	CoV_Prot_S_342‐356	CoV_Prot_S_342‐390	HLA‐B27:05	TRFASVYAW	1	0	0	0	0	0/1	1	0	0	0	0	0/1
HLA‐B35:03_P1	CoV_Prot_S_081‐095	CoV_Prot_S_81‐130	HLA‐B35:03	LPFNDGVYF	1	0	0	1	1	1/1 (100)	1	0	0	0	0	0/1
HLA‐B35:03_P2	CoV_Prot_S_1257‐1271	CoV_Prot_S_1222‐1273	HLA‐B35:03	EPVLKGVKL	1	0	0	0	0	0/1	1	0	0	1	1	1/1 (100)
HLA‐B35:03_P3	CoV_Prot_S_18‐32	CoV_Prot_S_1‐51	HLA‐B35:03	LPPAYTNSF	1	0	0	0	0	0/1	1	0	0	0	0	0/1
HLA‐B38:01_P1	CoV_Prot_S_769‐783	CoV_Prot_S_769‐813	HLA‐B38:01	EQDKNTQEV	1	0	0	0	0	0/1	1	0	1	0	1	1/1 (100)
HLA‐B40:01_P1	CoV_Prot_S_1149‐1163	CoV_Prot_S_1123‐1166	HLA‐B40:01	KYFKNHTSP	1	0	0	0	0	0/1	2	0	0	0	0	0/2
HLA‐B40:01_P2	CoV_Prot_S_1175‐1189	CoV_Prot_S_1157‐1205	HLA‐B40:01	KEIDRLNEV	4	0	0	0	0	0/4	3	0	0	0	0	0/3
HLA‐B44:02_P1	CoV_Prot_S_1203‐1215	CoV_Prot_S_1195‐1230	HLA‐B44:02	YEQYIKWPW	2	0	0	0	0	0/2	2	0	0	0	0	0/2
HLA‐B44:02_P2	CoV_Prot_S_825‐839	CoV_Prot_S_802‐852	HLA‐B44:02	ADAGFIKQY	2	0	0	0	0	0/2	2	0	0	1	1	1/2 (50)
HLA‐B44:02_P3	CoV_Prot_S_1197‐1211	CoV_Prot_S_1195‐1230	HLA‐B44:02	QELGKYEQY	2	0	0	0	0	0/2	2	0	0	0	0	0/2
HLA‐B44:03_P1	CoV_Prot_N_321‐335	CoV_Prot_N_305‐347	HLA‐B44:03	MEVTPSGTW	1	0	0	0	0	0/1	1	0	0	0	0	0/1
HLA‐B51:01_P1	CoV_Prot_S_005‐019	CoV_Prot_S_1‐51	HLA‐B51:01	LPLVSSQCV	4	0	1	0	1	1/4 (25)	5	0	0	0	0	0/5
HLA‐B51:01_P2	CoV_Prot_S_709‐723	CoV_Prot_S_698‐747	HLA‐B51:01	IPTNFTISV	5	0	0	0	0	0/5	5	0	0	1	1	1/5 (20)
HLA‐C02:02_DRB1_0701_P1	CoV_Prot_M_165‐179	CoV_Prot_M_135‐183	HLA‐C02:02	VATSRTLSY	1	0	0	0	0	0/1	2	1	0	0	1	1/2 (50)
HLA‐C02:02_DRB1_0701_P1	CoV_Prot_M_177‐191	CoV_Prot_M_173‐222	DRB1_0701	VATSRTLSY	1	0	0	0	0	0/1	3	0	0	0	0	0/3
HLA‐C02:02_HLA‐C12:02_P1	CoV_Prot_S_683‐696	CoV_Prot_S_665‐707	HLA‐C02:02	VASQSIIAY	1	0	0	0	0	0/1	2	0	0	0	0	0/2
HLA‐C02:02_HLA‐C12:02_P1	CoV_Prot_S_683‐696	CoV_Prot_S_665‐707	HLA‐C12:02	VASQSIIAY	1	0	0	0	0	0/1	2	0	0	0	0	0/2
HLA‐C02:02_P1	CoV_Prot_M_193‐207	CoV_Prot_M_173‐222	HLA‐C02:02	YSRYRIGNY	1	0	0	0	0	0/1	2	0	0	0	0	0/2
HLA‐C02:02_P3	CoV_Prot_S_170‐184	CoV_Prot_S_165‐216	HLA‐C02:02	VSQPFLMDL	1	0	0	0	0	0/1	2	0	0	0	0	0/2
HLA‐C02:02_P4	CoV_Prot_S_304‐319	CoV_Prot_S_285‐327	HLA‐C02:02	KGIYQTSNF	1	0	0	0	0	0/1	2	0	0	0	0	0/2
HLA‐C02:02_P5	CoV_Prot_S_369‐383	CoV_Prot_S_342‐390	HLA‐C02:02	ASFSTFKCY	1	0	0	0	0	0/1	2	0	0	1	1	1/2 (50)
HLA‐C02:02_P7	CoV_Prot_M_34‐47	CoV_Prot_M_1‐47	HLA‐C02:02	FAYANRNRF	1	0	0	0	0	0/1	2	1	0	1	2	2/2 (100)
HLA‐C02:02_P8	CoV_Prot_M_27‐37	CoV_Prot_M_1‐47	HLA‐C02:02	LTWICLLQF	1	0	0	0	0	0/1	2	0	1	1	2	2/2 (100)
HLA‐C02:02_P9	CoV_Prot_S_1259‐1273	CoV_Prot_S_1222‐1273	HLA‐C02:02	VLKGVKLHY	1	0	0	0	0	0/1	2	0	0	0	0	0/2
HLA‐C03:04_P1	CoV_Prot_S_881‐895	CoV_Prot_S_881‐927	HLA‐C03:04	WTFGAGAAL	2	0	1	0	1	1/2 (50)	3	0	0	1	1	1/3 (33)
HLA‐C03:04_P2	CoV_Prot_S_865‐879	CoV_Prot_S_841‐891	HLA‐C03:04	IAQYTSALL	5	0	0	0	0	0/5	5	0	1	0	1	1/5 (20)
HLA‐C04:01_DRB1_1501_P1	CoV_Prot_S_165‐179	CoV_Prot_S_165‐216	HLA‐C04:01	TFEYVSQPF	4	0	0	0	0	0/4	4	0	0	0	0	0/4
HLA‐C04:01_DRB1_1501_P1	CoV_Prot_S_161‐175	CoV_Prot_S_121‐175	DRB1_1501	TFEYVSQPF	5	0	0	1	1	1/5 (20)	6	0	0	0	0	0/6
HLA‐C04:01_DRB1_1501_P1	CoV_Prot_S_161‐175	CoV_Prot_S_121‐175	HLA‐C04:01	TFEYVSQPF	1	0	0	1	1	1/1 (100)	1	0	0	1	1	1/1 (100)
HLA‐C04:01_HLA‐C07:02_P1	CoV_Prot_S_72‐86	CoV_Prot_S_41‐91	HLA‐C04:01	RFDNPVLPF	4	0	0	1	1	1/4 (25)	3	0	1	0	1	1/3 (33,33)
HLA‐C04:01_HLA‐C07:02_P1	CoV_Prot_S_72‐86	CoV_Prot_S_41‐91	HLA‐C07:02	RFDNPVLPF	6	2	0	0	2	2/6 (33,33)	6	0	0	1	1	1/6 (16,67)
HLA‐C04:01_P1	CoV_Prot_M_105‐119	CoV_Prot_M_97‐147	HLA‐C04:01	SFNPETNIL	2	0	0	0	0	0/2	2	0	0	1	1	1/2 (50)
HLA‐C04:01_P2	CoV_Prot_N_78‐92	CoV_Prot_N_78‐127	HLA‐C04:01	SPDDQIGYY	2	0	0	1	1	1/2 (50)	2	0	0	0	0	0/2
HLA‐C04:01_P3	CoV_Prot_N_333‐347	CoV_Prot_N_305‐347	HLA‐C04:01	KLDDKDPNF	2	0	0	0	0	0/2	2	1	0	0	1	1/2 (50)
HLA‐C04:01_P6	CoV_Prot_S_1136‐1149	CoV_Prot_S_1123‐1166	HLA‐C04:01	VYDPLQPEL	4	0	0	0	0	0/4	4	0	0	1	1	1/4 (25)
HLA‐C05:01_P1	CoV_Prot_S_285‐299	CoV_Prot_S_285‐327	HLA‐C05:01	ITDAVDCAL	2	0	0	0	0	0/2	2	0	0	0	0	0/2
HLA‐C05:01_P2	CoV_Prot_S_581‐595	CoV_Prot_S_557‐607	HLA‐C05:01	ILDITPCSF	2	0	0	0	0	0/2	2	0	0	0	0	0/2
HLA‐C05:01_P3	CoV_Prot_S_713‐727	CoV_Prot_S_698‐747	HLA‐C05:01	FTISVTTEI	2	0	0	0	0	0/2	2	0	0	0	0	0/2
HLA‐C05:01_P4	CoV_Prot_S_745‐759	CoV_Prot_S_737‐779	HLA‐C05:01	STECSNLLL	2	0	0	0	0	0/2	2	0	0	0	0	0/2
HLA‐C05:01_P5	CoV_Prot_S_841‐855	CoV_Prot_S_841‐891	HLA‐C05:01	LGDIAARDL	2	0	0	0	0	0/2	2	0	0	1	1	1/2 (50)
HLA‐C05:01_P6	CoV_Prot_S_1117‐1131	CoV_Prot_S_1089‐1135	HLA‐C05:01	FVSGNCDVV	2	0	0	0	0	0/2	2	0	0	0	0	0/2
HLA‐C05:01_P7	CoV_Prot_S_289‐303	CoV_Prot_S_285‐327	HLA‐C05:01	ALDPLSETK	2	0	0	0	0	0/2	2	0	1	1	2	2/2 (100)
HLA‐C07:01_P1	CoV_Prot_S_553‐567	CoV_Prot_S_521‐567	HLA‐C07:01	KKFLPFQQF	4	0	0	1	1	1/4 (25)	4	0	0	1	1	1/4 (25)
HLA‐C07:01_P2	CoV_Prot_M_102‐116	CoV_Prot_M_97‐147	HLA‐C07:01	ARTRSMWSF	4	0	0	0	0	0/4	6	0	0	0	0	0/6
HLA‐C07:01_P3	CoV_Prot_S_785‐799	CoV_Prot_S_769‐813	HLA‐C07:01	YKTPPIKDF	5	0	0	1	1	1/5 (20)	6	0	0	0	0	0/6
HLA‐C07:02_P11	CoV_Prot_S_322‐335	CoV_Prot_S_313‐351	HLA‐C07:02	VRFPNITNL	8	1	1	0	2	2/8 (25)	9	0	1	0	1	1/9 (11,11)
HLA‐C07:02_P2	CoV_Prot_M_169‐183	CoV_Prot_M_135‐183	HLA‐C07:02	SRTLSYYKL	7	1	0	0	1	1/7 (14,29)	7	0	1	1	2	2/7 (28,57)
HLA‐C07:02_P3	CoV_Prot_N_158‐172	CoV_Prot_N_158‐203	HLA‐C07:02	LQLPQGTTL	7	0	1	1	2	2/7 (28,57)	7	0	1	0	1	1/7 (14,29)
HLA‐C07:02_P4	CoV_Prot_S_438‐452	CoV_Prot_S_421‐455	HLA‐C07:02	SKVGGNYNY	8	0	0	2	2	2/8 (25)	9	0	0	1	1	1/9 (11,11)
HLA‐C07:02_P5	CoV_Prot_S_486‐500	CoV_Prot_S_461‐508	HLA‐C07:02	YFPLQSYGF	8	0	0	1	1	1/8 (12,5)	7	0	1	2	3	3/7 (42,86)
HLA‐C07:02_P6	CoV_Prot_S_573‐587	CoV_Prot_S_557‐607	HLA‐C07:02	VRDPQTLEI	8	0	0	2	2	2/8 (25)	9	0	1	0	1	1/9 (11,11)
HLA‐C07:02_P7	CoV_Prot_S_165‐179	CoV_Prot_S_165‐216	HLA‐C07:02	EYVSQPFLM	8	1	0	0	1	1/8 (12,5)	9	0	0	2	2	2/9 (22,22)
HLA‐C07:02_P8	CoV_Prot_S_18‐32	CoV_Prot_S_1‐51	HLA‐C07:02	TRTQLPPAY	7	0	0	1	1	1/7 (14,29)	9	0	1	0	1	1/9 (11,11)
HLA‐C08:02_P1	CoV_Prot_S_865‐879	CoV_Prot_S_841‐891	HLA‐C08:02	LTDEMIAQY	1	0	0	0	0	0/1	1	0	0	1	1	1/1 (100)
HLA‐C12:02_P1	CoV_Prot_S_1189‐1203	CoV_Prot_S_1157‐1205	HLA‐C12:02	VAKNLNESL	1	0	0	0	0	0/1	2	0	0	0	0	0/2
HLA‐C12:03_HLA‐B51:01_P1	CoV_Prot_S_117‐130	CoV_Prot_S_81‐130	HLA‐B51:01	NATNVVIKV	5	0	0	0	0	0/5	5	0	0	3	3	3/5 (60)
HLA‐C12:03_HLA‐B51:01_P1	CoV_Prot_S_121‐135	CoV_Prot_S_121‐175	HLA‐C12:03	NATNVVIKV	1	0	0	0	0	0/1	2	0	0	0	0	0/2
HLA‐C12:03_HLA‐B51:01_P1	CoV_Prot_S_121‐135	CoV_Prot_S_121‐175	HLA‐B51:01	NATNVVIKV	4	0	1	0	1	1/4 (25)	5	0	0	3	3	3/5 (60)
HLA‐C12:03_P2	CoV_Prot_S_249‐263	CoV_Prot_S_245‐295	HLA‐C12:03	SSSGWTAGA	2	0	0	0	0	0/2	4	1	0	0	1	1/4 (25)
HLA‐C14:02_P1	CoV_Prot_M_193‐207	CoV_Prot_M_173‐222	HLA‐C14:02	RYRIGNYKL	2	0	0	0	0	0/2	2	0	0	0	0	0/2
HLA‐C14:02_P2	CoV_Prot_S_698‐712	CoV_Prot_S_698‐747	HLA‐C14:02	LGAENSVAY	2	0	0	0	0	0/2	2	0	0	1	1	1/2 (50)
HLA‐C15:02_P1	CoV_Prot_S_024‐038	CoV_Prot_S_1‐51	HLA‐C15:02	YTNSFTRGV	1	0	0	0	0	0/1	1	0	0	0	0	0/1
HLA‐C15:02_P2	CoV_Prot_S_057‐071	CoV_Prot_S_41‐91	HLA‐C15:02	VTWFHAIHV	2	0	0	0	0	0/2	2	0	0	2	2	2/2 (100)
HLA‐C15:02_P3	CoV_Prot_S_93‐107	CoV_Prot_S_81‐130	HLA‐C15:02	ASTEKSNII	3	0	0	0	0	0/3	3	1	0	1	2	2/3 (66,67)
HLA‐C16:01_DRB1_1501_P1	CoV_Prot_S_154‐168	CoV_Prot_S_121‐175	HLA‐C16:01	YSSANNCTF	1	0	0	0	0	0/1	1	0	0	0	0	0/1
HLA‐C16:01_DRB1_1501_P1	CoV_Prot_S_154‐168	CoV_Prot_S_121‐175	DRB1_1501	YSSANNCTF	5	0	0	1	1	1/5 (20)	6	0	0	0	0	0/6

*Note*: Rows refer to individual 9‐mer core peptides derived from *in vitro*‐ and *in silico*‐derived analyses of (potential) immunogenic 15‐mer SARS‐CoV‐2 spike, nucleocapsid, and membrane‐specific proteins. Columns from left to right refer to the individual core peptide ID, the respective ancestor 15‐mer peptide and pool, the predicted HLA restriction toward which the tests were performed, as well as the results of tests on PBMCs (6th to 11th column) and expanded cells (12th to 17th column).

### 2.2 Identification of Immunogenic Protein Regions and Peptides

**FIGURE 2:**
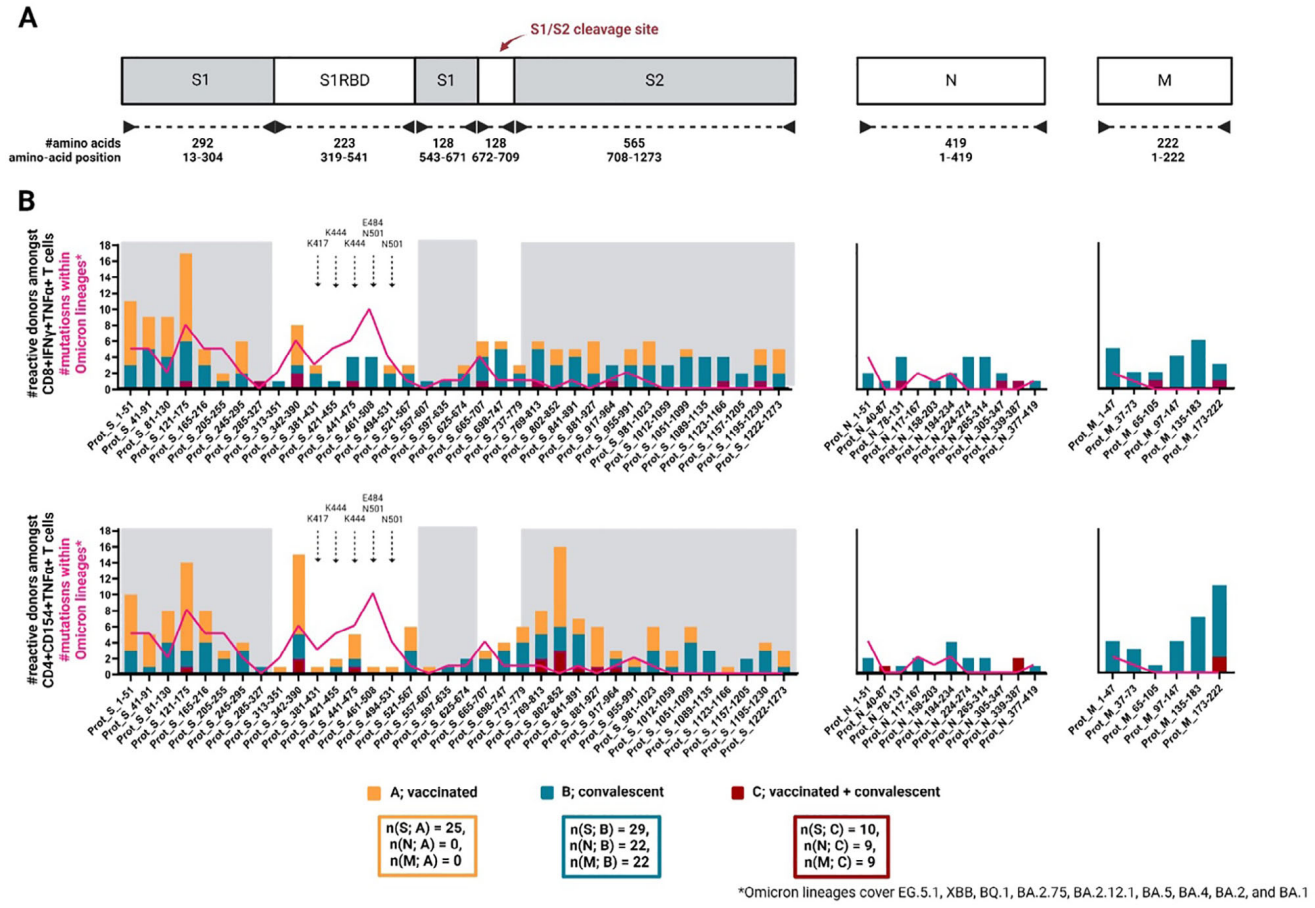
Immunogenic protein regions within the SARS‐CoV‐2 spike‐, nucleocapsid‐, and membrane‐protein among cohorts A, B, and C. (A) Illustration of SARS‐CoV‐2 Spike (S), Nucleocapsid (N) and Membrane (M) protein sequences. Given are the total numbers of amino acids covered by the protein (subunits) as well as their respective positions. (B) Bar graphs (aligned to (A)) for cohort A (vaccinated; orange), cohort B (convalescent; petrol) and cohort C (vaccinated and convalescent; red) showing the absolute numbers of reactive study subjects amongst CD8^+^TNF‐α^+^IFN‐γ^+^ (upper graphs) and CD4^+^CD154^+^TNF‐α^+^ T cells (lower graphs) (left *y*‐axes) upon stimulation with peptide pools covering the Spike protein (left graphs), the nucleocapsid protein (middle graphs) and the Membrane protein (right graphs), plotted against the respective protein region (*x*‐axes). Aligned to these bars, the absolute number of mutations that are found within the SARS‐CoV‐2 B.1.1.529/Omicron sublineages E.G.1, XBB, BQ.1, BA.2.12.1, BA.5, BA.4 and BA.1 (pink curve; left *y*‐axes) is shown.

To identify SARS‐CoV‐2‐specific immunity gained through vaccination or infection, the absolute numbers of study subjects, who showed T‐cell responses to the indicated 50‐amino‐acid‐long regions of the SARS‐CoV‐2 S, M, and N proteins were counted (Figure 2). T‐cell reactivity was measured based on CD8^+^IFN‐γ^+^TNF‐α^+^ T cells (from now on referred to as “reactive CD8^+^ T cells”) (Figure 2B, upper graph) and CD4^+^CD154^+^TNF‐α^+^ T cells (from now on referred to as “reactive CD4^+^ T cells”) (Figure 2B, lower graph). Cohort C (red bars) was negligible due to low n numbers and little overlap in HLA distributions to cohorts A and B (Figure 1B). Comparison of cohort A (orange bars) and B (petrol bars) unveiled differences between vaccination and infection: vaccinated individuals showed peaks of reactivity within the mid‐region of the S1‐subunit for both, reactive CD4^+^ and CD8^+^ T cells, and at the N‐terminal region of the S1‐receptor binding domain (RBD) and S2‐subunit for reactive CD8^+^ T cells. In contrast, convalescent individuals displayed a generally more homogeneously distributed T‐cell reactivity along the S, N, and M proteins among CD8^+^‐reactive T cells. This was slightly different for reactive CD4^+^ T cells, as here convalescent individuals display a partial lack of reactivity within the S1 RBD subunit, as well as a peaking region of reactivity at the C‐terminus of the Membrane protein.

To uncover whether virus mutations would occur preferentially in regions that could mount a T‐cell response, the number of mutations emerging from the B.1.1.529/Omicron virus strain lineages (EG.5.1, XBB, BQ.1, BA.2.75, BA.2.12.1, BA.5, BA.4, and BA.1) within the respective protein regions (Figure 2B, left *y*‐axes, pink lines) was aligned. Only partial overlap with the identified regions of T‐cell reactivities was observed.

Taken together, T‐cell responses in a cohort of convalescent individuals were mostly distributed over the entire sequences of the S, M, and N proteins, while vaccinated individuals rather reacted to distinct regions of the S protein. Our analyses provided a comprehensive overview of the epitope landscape of the S, M, and N protein of SARS‐Cov‐2 and identified certain differences between vaccinated and convalescent individuals. Recent mutations in SARS‐CoV‐2 did not indicate preferential mutations in regions capable of mounting strong T‐cell responses. Furthermore, a considerable number of potential MHC‐binding peptides were predicted and detected in all antigens. Hence, we conclude that T‐cell responses ought to be rather resilient to viral variants with the current rate of mutation of SARS‐CoV‐2 and a T‐cell escape variant affecting a general public would require a major reorganization of the viral protein structure, which is in line with previous studies [10]

### 2.3 (Un)favorable Haplotypes Do Not Influence HLAs’ Peptide Presentation Capacity

Fischer et al. [11] recently correlated certain HLA haplotypes with a longer disease duration and enhanced severity. To uncover whether the cause of a severe disease course lies in an impaired potential of the HLAs to present SARS‐CoV‐2 S, M, and N‐derived peptides, an independent cohort of convalescent donors, the so‐called cohort D, was recruited (Figure 1C). Cohort D was included to represent the first and second line of infections (“Ischgl‐cohort”). Therefore, this cohort is a cohort, with known immune and infection status, as well as disease status. Moreover, the patients are young and do not have co‐morbidities as they are potential convalescent blood donors. These individuals were sequenced for their HLA haplotypes and assessed for their cellular and humoral immune responses. Thereafter, the identified HLA haplotypes expressed by the individuals of cohort D were correlated to the individual disease duration and severity, which was assessed based on a self‐questionnaire (see extract in the M&M part). This correlation and the previous study by Fischer et al. [11] including a much bigger cohort, allowed a classification of the HLA haplotypes into two major groups: favorable and unfavorable haplotypes. The two groups were classified by calculating a hazard score for all individuals of cohort D based on their disease course (Table S3 and S4).

Having identified those haplotypes that are linked to mild and more severe COVID‐19 disease (Table S5), individuals from cohorts A to C were re‐grouped according to their expression of favorable‐ and unfavorable‐HLA haplotypes and subsequently re‐analyzed to see whether those HLAs elicit an altered T‐cell‐response pattern. However, the resulting T‐cell immunogenic protein regions were comparable for individuals of all cohorts and both subgroups (Figure S3). This assertion is applicable to both, reactive CD8^+^ and CD4^+^ T cells, pointing toward a similar MHC‐mediated peptide presentation capacity within both groups of individuals. A reciprocal analysis comparing the number of presented epitopes in people, who underwent severe or mild disease courses only revealed minimal differences, with significantly higher peptide responses for the more favorable MHC II haplotypes (Figure S4), providing further evidence for similar peptide presentation potentials in mild and severe diseases.

### 2.4 HLA Haplotypes Linked to Unfavorable Disease Courses Correlate With Reduced CD8^+^ T‐Cell Responses

**FIGURE 3:**
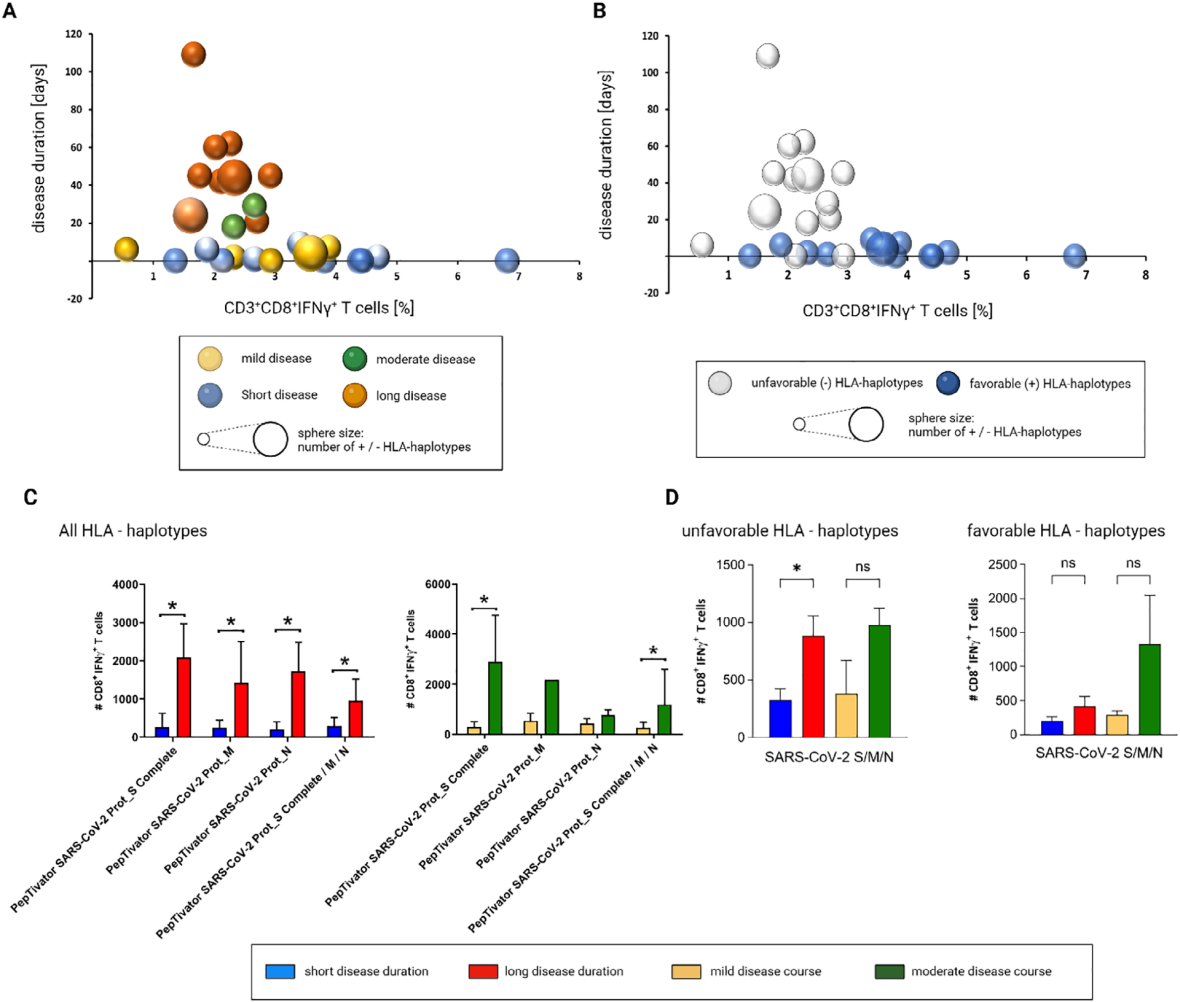
Long disease courses are dominated by a weaker virus‐specific CD8^+^ T‐cell response and unfavorable HLA‐haplotypes. Correlation of disease duration with the frequency of PepTivator‐specific CD3^+^CD8^+^IFNγ^+^ T cells of (A) study subjects with mild (yellow), moderate (green), short (blue), and long disease (red) (*n* ≥9/group) and (B) all study subjects (*n* = 40) show an accumulation of unfavorable HLA haplotypes and low frequencies of reactive CD8^+^ T cells for prolonged disease courses. White spheres indicate unfavorable HLA‐haplotypes, while blue spheres represent favorable HLA‐haplotypes for disease severity Frequencies were determined within PBMCs, gated on CD45, CD3, CD8, and the expression of IFN‐γ. (C) Flow cytometric assessment of the total cell count of virus‐reactive CD3^+^CD8^+^ T cells after stimulation with the three different SARS‐CoV‐2 PepTivators S, M, and N as well as a combination of all shows significantly increased numbers of reactive CD8^+^ T cells. (D) Assessment of the total cell count of virus‐reactive CD3^+^CD8^+^ T cells of study subjects bearing exclusively unfavorable (left) or favorable (right) HLA‐haplotypes after combinatorial restimulation with SARS‐CoV‐2‐PepTivator pool S, M and N shows significant differences for individuals with unfavorable HLA haplotypes only. *p < 0.05; Error bars represent mean values ± SEM; Mann–Whitney *U*‐test.

**FIGURE 4:**
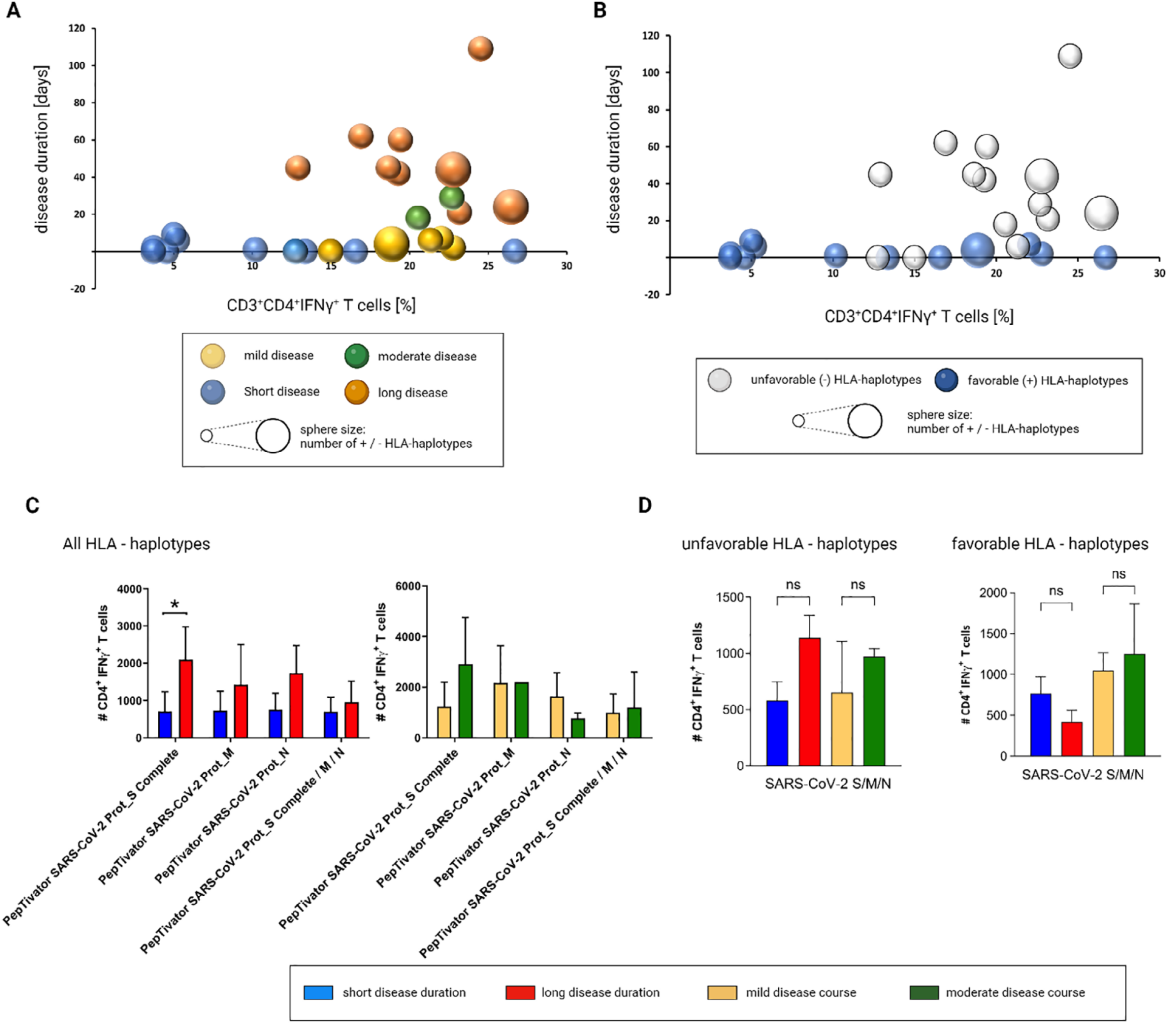
Long disease courses show higher frequencies of virus‐specific CD4^+^ T cells and unfavorable HLA‐haplotypes. (A) Correlation of disease duration with the frequency of PepTivator‐specific CD3^+^CD4^+^IFNγ^+^ T cells of (A) study subjects with mild (yellow), moderate (green), short (blue), and long disease (red) (*n* ≥9/group) and (B) all study subjects (*n* = 40) show an accumulation of unfavorable HLA haplotypes and a tendency toward elevated frequencies of reactive CD4^+^ T cells for prolonged disease courses. White spheres indicate unfavorable HLA haplotypes, while blue spheres represent favorable HLA haplotypes for disease severity. Frequencies were determined within PBMCs, gated on CD45, CD3, CD8, and the expression of IFN‐γ. (C) Flow cytometric assessment of the total cell count of virus‐reactive CD3^+^CD4^+^ T cells after stimulation with the three different SARS‐CoV‐2 PepTivators S, M, and N as well as a combination of all, shows only minor differences in reactive CD4^+^ T cell counts. (D) Assessment of the total cell count of virus‐reactive CD3^+^CD4^+^ T cells of study subjects bearing exclusively unfavorable (left) or favorable (right) HLA‐haplotypes after combinatorial restimulation with SARS‐CoV‐2‐PepTivator pool S, M and N demonstrates no significant differences. *p < 0.05; Error bars represent mean values ± SEM; Mann–Whitney *U*‐test.

As we did not find indications pointing to differences in peptide‐presentation capacity between individuals expressing favorable and unfavorable HLA alleles for cohorts A to C, we hypothesized that T‐cell intrinsic differences and varying strengths of the T‐cell responses were the underlying cause of varying disease courses. Hence, cellular responses in patients with favorable and unfavorable HLA haplotypes were analyzed for cohort D and the frequencies of CD3^+^CD8^+^INF‐γ^+^ as well as CD3^+^CD4^+^INF‐γ^+^ effector memory T cells were assessed and correlated with the individual disease duration or severity. This analysis revealed that patients with an unfavorable HLA‐haplotype showed lower frequencies of INF‐γ‐expressing SARS‐CoV‐2‐specific CD8^+^ T cells (with around 3%), although these patients suffered longer from COVID‐19 (Figure 3A,B). Noteworthy, blood withdrawal was always around the same time point (see material and methods section). Simultaneously, the same group suffering from long disease duration presented with high frequencies of INF‐γ‐expressing SARS‐CoV‐2‐specific CD4^+^ T cells (with up to approx. 28%), (Figure 4A,B). However, when the study subjects were not specifically categorized into favorable or unfavorable HLA‐haplotypes, disease duration as well as disease severity aligned with an increased number of CD3^+^CD8^+^INF‐γ^+^ as well as CD3^+^CD4^+^INF‐γ^+^ antigen‐specific T cells for all SARS‐CoV‐2 proteins (S, M, and N) after re‐stimulation (Figures 3C and 4C). Considering only the unfavorable HLA‐haplotypes for all disease stages, a significant increase in the absolute number of CD3^+^CD8^+^INFγ^+^ T cells was identified for those individuals that suffered from a long disease duration (Figures 3D and 4D), pointing toward a bystander T‐cell activation [17].

### 2.5 HLA‐alleles Correlated With Disease Duration and CD4^+^ T‐Cell Response

**FIGURE 5:**
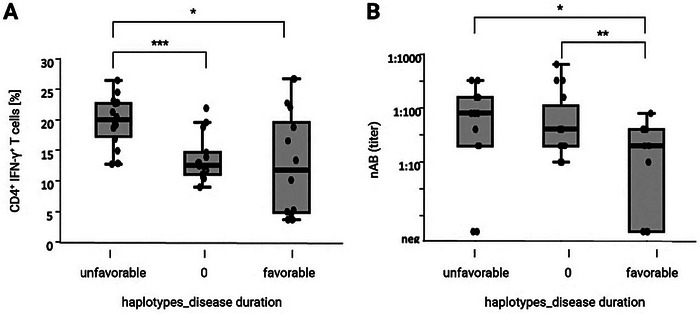
Unfavorable HLA‐haplotypes correlate with a higher CD4^+^ T‐cell response and elevated levels of neutralizing antibodies. (A) Correlation of disease duration with the CD4^+^ T‐cell response shows positive association with unfavorable HLA‐haplotypes. (B) Positive correlation of disease duration with the titer of neutralizing antibodies (nABs) within unfavorable HLA‐haplotypes. 0 corresponds to the median response of unfavorable and favorable haplotypes that were linked to disease duration; *p < 0.05, **p < 0.005, ***p < 0.0005. Error bars represent mean values ± SEM.

Although the unfavorable HLA‐haplotypes did not show a direct impact on CD4^+^ T‐cell responses within cohort D, the question was raised whether other parameters might play a role. Therefore, we looked at the age, and expression of CCR5, a chemokine receptor that is crucial for the humoral response of mucosal immunity [11], as well as the humoral response itself. There were no differences within cohort D under consideration of age or the expression of CCR5. As virus‐specific CD4^+^ T helper‐cell responses were elevated during long COVID‐19 disease courses within the study subjects of cohort D, we assessed their neutralizing antibody (nAB) titers. Titers of ≥1:160 were considered as a positive reaction against SARS‐CoV‐2 infections. Here we correlated the CD4^+^ T‐cell response of all study subjects with their individual disease duration and the associated HLA‐haplotypes, showing that those with the unfavorable HLA‐haplotypes presented also with the highest counts of CD4^+^ T cells (Figure 5A). Considering the level of nABs in correlation to the haplotypes, those individuals with unfavorable haplotypes correlated also with the highest titers of neutralizing antibodies (Figure 5B).

## 3 DISCUSSION

### 3.1 Mapping of Immunogenic Protein Regions Reveals Distinct Patterns for Vaccinated and Naturally Infected Individuals

Virus‐specific T‐cell responses are linked to fast viral clearance and mild disease courses. Improving our understanding of SARS‐CoV‐2‐specific T‐cell responses is crucial for the ability to treat and prevent severe disease courses. The present study provides a comprehensive overview of the epitope landscape of the N, M, and S proteins of SARS‐CoV‐2.

Dörnte et al. could previously show via both *in vitro* peptide stimulation and *in silico* NetMHCpan analyses that SARS‐CoV‐2 mutations may not lead to a complete escape from the T‐cell response on a population level. Since December 2020 [12], multiple vaccines have been developed to protect the population from severe infections [13]. However, through the continuous mutation of SARS‐CoV‐2 antigens novel virus strains arise with altered pathogenicity, as these strains bear the potential to escape SARS‐CoV‐2 specific immunity gained via vaccination with first‐generation vaccines or via natural infection with ancestral virus‐strains [1]. Therefore, predicting the effect of mutations on T cell‐mediated immune responses is important to estimate the potential loss of immunoprotection, for example, caused by a novel heavily mutated SARS‐CoV‐2 strain.

The present study demonstrates that vaccinated individuals have a tendency to mount T‐cell responses to slightly more distinct regions of the Spike protein, while T‐cell responses in naturally infected individuals were induced via more uniformly distributed regions of the S protein. Note, that here T‐cell responses have been evaluated independently from the individual response strength, and that the number of T‐cell epitopes might not correlate with the latter. This uniform distribution of protein regions that are recognized by T cells of convalescent individuals could also be observed for the M and the N proteins. Notably, this difference between vaccinated and convalescent individuals was especially pronounced within the CD4^+^ T‐cell compartment. Whether CD4^+^ T‐cell activation could directly affect the quantity and quality of the humoral immune response remains to be investigated, since follicular T helper (T_FH_) cells were not analyzed. However, the direct correlations between CD4^+^ T‐cell and humoral responses have been described [14, 15]. Therefore, a lack of CD4^+^ T cells that specifically recognize cognate peptides presented by the pre‐activated B cells may result in an impaired formation of long‐lived memory B cells. The broad and more evenly distributed T‐cell response presented by convalescent individuals might support an improved B‐cell response and memory phenotype compared with the immunity gained by vaccinated individuals, but as mentioned above differences between these two groups were rather mild, and an in‐depth T_FH_ analysis would be required.

Both the *in silico* prediction of potential MHC binders and the alignment of mutations found in the S, M, and N proteins suggest relative stability of the T‐cell response also for novel virus strains unless the protein structure of SARS‐CoV‐2 undergoes a major reorganization. This hypothesis is based on (I) the high number of potential MHC binding peptides predicted throughout the entire sequence of the Spike protein [16, 17], (II) the evenly distributed areas, which can mount T‐cell responses in naturally infected individuals, (III) the high number of 15‐mer and core peptides identified in the present study and other previously published literature [18, 19, 20, 21, 22, 23], and (IV) the absence of a correlation of past SARS‐CoV‐2 mutations with areas mounting strong T‐cell responses. This hypothesis is further supported by previously described findings, which demonstrated stable T‐cell responses to the B.1.1.529‐variant of SARS‐CoV‐2 in individuals vaccinated two or three times with first‐generation vaccines against all evaluated proteins [24].

To determine whether an impaired capacity of MHC alleles to present peptides to T cells was associated with more severe and prolonged disease, we reevaluated the T cell‐immunogenic protein regions of cohorts A to C. This analysis revealed that there is no difference between the T‐cell recognition patterns of individuals, expressing favorable or unfavorable HLA alleles. Based on that we evaluated the T‐cell response strength in an additional cohort D, for which clinical parameters for disease length and severity were available, which allowed us to perform additional correlations. To exclude the possibility of an impaired recognition of pMHC‐complexes by T cells future subsequent studies could comprise TCR avidity evaluations. Considering the T‐cell response of the naturally infected individuals categorized based on their disease duration and severity we identified a clear pattern for CD8^+^ T‐cell responses. Those individuals with a short disease duration mounted the highest frequencies of CD8^+^ T cells while those with a prolonged disease course had rather low frequencies of virus‐specific CD8^+^ T cells. Contrastingly, we observed high numbers of reactive, virus‐specific CD8^+^ T cells after re‐stimulation with SARS‐CoV‐2 derived peptides although the same cell numbers were applied for the assays, which points toward a high bystander activation of CD8^+^ T cells in severe COVID‐19. Additionally, comparing those observations to the T‐cell responses assessed for vaccinated‐only patients, the virus‐specific T cells generated under infection were directed against all proteins of the SARS‐CoV‐2 strain (S, M, and N).

This data indicates a lack of a virus‐specific MHC class I response in those patients with more severe symptoms, which is also supported by the literature [25, 26, 27]. Assessment of the virus‐specific CD4^+^ T‐cell response reveals an equal distribution in all four different subgroups of convalescent subjects, with elevated frequencies only in a combined cocktail of all proteins pointing toward a rather CD4‐mediated immune response in those patients who suffered longer. These findings are in line with the results found for neutralizing antibody titers, where those patients with a higher CD4^+^ T‐cell response also aligned with higher antibody titers, indicating a likely CD4^+^ T‐cell linked B‐cell response as described above. However, further characterization of CD4^+^ T‐cell subsets is required in the future.

Several publications have shown that infection with SARS‐CoV‐2 is, indeed, linked to the expression levels of HLA molecules. The group of Bahlmann et al. transfected two human cell lines, Caco‐2 (human colorectal adenocarcinoma cells) and Calu‐3 (human airway epithelial cells) with different SARS‐Cov2 virus strains and showed, that the virus‐positive cells expressed a significantly lower number of HLA‐molecules than nontransfected cells, indicating that SARS‐CoV‐2 inhibits the induction of the HLA class I pathway [28]. In a cohort of vaccinated patients, Mentzer et al. analyzed the humoral response by determining the antibody titers after vaccination and associated the response with the HLA class I pathway as they found a clear association with genes regulated on Chromosome 6, where the gene for HLA is located. Moreover, they identified that patients with HLA‐DQB1:06 presented with the highest antibody titers [29].

The correlation of the antibody titers with the severity of the disease revealed that while IgG titers against the Spike protein or the Nucleocapsid were clearly associated with the course of the disease, as neutralizing antibodies were only highly produced by those patients with a rather moderate/long infection (WHO grade 3) [30]. A more detailed study performed by Lehmann et al. investigated a so‐called “house‐cohort” in which some individuals of the same household underwent COVID‐19 infection, while others did not. They identified that those patients that got infected were homozygous for specific HLA molecules. Looking for those HLA‐molecules in convalescent study subjects of cohort D, carrying HLA‐DQB*05:01 was considered a “protective” HLA‐molecule, resulting in a shorter disease duration, while those lacking this molecule suffered from a longer infection [31]. Moreover, individuals with HLA‐DQB*05:01 expression had lower IgG antibody titers compared with those without, but there was no difference in the neutralizing antibody titers, indicating that carriers of HLA‐DQB*05:01 have a tendency to form antibodies [32].

Considering another parameter, that might play a role in the differing immune responses in cohort D, the cohort was divided into male and female donors to assess the effect of the sex on the cellular and humoral immune response. It is known that men generally develop slightly dominating Th1‐driven immune responses, while female responses are dominated by Th2‐cells [33]. Moreover, it has been reported that women mount much higher antibody levels due to more proliferative B cells [33]. An effect that was mirrored in the results of convalescent study subjects from cohort D. Through breaking down the HLA‐haplotypes to the most common alleles and looking at the duration of the SARS‐CoV‐2 infection, in females HLA class I haplotypes were associated with shorter, while HLA class II are associated with longer disease duration. Contrastingly, in males, HLA class II is associated with a shorter disease duration. Hence, a shorter disease duration is associated with a more favorable HLA‐haplotype (Table S3) [32]. These results are in line with the cellular response, in which unfavorable HLA‐haplotypes show a weaker CD8^+^ T‐cell response (Figure 3), while there was no association with CD4^+^ T cells (Figure 4). However, considering the disease duration, CD4^+^ T cells were elevated and were associated with elevated neutralizing antibody titers (Figures 4 and 5).

The results presented in this study underline the requirement of a proper cellular response, which is mandatory to clear the infection, thereby also supporting previous publications [34]. A strong T‐helper cell link within more severe and longer infections, which support the B cells in producing higher antibody titers is likely, however, requires further corroboration by future studies, in particular, the analysis of the T_FH_ subset. In addition, despite a relatively stable T‐cell response toward novel SARS‐CoV‐2 strains is expected, next‐generation vaccines should in the future aim for preserving a sufficient cellular T‐cell response and therefore should specifically include T‐cell antigens in addition to the formation of antibodies against SARS‐CoV‐2.

### 3.2 Limitations of the Study

1. Cohorts A, B, and C include nonsimilar numbers of participants as cohort C is considerably smaller than A and B (*n*_A_ = 27, *n*_B_ = 33, *n*_c_ = 12). Therefore, a direct comparison of obtained T‐cell reactivity patterns with cohort C was not done in this study, although it has no effect on the information about epitope candidates and core peptides obtained from the analysis of cohort C.

2. Cohort A comprises 3.5 times more females compared with males. Hence, a discrimination between T‐cell responses in male and female individuals for cohorts A, B, and C was not performed.

3. The mean interval between vaccination and/or natural infection and the time of blood collection is not the same between the cohorts with a median of 50 ± 9 days. Furthermore, the majority (63%) of cohort A received BNT162b2 vaccines, while B was more diverse. Correlation of T‐cell responses with vaccine type and time since vaccination and/or infection was not possible.

4. Differences between HLA allotypes expressed by each cohort were observed for all HLA class I and II genes. Although these differences are inevitable due to the natural diversity of the human HLA gene locus, unequal T‐cell reactivity against SARS‐CoV‐2 structural proteins may be influenced by the different peptide binding affinities of the expressed HLAs rather than only by the immunization routes represented by the different cohorts.

The evaluation of intrinsic differences in T‐cell response strengths was done on convalescent individuals with mild to moderate infections and no severe infections. Nevertheless, our results are in line with studies including severe outcomes. A deeper characterization of T_FH_ cells as a potential link between the CD4^+^ T cell immune response and antibody‐producing B cells is necessary. This could be the focus of future studies.

## 4 Materials and Methods

### 4.1 Study Participants and Whole Blood Donations

Study participants were recruited at Miltenyi Biotec via an internal blood donation program, and the Medical School Hannover (MHH) (cohort A–C), as well as from volunteers of the University Clinic Düsseldorf (cohort D). The individual SARS‐CoV‐2 background of all study participants was assessed via questionnaires and summarized in Table S1. Participants 1 to 73 were assigned to the study's cohorts comprising (A) vaccinated, (B) convalescent, and (C) vaccinated and convalescent individuals (Figure 1C). Whole blood donations were drawn from all volunteers after informed consent (Miltenyi Biotec: 20200151; Medical School Hannover: 9255_BO_K2020; University Clinic Düsseldorf) and following the WMA Declaration of Helsinki regarding the ethical principles for medical research involving human subjects. Therefore, 100 mL whole blood was drawn using an EDTA‐coated BD Vacutainer (BD, Franklin Lakes, USA, cat. no. 367525). Donations from the MHH hospital were transported overnight to Miltenyi Biotec. Donations from Miltenyi employees as well as from volunteers of the University Clinic Düsseldorf were directly processed after blood drawing. There was a mean of 57 days for cohort A, a mean of 59 days for cohort B, and a mean of 41 days for cohort C between the date of vaccination or infection and the date of sample drawing. For cohort D, patients donated their blood samples as convalescent donors within 2 months after recovery.

It is important to note that study participants of cohort D were recruited and analyzed independently from cohorts A to C, for the evaluation of SARS‐CoV‐2–specific T‐cell response strengths of the 40 donors depicted in this study. The categorization of those study participants into subgroups of mild, moderate, as well as short and long disease was done according to information assessed by a self‐questionnaire. Hereby, the different disease durations/strengths were subdivided based on disease duration and symptoms (mild: sore throat, running nose, no fever/ short: 1–10 days/moderate: sore throat, running nose, fever over 3 days/long: 20–120 days). We insert here an extract from the questionnaire for recording the degree of illness: 
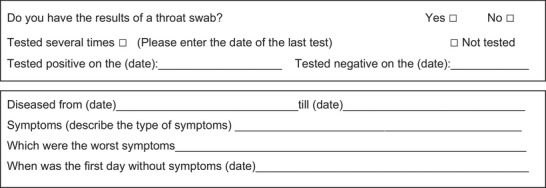


### 4.2 Sequential Walk

To identify SARS‐CoV‐2 specific T‐cell epitopes an *in vitro* stimulation approach was utilized to assess the immunogenicity of SARS‐CoV‐2 derived peptides (for sequences for SARS‐CoV‐2 peptides see GenBank QHD43416.1, QHD43423.2, QHD43419.1). By using an experimental design aiming for a stepwise downsizing/filtering of potential immunogenic regions, the number of necessary experiments and blood material could be reduced. Hence, in the first step, 15mer‐peptide pools, followed by a downstream assessment of single, 15mer peptides out of reactive pools, and finally the evaluation of 9mer core peptides derived from reactive single peptides have been evaluated for their individual potential to initiate a peptide‐specific T‐cell response.

In more detail, first, a sequential walk over the virus’ spike, nucleocapsid, and membrane protein was performed using mostly 15mer consecutive peptides having 11 amino acids overlapping each other. Due to a limited number of available peripheral blood mononuclear cells (PBMCs), this first peptide stimulation approach was done using pools, comprising 10 to 12 peptides. Together 36 peptide pools (362 single 15mer peptides) for the Spike, 11 peptide pools (110 single 15mer peptides) for the Nucleocapsid, and 6 peptide pools (60 single 15mer peptides) representing the Membrane protein cover the complete sequence of the respective protein. After evaluation of immunogenic peptide regions covered by the tested pool, the respective single peptides’ immunogenicity was determined following the similar *in vitro* stimulation procedure, on expanded PBMCs of the same study subject. From an additional *in silico*, NetMHC (II)pan‐based analysis of observations from these first two *in vitro* approaches, the peptides’ HLA restrictions and core peptide sequences could be extracted and used in a third, and final validation of core peptides’ immunogenicity for certain HLA alleles.

For each of the described stimulation experiments, an unstimulated control, as well as two positive controls stimulated with CytoStim (Miltenyi Biotec, cat. no. 130‐092‐172) and SARS‐CoV‐2 PepTivators (PepTivator SARS‐CoV‐2 Prot_S Complete, cat. no. 130‐127‐951; PepTivator SARS‐CoV‐2 Prot_M, cat. no 130‐126‐709; PepTivator SARS‐CoV‐2 Prot_N, cat. no 130‐126‐698) covering the complete sequence of the before mentioned structural proteins, were prepared.

### 4.3 Isolation of Peripheral Blood Mononuclear Cells

To isolate the PBMCs from whole blood, density gradient centrifugation using Pancoll (Pan Biotec, Aidenbach, Germany, cat. no. P04‐60500) was utilized according to the manufacturer's instructions using CliniMACS PBS/EDTA buffer (Miltenyi Biotec, Bergisch Gladbach, Germany, cat. no. 200‐070‐025). To complete the isolation procedure, cells collected in a 50 mL Falcon tube were diluted with CliniMACS PBS/EDTA buffer and centrifuged twice at 200*g* for 15 min to remove the remaining thrombocytes. Afterward, cells were counted using the Sysmex XP‐300 device (Sysmex, Norderstedt, Germany) and diluted to a concentration of 1 × 10^7^ cells/mL using RPMI‐1640 Medium (Biowest, Nuaillé, France, cat. no. L0501‐500) supplemented with 5% human AB serum (Capricorn, Ebsdorfergrund, Germany, cat. no. HUM‐3B, Lot. CP20‐3472) and 1× Gibco Anti‐Anti (Thermo Fisher Scientific, Waltham, USA, cat. no. 11580486) (from now on referred to as “supplemented medium”). Using this cell suspension, an appropriate number of wells of 96‐well flat bottom plate (Falcon, New York, USA, cat. no. 353072) were filled to a concentration of 1 × 10^6^ PBMCs per well. The plate was then incubated overnight at 37°C, 5% CO_2_.

### 4.4 *In Vitro* Stimulation Approach and Subsequent Staining of Activity Markers and Intracellular Cytokines

Each peptide (pool) was separately added at a final concentration of 1 µg/mL/peptide/1E6 PBMCs to the priorly prepared cell samples. Stimulation was done for a total of 6 h at 37°C, 5% CO_2._ After 2 h, 2 µg/mL Brefeldin A (Sigma‐Aldrich, St. Louis, USA, cat. no. B7651) was added to each well to prevent cells from cytokine secretion.

After complete stimulation, staining was performed in a 96‐well V‐bottom plate (Sigma‐Aldrich, cat. no. Z667234) into which cells were transferred after adding 100 µL PBS/EDTA (2 mM) buffer to each sample. Afterward, the plate was centrifuged at 300*g* for 5 min at room temperature (RT) and the supernatant was discarded. Next, cells were stained with Viobility 450/452 Fixable Dyes (Miltenyi Biotec, cat. 130‐109‐816), according to the manufacturer's instructions. To enable subsequent intracellular staining of cytokines and activation markers, cell fixation was performed using Inside Fix (Inside Stain Kit, Miltenyi Biotec, cat. no. 130‐090‐477), according to the manufacturer's instruction, followed by resuspension in 250 µL/well Inside Perm (Inside Stain Kit, Miltenyi Biotec, cat. no. 130‐090‐477) and centrifugation at 300*g* for 5 min at RT. Finally, cells were stained using the following antibody‐cocktail: anti‐CD3—APC (Miltenyi Biotec, cat. no. 130‐113‐135), anti‐CD14—VioBlue (Miltenyi Biotec, cat. no. 130‐110‐525), anti‐CD20—VioBlue (Miltenyi Biotec, cat. no. 130‐111‐531), anti‐CD4—VioBright515 (Miltenyi Biotec, cat. no. 130‐114‐535), anti‐CD8—VioGreen (Miltenyi Biotec, cat. no. 130‐110‐684), anti‐IFN‐γ—PE (Miltenyi Biotec, cat. no. 130‐113‐496), anti‐TNF‐α—PEVio770 (Miltenyi Biotec, cat. no. 130‐120‐492), anti‐CD154—APCVio770 (Miltenyi Biotec, Cat. No.130‐114‐130), and anti‐IL‐2—PEVio615 (Miltenyi Biotec, cat. no.130‐111‐307). All antibodies were used in a 1:50 dilution. Staining was done according to the manufacturer's instructions. After additional washing with 100 µL Inside Perm and centrifugation at 300*g* for 10 min at RT, cells are resuspendedin250 µL PBS/EDTA (2 mM) buffer supplemented with 0.5% of MACS BSA Stock Solution (Miltenyi Biotec, cat. no. 130‐091‐376). Flow cytometric analysis was done using the MACSQuant16—flow cytometer (Miltenyi Biotec, cat. no. 130‐109‐803). We defined a positive T‐cell response by the appearance of a distinct population for the chosen activity‐associated markers and a frequency increase of at least 10% with respect to the unstimulated control.

### 4.5 Expansion of SARS‐CoV‐2 Spike, Nucleocapsid, Membrane‐Specific Reactive T Cells

Freshly isolated PBMCs (see previous section) were plated out on a four‐well plate (Falcon, Cat. No.353046), at a concentration of 5E6 PBMCs/5 mL supplemented medium. To support directed cell proliferation, 100 U/mL IL‐2 (Miltenyi Biotec, cat. no. 130‐097‐745) as well as 1 µg/mL of either PepTivator SARS‐CoV‐2 Prot_S Complete, PepTivator SARS‐CoV‐2 Prot_M, or PepTivator SARS‐CoV‐2 Prot_N were added to the cell suspension. Afterward, the cells were incubated for 14 days at 37°C, 5% CO_2_. Meanwhile, the medium was refreshed every 2 to 3 days by gentle removal of half the medium volume and subsequent addition of the same volume‐supplemented medium together with 100 U/mL IL‐2.

### 4.6 *In Silico* Peptide Binding Prediction

Using an *in silico* approach, HLA restrictions, corresponding binding affinities, and core sequences of single 15mer peptides, derived from previously immunogenic peptide pools, were specified. For that, the open‐source NetMHCpan (https://services.healthtech.dtu.dk/service.php?NetMHCpan‐4.1) and NetMHCIIpan (https://services.healthtech.dtu.dk/service.php?NetMHCIIpan‐4.0) prediction‐algorithms were utilized, for interactions with MHC class I and MHC class II molecules, respectively. The eluted ligand percentile ranks (EL‐Rank) are used as a measure of the peptide binding affinity. Therein, EL‐Ranks <2 and >0.5 are considered weak binding peptides, and EL‐Ranks <0.5 are considered strong binding peptides to the respective MHC class I molecule. For MHC class II molecules, instead, EL‐Rank thresholds of <5 and >2 and <2 are applied for the categorization into weak and strong binding peptides, respectively.

### 4.7 Sequencing of HLA‐Alleles

To genotype the HLA alleles for all cohort participants from the University Clinic Düsseldorf (cohort D), an amplicon‐based NGS‐based approach was used. Six multiplex polymerase chain reactions (PCRs) were used to amplify exons 2, 3, and 4 of the HLA‐Class I and HLA‐DPB1 genes and exons 2 and 3 of HLA‐DRB1, HLA‐DQA1, and HLA‐DQB1. The resulting fragments were supplemented with sample‐specific barcodes and Illumina‐compatible adapters. Sequencing was performed on an Illumina MiSeq device (Illumina Inc., San Diego, USA). A customized software (NGSSequence Analyser, Institute for Transplantation Diagnostics and Cell Therapy, University Hospital Dusseldorf, Dusseldorf, Germany) served for the analysis of the sequence reads using quality control values and high coverage to automate the data analysis. For haplotype phasing of the obtained HLA data, we used the Arlequin software (version 3.5.2.2, available cmpg.unibe.ch/software/arlequin35/), for binding affinities to viral peptides [35].

For all remaining study subjects from other cohorts, HLA sequencing was performed externally by DKMS Life Science Lab GmbH.

### 4.8 Determination of Antibody Levels

Determination of antibody levels was performed using two serological assays as described before [36]. Spike S1 protein domain‐specific IgA and IgG antibodies were detected by ELISA (Euroimmun, Germany), and Ig antibodies against the Nucleocapsid were verified by Roche Diagnostic Test (Elecsys). Neutralizing antibodies were tested additionally in biological duplicates. Heat‐inactivated donor plasma was diluted. Serum neutralization titer was determined by microscopic inspection as the highest serum dilution without virus‐induced cytopathic effect (CPE). Two previously tested sera from SARS‐CoV‐2 infected individuals served as positive controls for each round. A high‐titer control (NT 1:640) and a medium‐titer control (NT 1:160) were defined to validate each assay. Interassay and intra‐assay variations were determined, and these control sera exhibited maximum variation within only one dilution level. Serum from noninfected individuals and cells without serum served as negative controls to confirm virus‐induced CPE. A neutralization titer of 1:160 was defined as the threshold titer for the binary representation of virus neutralization capacity, allowing the potential plasmapheresis product to serve as a therapeutic agent.

### 4.9 *In Vitro* Assays for Antigen‐Specific T Cells

PBMCs of convalescent subjects from four different sub‐groups of disease severity or duration (short, mild, moderate, long) were isolated as described above. The cell suspension was adjusted to a cell titer of 1 × 10^7^ cells/mL X‐Vivo 15 medium (Lonza; cat. no. 11695120) and 100 µL/well was distributed into a 96‐well plate. For each study subject, several approaches were set up: SARS‐CoV‐2 PepTivator Prot_S Complete, Prot_M, and Prot_N as well as a well for all three PepTivators, a negative control (no PepTivators) and the stimulation with PMA/Iono as a positive control. Cells were incubated with the according stimulating agents for 6 h in total. After 30 min of stimulation, 5 ng/mL Brefeldin A (BioLegend; ca. no. 420601) were added to each well. Subsequently, cells were harvested and stained for anti‐CD3‐PE‐Dazzle (BioLegend, cat. no. 300446), anti‐CD4 BV650 (BioLegend, cat. no. 317436), anti‐CD8 VioGreen (Miltenyi Biotec, cat. no. 130‐113‐164), anti‐CD45RO_BV570 (BioLegend, cat. no. 304226), anti‐CD154_PE‐Cy7 (BioLegend, cat. no. 310832) and anti‐IFN‐γ‐BV421 (BioLegend, cat. no. 502532). All antibodies were used in a 1:100 dilution. Staining was done according to the manufacturer's instructions. After washing with PBS and centrifugation at 300*g*, cells are resuspended in 250 µL PBS/EDTA (2 mM) buffer supplemented with 0.5% of MACS BSA Stock Solution (Miltenyi Biotec, cat. no. 130‐091‐376). Flow cytometric analysis was done using the CytoFlex S–flow cytometer (Beckmann Coulter). Each sample was then normalized to the negative control to exclude unspecific responses or backgrounds.

## Author Contributions

Conceptualization: C. Dörnte, A. Datsi, J. Fischer, and M. Schuster. Sample acquisition: J. Fischer, J. Kostyra, C. Lamsfuß, H. Baurmann, B. Eiz‐Vesper, and A. Bonifacius. Investigation: C. Dörnte, A. Datsi, V. Traska, J. Kostyra, M. Wagner, O. Brauns, DS. Visualization: C. Dörnte and A. Datsi. Funding acquisition: H. Baurmann, A. Richter, J. Schmitz, and M. Schuster. Project administration: C. Dörnte, O. Brauns, H. Winkels, H. Baurmann, B. Eiz‐Vesper, A. Bonifacius, R. V. Sorg, C. Dose, J. Schmitz, A. Richter, J. Fischer, and M. Schuster. Supervision: M. Schuster, J. Fischer, and R. V. Sorg. Writing–original draft: C. Dörnte and A. Datsi. Writing–review & editing: C. Dörnte, A. Datsi, J. Fischer, R. V. Sorg, and M. Schuster.

## Conflicts of Interest

C. D., V. T., J. K., J. H., M. W., O. B., D. S., C. L., H. B., C. Do., J. S., A. R., and M. S. are employees of Miltenyi Biotec B.V. & Co. KG. The remaining authors declare no conflicts of interest.

### Peer Review

The peer review history for this article is available at https://publons.com/publon/10.1002/eji.202451497.

## Supporting information



Supporting Information

Supporting Information

Supporting Information

Supporting Information

Supporting Information

## Data Availability

All relevant data are included in the supplementary material. The data that support the findings of this study and are not included here, are available on request from the corresponding author.
