# Supplementary material for: Distinct HLA Haplotypes Are Associated With an Altered Strength of SARS‐CoV‐2‐Specific T‐Cell Responses and Unfavorable Disease Courses
Source: Eur J Immunol. 2025 Apr 21;55(4):e202451497. doi: 10.1002/eji.202451497 (PMC12012228; doi:10.1002/eji.202451497)
Supplement: Supplementary file 1 — Supporting Information [file EJI-55-e202451497-s001.docx]

**Supplementary Figure 1. HLA class I and class II-allotype distribution among cohort A (vaccinated), B (convalescent), C (vaccinated and convalescent) and D (convalescent).** Distribution pie charts for HLA-loci HLA-A, -B, -C, DPB1, -DQB1, -DRB1, and -DQA1 are given (from top to bottom) for each of the three cohorts A (vac; vaccinated), B (con; convalescent), C (vac+con; vaccinated and convalescent) and D (independent convalescent donor cohort from Duesseldorf) (from left to right). For each allotype the percentage of presenting donors within the respective cohort is given.
