# Supplementary material for: Distinct HLA Haplotypes Are Associated With an Altered Strength of SARS‐CoV‐2‐Specific T‐Cell Responses and Unfavorable Disease Courses
Source: Eur J Immunol. 2025 Apr 21;55(4):e202451497. doi: 10.1002/eji.202451497 (PMC12012228; doi:10.1002/eji.202451497)
Supplement: Supplementary file 2 — Supporting Information [file EJI-55-e202451497-s004.docx]

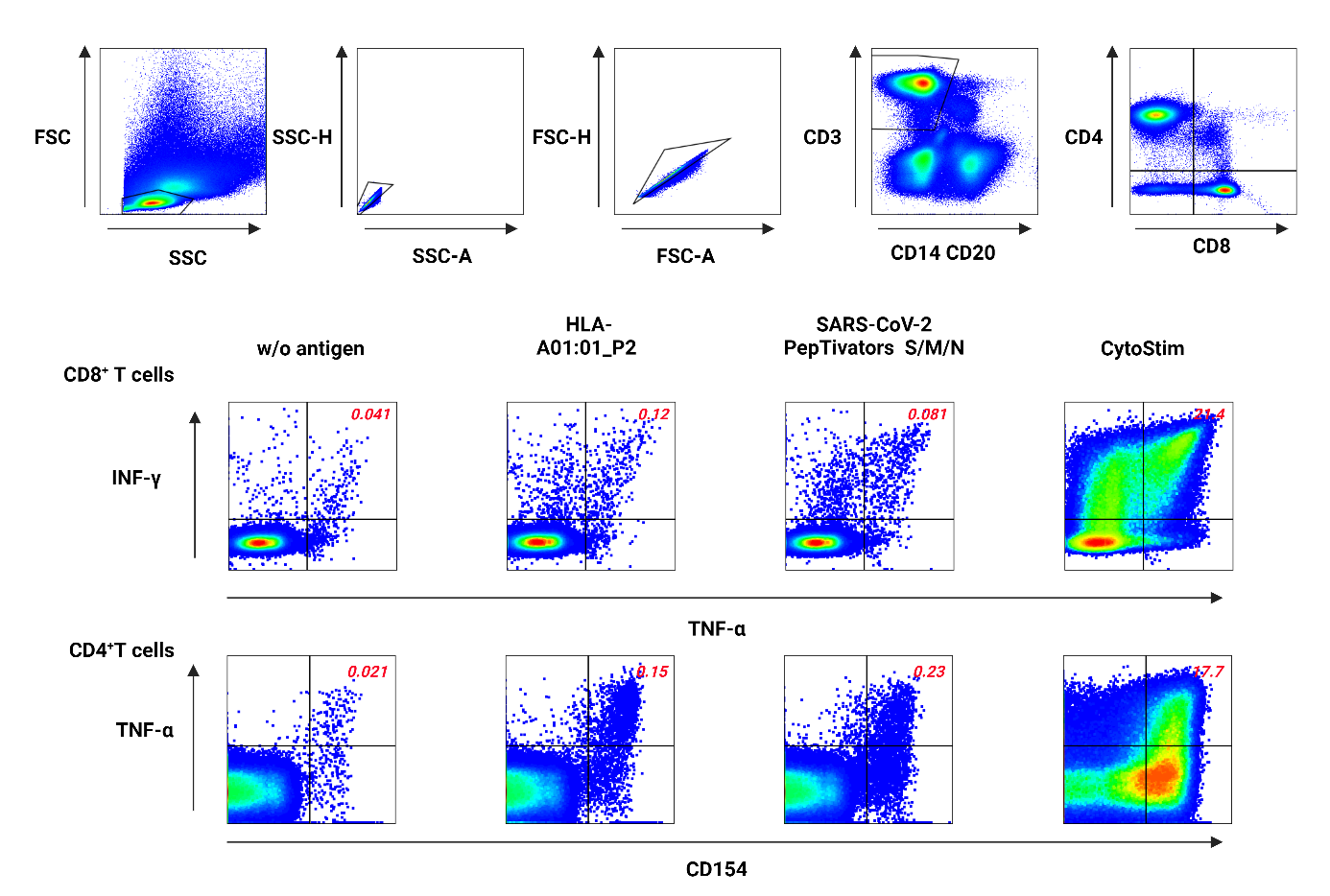


**Supplementary Figure 2. Exemplary flow cytometric gating strategy for the evaluation of T-cell responses after *in vitro* peptide stimulation**. Exemplary analysis of a SARS-CoV-2 experienced individual stimulated with 9-mer core peptide HLA-A0101_P2, SARS-CoV-2 PepTivator^®^ proteins covering the Spike, Membrane and Nucleocapsid protein and with CytoStim™. Pre-gating was performed on lymphocytes, twice exclusion of doublets, and CD3^+^ cells before differentiation into CD4^+^ and CD8^+^ single positive cells. Reactive CD8^+^ T cells are defined as INF-γ^+^TNF-α^+^-double positive cells. Reactive CD4^+^ T cells are defined as TNF-α^+^CD154^+^-double positive cells. Frequencies of double positive cells are indicated in red in the upper right quadrant.
