# Supplementary material for: Distinct HLA Haplotypes Are Associated With an Altered Strength of SARS‐CoV‐2‐Specific T‐Cell Responses and Unfavorable Disease Courses
Source: Eur J Immunol. 2025 Apr 21;55(4):e202451497. doi: 10.1002/eji.202451497 (PMC12012228; doi:10.1002/eji.202451497)
Supplement: Supplementary file 3 — Supporting Information [file EJI-55-e202451497-s003.docx]

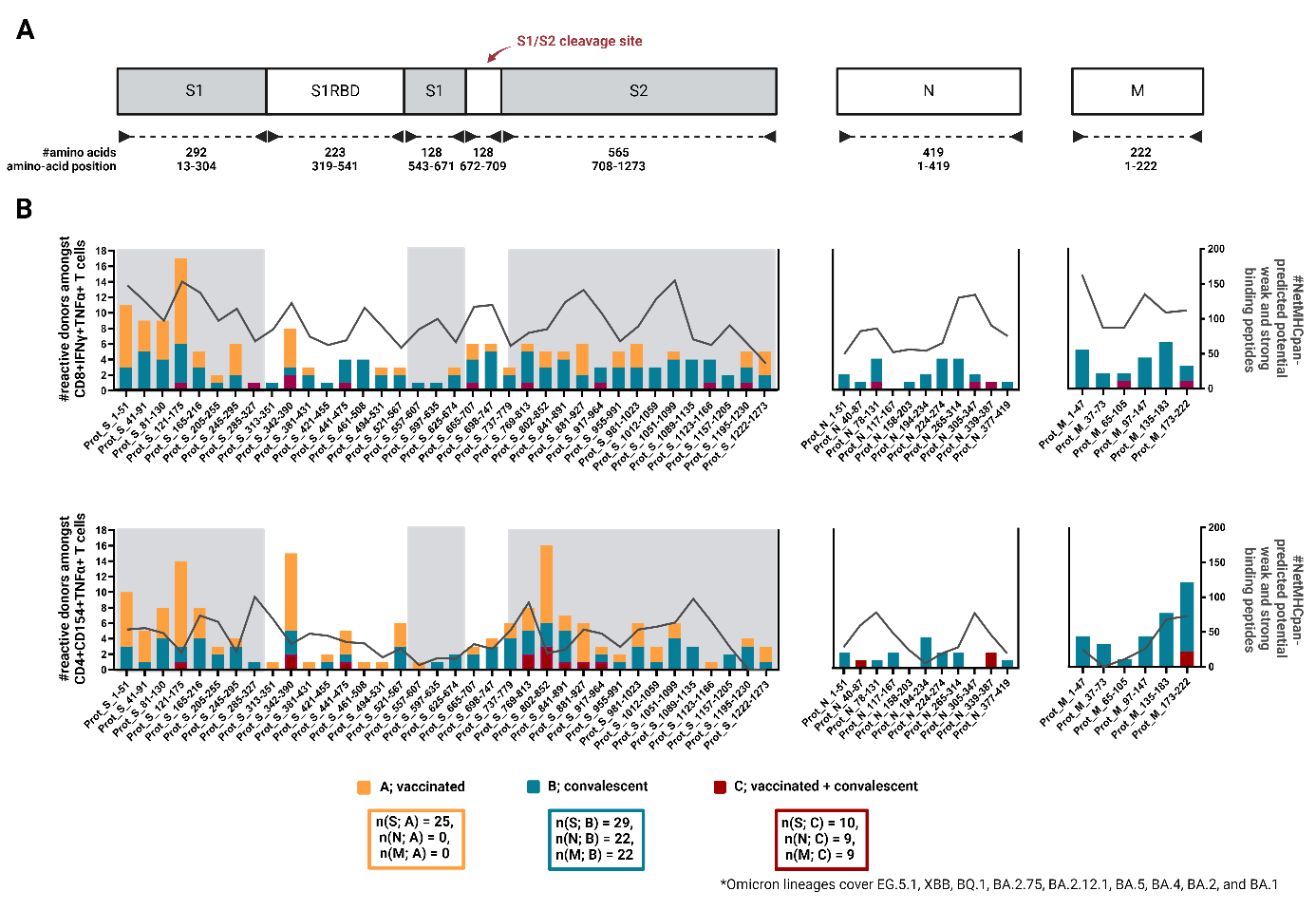


**Supplementary Figure 3. Number of predicted potential HLA-binding peptides aligned to immunogenic protein regions found within the SARS-CoV-2 Spike-, Nucleocapsid-, and Membrane-protein among cohort A, B and C. (A)** Illustration of SARS-CoV-2 Spike (S), Nucleocapsid (N) and Membrane (M) protein sequences. Given are the total numbers of amino acids covered by the protein (subunits) as well as their respective positions. (**B**) Bar graphs (aligned to (A)) for cohort A (vaccinated; orange), cohort B (convalescent; petrol) and cohort C (vaccinated and convalescent; red) showing the absolute numbers of reactive study subjects amongst CD8^+^TNF-α^+^IFN-γ^+^ (upper graphs) and CD4^+^CD154^+^TNF-α^+^ T cells (lower graphs) (left y-axes) upon stimulation with peptide pools covering the Spike protein (left graphs), the Nucleocapsid protein (middle graphs) and the Membrane protein (right graphs), plotted against the respective protein region (x-axes). Aligned to these bars, the number of NetMHCpan-predicted weak and strong binding peptides, which are found within the specific protein regions, are depicted (gray curve; right y-axes).
