# Supplementary material for: Distinct HLA Haplotypes Are Associated With an Altered Strength of SARS‐CoV‐2‐Specific T‐Cell Responses and Unfavorable Disease Courses
Source: Eur J Immunol. 2025 Apr 21;55(4):e202451497. doi: 10.1002/eji.202451497 (PMC12012228; doi:10.1002/eji.202451497)
Supplement: Supplementary file 4 — Supporting Information [file EJI-55-e202451497-s002.docx]

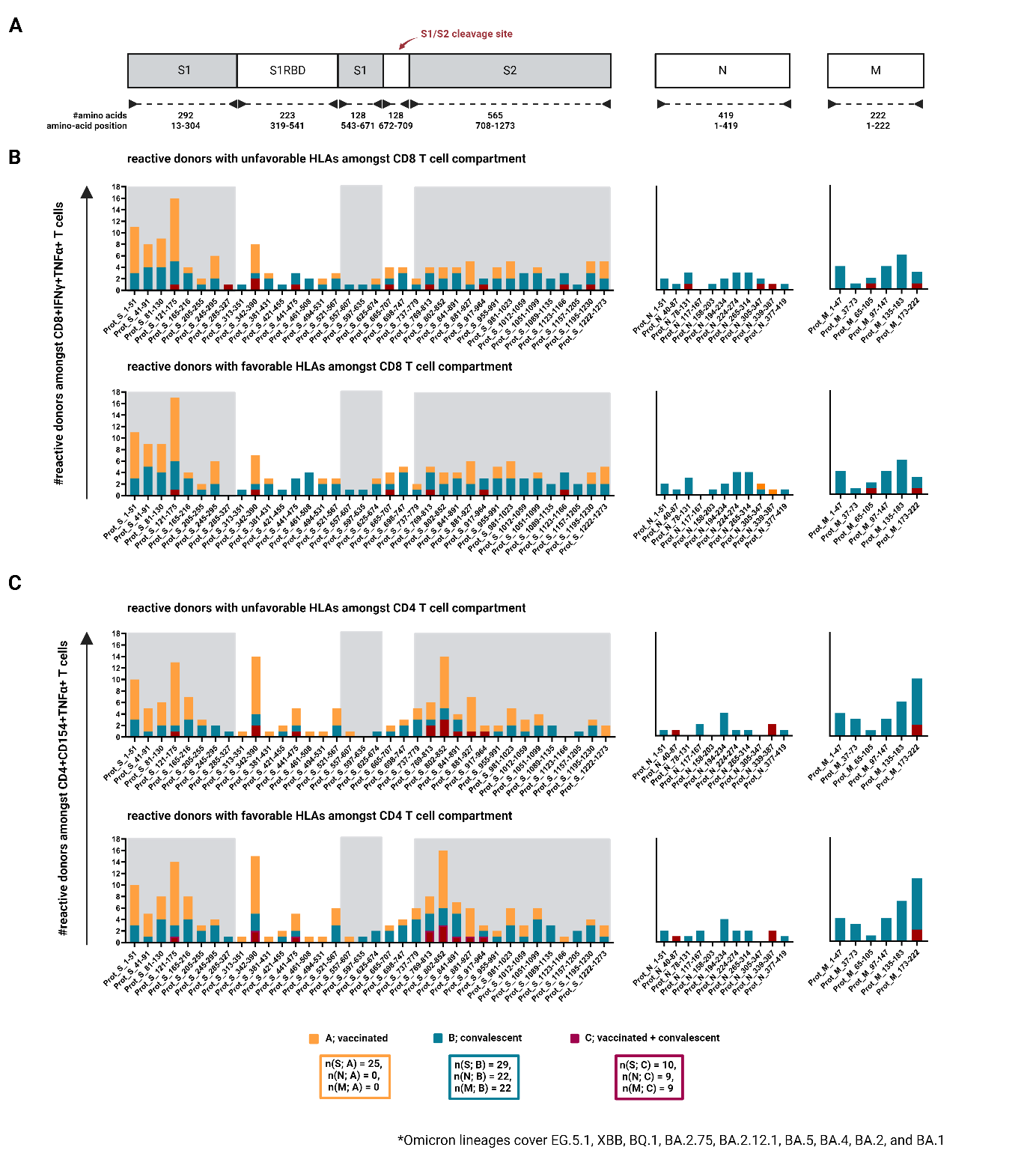


**Supplementary Figure 4. Immunogenic protein regions within the SARS-CoV-2 Spike, Nucleocapsid, and Membrane proteins among cohorts A, B and C under consideration of (un)favorable HLA alleles associated to severe and mild Covid-19 disease courses.** (**A**) Illustration of SARS-CoV-2 Spike (S), Nucleocapsid (N) and Membrane (M) protein sequences. Given are the total numbers of amino acids covered by the protein (subunits) as well as the amino-acids positions. (**B, C**) Bar graphs (aligned to (A)) showing the absolute number of reactive donors expressing unfavorable HLA allotypes (upper graphs) or favorable HLA allotypes (lower graphs) amongst CD8^+^TNFɑ^+^IFN-ɣ^+^ T cells (B) and CD4^+^CD154^+^TNF-ɑ^+^ T cells (C). Orange bars show vaccinated individuals from cohort A, while petrol bars show convalescent (cohort B) and red bars vaccinated and convalescent individuals (cohort C).
