# Supplementary material for: Distinct HLA Haplotypes Are Associated With an Altered Strength of SARS‐CoV‐2‐Specific T‐Cell Responses and Unfavorable Disease Courses
Source: Eur J Immunol. 2025 Apr 21;55(4):e202451497. doi: 10.1002/eji.202451497 (PMC12012228; doi:10.1002/eji.202451497)
Supplement: Supplementary file 5 — Supporting Information [file EJI-55-e202451497-s005.docx]

**Supplementary Table 1.** **Detailed background information on study subjects of cohorts A - D.**  Cohort description: A = vaccinated, B = convalescent, C = vaccinated and convalescent (second column), D = convalescent; the gender of the participants: m = male, f = female (third column); age (in years) refers to the age of the donor at the date of blood donation (fourth column); the indicated vaccine refers to the vaccine types administered as first and second dose (firth column); the sub-cohorts for long, short, mild and moderate disease courses for study subject of cohort D are indicated (sixth column); the interim time between last immunization via vaccination (referring to cohort A or C) or natural infection (referring to cohort B or C) is provided in days (seventh column); HLA allotypes expressed by each participant are shown for HLA Class I genes (HLA-A, -B, -C) as well as HLA class II genes (HLA-DPB1, -DQB1, -DRB1 and -DQA1) (seventh to thirteenth column); NI = no information, n.A. = not applicable

| **ID** | **cohort** | **gender** | **age** | **vaccine*** | **sub- cohort** | **interim (days)** | **HLA-A** | | **HLA-B** | | **HLA-C** | | **HLA-DPB1** | | **HLA-DQB1** | | **HLA-DRB1** | | **HLA-DQA1** | |
| --- | --- | --- | --- | --- | --- | --- | --- | --- | --- | --- | --- | --- | --- | --- | --- | --- | --- | --- | --- | --- |
| **1** | B | m | 42 | n.A. | n.A. | NI | *02:01 | *29:02 | *40:02 | *44:03 | *02:02 | *16:01 | *02:01 | *04:01 | *02:02 | *03:01 | *07:01 | *11:01 | NI | NI |
| **2** | B | f | 36 | n.A. | n.A. | 21 | *24:02 | *31:01 | *08:01 | *35:03 | *07:01 | *12:03 | *04:01 | *04:02 | *02:01 | *03:01 | *03:01 | *11:01 | *05:01 | *05:01 |
| **3** | B | f | 35 | n.A. | n.A. | 191 | *01:01 | *03:01 | *07:02 | *51:01 | *07:02 | *15:02 | *03:01 | *04:01 | *03:02 | *06:02 | *04:01 | *15:01 | NI | NI |
| **4** | B | f | 19 | n.A. | n.A. | 14 | *03:01 | *24:02 | *07:02 | *07:02 | *07:02 | *07:02 | *02:01 | *04:01 | *03:02 | *06:02 | *04:01 | *15:01 | NI | NI |
| **5** | B | f | 52 | n.A. | n.A. | 21 | *02:01 | *02:01 | *27:05 | *44:02 | *02:02 | *05:01 | *04:01 | *04:01 | *03:01 | *05:01 | *01:01 | *04:01 | NI | NI |
| **6** | B | m | 25 | n.A. | n.A. | 21 | *02:01 | *03:01 | *07:02 | *44:02 | *05:01 | *07:02 | *02:01 | *04:02 | *05:03 | *06:02 | *14:01 | *15:01 | NI | NI |
| **7** | B | f | 54 | n.A. | n.A. | 28 | *02:01 | *26:01 | *14:01 | *51:01 | *08:02 | *15:02 | *04:01 | *10:01 | *03:02 | *06:03 | *04:01 | *13:01 | NI | NI |
| **8** | B | m | 22 | n.A. | n.A. | 28 | *02:01 | *26:01 | *27:05 | *51:01 | *01:02 | *15:02 | *04:01 | *10:01 | *05:01 | *06:03 | *01:01 | *13:01 | NI | NI |
| **9** | B | m | 33 | n.A. | n.A. | 28 | *03:01 | *25:01 | *38:01 | *50:01 | *06:02 | *12:03 | *02:01 | *09:01 | *02:01 | *06:03 | *03:01 | *13:01 | NI | NI |
| **10** | B | m | 50 | n.A. | n.A. | 28 | *01:01 | *68:01 | *08:01 | *40:02 | *02:02 | *07:01 | *04:01 | *04:02 | *02:01 | *03:01 | *03:01 | *11:01 | NI | NI |
| **11** | B | m | 26 | n.A. | n.A. | 28 | *02:01 | *23:01 | *15:01 | *38:01 | *03:03 | *04:01 | *04:01 | *19:01 | *03:01 | *06:03 | *11:01 | *13:01 | NI | NI |
| **12** | B | m | 52 | n.A. | n.A. | 28 | *11:01 | *29:01 | *35:03 | *52:01 | *04:01 | *12:02 | *03:01 | *04:01 | *04:02 | *06:01 | *08:01 | *15:02 | NI | NI |
| **13** | B | m | 33 | n.A. | n.A. | 28 | *26:01 | *68:01 | *08:23 | *13:02 | *06:02 | *07:02 | *04:01 | *04:02 | *02:01 | *02:01 | *03:01 | *07:01 | NI | NI |
| **14** | B | f | 26 | n.A. | n.A. | 28 | *01:01 | *24:02 | *07:02 | *44:27 | *07:02 | *07:04 | *03:01 | *04:01 | *04:02 | *05:02 | *08:01 | *16:01 | NI | NI |
| **15** | B | m | 37 | n.A. | n.A. | 56 | *02:01 | *02:01 | *08:01 | *39:01 | *07:01 | *12:03 | *03:01 | *04:01 | *02:01 | *03:01 | *03:01 | *11:01 | *05:01 | *05:01 |
| **16** | B | f | 35 | n.A. | n.A. | 49 | *02:01 | *02:01 | *40:01 | *44:02 | *03:04 | *05:01 | *02:01 | *04:01 | *03:01 | *06:04 | *04:01 | *13:02 | *01:02 | *03:01 |
| **17** | B | f | 57 | n.A. | n.A. | 56 | *02:01 | *32:01 | *37:01 | *44:02 | *05:01 | *06:02 | *01:01 | *04:02 | *03:01 | *03:01 | *11:01 | *12:01 | *05:01 | *05:01 |
| **18** | B | f | 60 | n.A. | n.A. | 56 | *02:01 | *11:01 | *44:02 | *55:01 | *03:03 | *05:01 | *04:01 | *04:02 | *05:03 | *06:03 | *13:01 | *14:54 | *01:03 | *01:01 |
| **19** | B | f | 29 | n.A. | n.A. | n.A. | *02:01 | *03:01 | *14:02 | *39:06 | *07:02 | *08:02 | *04:01 | *05:01 | *03:02 | *06:09 | *04:01 | *13:02 | NI | NI |
| **20** | B | m | 48 | n.A. | n.A. | 56 | *02:01 | *02:01 | *07:02 | *08:01 | *07:01 | *07:02 | *02:01 | *03:01 | *02:01 | *03:01 | *03:01 | *04:01 | *03:01 | *05:01 |
| **21** | B | m | 47 | n.A. | n.A. | 56 | *02:01 | *02:01 | *45:01 | *57:01 | *06:02 | *06:02 | *02:01 | *04:01 | *03:02 | *03:03 | *04:05 | *07:01 | *02:01 | *03:01 |
| **22** | B | f | 54 | n.A. | n.A. | 56 | *02:01 | *03:01 | *44:02 | *47:01 | *05:01 | *06:02 | *04:01 | *04:01 | *05:01 | *06:02 | *01:01 | *15:01 | *01:01 | *01:02 |
| **23** | B | m | 54 | n.A. | n.A. | 56 | *02:01 | *02:01 | *07:02 | *51:01 | *07:02 | *14:02 | *04:01 | *04:01 | *03:01 | *06:03 | *04:07 | *13:01 | *01:03 | *03:01 |
| **24** | B | f | 39 | n.A. | n.A. | 56 | *02:01 | *02:01 | *37:01 | *44:03 | *06:02 | *06:02 | *03:01 | *10:01 | *03:01 | *05:01 | *01:01 | *04:07 | *01:01 | *03:01 |
| **25** | B | m | 24 | n.A. | n.A. | 56 | *02:01 | *11:01 | *27:05 | *35:01 | *02:02 | *04:01 | *04:01 | *04:01 | *02:01 | *03:02 | *04:04 | *07:01 | *02:01 | *03:01 |
| **26** | B | m | 46 | n.A. | n.A. | 28 | *02:01 | *24:02 | *39:06 | *40:01 | *03:04 | *07:02 | *03:01 | *04:02 | *03:01 | *06:04 | *11:01 | *13:02 | NI | NI |
| **27** | B | f | 49 | n.A. | n.A. | 56 | *01:01 | *03:01 | *07:02 | *08:01 | *07:01 | *07:02 | *01:01 | *04:01 | *02:01 | *06:02 | *03:01 | *15:01 | *01:02 | *05:01 |
| **28** | B | m | 63 | n.A. | n.A. | 56 | *02:01 | *02:01 | *07:02 | *57:01 | *06:02 | *07:02 | *04:01 | *04:02 | *03:01 | *06:02 | *11:01 | *15:01 | *01:02 | *05:01 |
| **29** | B | f | 53 | n.A. | n.A. | 56 | *02:01 | *03:01 | *15:01 | *44:27 | *04:01 | *07:04 | *01:01 | *04:01 | *03:01 | *05:02 | *12:01 | *16:01 | *01:02 | *05:01 |
| **30** | B | f | 55 | n.A. | n.A. | 56 | *01:01 | *30:04 | *08:01 | *50:01 | *06:02 | *07:01 | *04:01 | *09:01 | *02:01 | *02:01 | *03:01 | *07:01 | *02:01 | *05:01 |
| **31** | B | m | 26 | n.A. | n.A. | 56 | *03:01 | *68:01 | *07:02 | *15:01 | *03:04 | *07:02 | *02:01 | *04:01 | *03:02 | *06:02 | *04:01 | *15:01 | *01:02 | *03:01 |
| **32** | B | f | 54 | n.A. | n.A. | 28 | *03:01 | *03:01 | *51:01 | *51:01 | *01:02 | *02:02 | *03:01 | *04:01 | *03:01 | *05:01 | *01:01 | *11:01 | *01:01 | *05:01 |
| **33** | B | m | 39 | n.A. | n.A. | 21 | *11:01 | *29:02 | *44:03 | *56:01 | *01:02 | *16:01 | *06:01 | *14:01 | *05:01 | *05:01 | *01:01 | *01:01 | *01:01 | *01:01 |
| **34** | B | f | 30 | BNT162b2 | n.A. | 499 | *01:01 | *02:01 | *07:02 | *40:01 | *03:04 | *07:02 | NI | NI | *06:03 | *06:04 | *13:01 | *13:02 | NI | NI |
| **35** | C | f | 56 | AZD1222 | n.A. | 90 | *11:01 | *24:02 | *35:01 | *40:02 | *02:02 | *04:01 | *02:01 | *04:01 | *02:02 | *03:01 | *07:01 | *11:01 | *02:01 | *05:01 |
| **36** | C | m | 51 | NI | n.A. | 56 | *01:01 | *02:01 | *15:01 | *35:02 | *03:04 | *04:01 | *02:01 | *04:01 | *03:01 | *03:02 | *04:01 | *11:04 | NI | NI |
| **37** | C | f | 64 | NI | n.A. | 63 | *02:01 | *30:01 | *13:02 | *15:01 | *03:03 | *06:02 | *02:01 | *17:01 | *02:02 | *03:01 | *04:08 | *07:01 | *02:01 | *03:03 |
| **38** | C | m | 68 | NI | n.A. | 63 | *01:01 | *02:01 | *08:01 | *15:01 | *03:04 | *07:01 | *02:02 | *04:01 | *02:01 | *03:02 | *03:01 | *04:01 | *03:01 | *05:01 |
| **39** | C | f | 46 | BNT162b2 | n.A. | 4 | *29:02 | *31:01 | *44:03 | *50:01 | *06:02 | *16:01 | *04:01 | *11:01 | *02:01 | *02:01 | *07:01 | *07:01 | *02:01 | *02:01 |
| **40** | C | m | 48 | BNT162b2 | n.A. | 4 | *11:01 | *05:01 | *40:01 | *56:01 | *01:02 | *03:04 | *04:01 | *04:01 | *03:01 | *06:03 | *04:01 | *13:01 | *01:03 | *03:01 |
| **41** | C | f | 50 | AZD1222 | n.A. | 56 | *01:01 | *23:01 | *35:01 | *40:01 | *03:04 | *04:01 | *04:01 | *04:01 | *05:01 | *06:02 | *01:01 | *15:01 | *01:01 | *01:02 |
| **42** | C | m | 50 | AD26.COV2.S | n.A. | 34 | *02:01 | *24:02 | *44:02 | *51:01 | *02:02 | *05:01 | *04:01 | *04:01 | *03:01 | *03:01 | *04:01 | *04:01 | *03:01 | *03:01 |
| **43** | C | m | 34 | mRNA-1273 | n.A. | 30 | *01:01 | *68:02 | *08:01 | *14:02 | *07:01 | *08:02 | *01:01 | *02:01 | *02:01 | *03:01 | *03:01 | *13:03 | *05:01 | *05:01 |
| **44** | C | f | 27 | mRNA-1273 | n.A. | 30 | *11:01 | *24:02 | *15:01 | *40:02 | *02:02 | *03:03 | *02:01 | *04:01 | *03:01 | *03:01 | *11:01 | *11:03 | *05:01 | *05:01 |
| **45** | C | f | 23 | mRNA-1273 | n.A. | 49 | *01:01 | *32:01 | *08:01 | *15:01 | *04:01 | *07:01 | *01:01 | *03:01 | NI | NI | *02:01 | *06:03 | *03:01 | *13:01 |
| **46** | C | f | 55 | BNT162b2 | n.A. | 8 | *01:01 | *03:01 | *07:02 | *40:01 | *03:04 | *07:02 | *03:01 | *04:01 | *06 | *06 | *13:02 | *15:01 | NI | NI |
| **47** | A | f | 42 | BNT162b2 | n.A. | 18 | *02:01 | *24:02 | *40:01 | *51:01 | *03:04 | *05:01 | *15:01 | *15:01 | *03:01 | *06:03 | *11:03 | *13:01 | NI | NI |
| **48** | A | f | 37 | NI | n.A. | 12 | *02:01 | *31:01 | *38:01 | *51:01 | *04:01 | *12:03 | NI | NI | NI | NI | NI | NI | NI | NI |
| **49** | A | f | 49 | BNT162b2 | n.A. | 18 | *03:01 | *68:01 | *07:02 | *35:03 | *04:01 | *07:02 | *03:01 | *04:01 | *03:01 | *03.01 | *04:01 | *11:01 | NI | NI |
| **50** | A | f | 41 | BNT162b2 | n.A. | 26 | *02:01 | *02:01 | *15:01 | *39:01 | *03:04 | *07:02 | *02:01 | *03:01 | *04:02 | *05:01 | *01:01 | *08:01 | NI | NI |
| **51** | A | m | 26 | BNT162b2 | n.A. | 5 | *03:01 | *03:01 | *08:01 | *15:01 | *06:02 | *07:01 | NI | NI | *02:01 | *03:02 | *03:01 | *04:01 | NI | NI |
| **52** | A | m | 44 | NI | n.A. | 28 | *03:01 | *29:02 | *07:02 | *44:03 | *07:02 | *16:01 | *02:01 | *11:01 | *02:02 | *06:02 | *07:01 | *15:01 | NI | NI |
| **53** | A | f | 54 | BNT162b2 | n.A. | 19 | *01:01 | *03:01 | *08:01 | *35:01 | *04:01 | *07:01 | *04:02 | *17:01 | *05:01 | *06:02 | *01:01 | *15:01 | NI | NI |
| **54** | A | f | 26 | BNT162b2 | n.A. | 46 | *01:01 | *25:01 | *08:01 | *18:01 | *07:01 | *12:03 | *04:01 | *04:01 | *02:01 | *05:01 | *01:01 | *15:01 | NI | NI |
| **55** | A | f | 37 | BNT162b2 | n.A. | 20 | *02:01 | *25:01 | *44:02 | *56:01 | *01:02 | *05:01 | NI | NI | *05:01 | *05:01 | *01:01 | *01:01 | NI | NI |
| **56** | A | f | 30 | BNT162b2 | n.A. | 55 | *03:01 | *11:01 | *07:02 | *51:01 | *07:02 | *14:02 | *01:01 | *04:01 | *03:01 | *03:01 | *11:01 | *13:05 | NI | NI |
| **57** | A | f | 34 | BNT162b2 | n.A. | 55 | *02:01 | *02:01 | *18:01 | *44:20 | *05:01 | *12:03 | *23:01 | *23:01 | *03:02 | *06:02 | *04:01 | *15:01 | NI | NI |
| **58** | A | m | 30 | BNT162b2 | n.A. | 56 | *01:01 | *01:01 | *08:01 | *08:01 | *07:01 | *07:01 | *02:01 | *03:01 | *02:01 | *03:01 | *03:01 | *11:01 | NI | NI |
| **59** | A | f | 34 | BNT162b2 | n.A. | 50 | *01:01 | *02:01 | *08:01 | *15:01 | *03:03 | *07:01 | *04:01 | *04:01 | *03:01 | *06:03 | *04:01 | *13:01 | NI | NI |
| **60** | A | f | 32 | AZD1222 | n.A. | 83 | *02:01 | *03:01 | *27:05 | *44:02 | *02:02 | *05:01 | *04:01 | *04:01 | *05:03 | *06:02 | NI | *15:01 | NI | NI |
| **61** | A | f | 31 | BNT162b2 | n.A. | 57 | *02:01 | *68:01 | *15:01 | *51:01 | *03:04 | *14:02 | NI | NI | *06:01 | *06:03 | *13:01 | *13:01 | NI | NI |
| **62** | A | f | 55 | mRNA-1273 | n.A. | 63 | *02:01 | *24:02 | *35:03 | *40:01 | *03:04 | *04:01 | NI | NI | *03:01 | *06:04 | *11:01 | *13:02 | NI | NI |
| **63** | A | m | 50 | BNT162b2 | n.A. | 91 | *02:01 | *23:01 | *18:01 | *39:06 | *07:01 | *07:02 | *02:01 | *04:01 | *03:01 | *03:01 | *11:01 | *11:04 | NI | NI |
| **64** | A | m | 40 | NI | n.A. | 54 | *24:02 | *29:02 | *15:09 | *40:01 | *03:04 | *07:04 | *14:01 | *14:01 | *06:04 | *06:04 | *13:02 | *13:02 | NI | NI |
| **65** | A | f | 28 | BNT162b2 | n.A. | 55 | *01:01 | *02:01 | *08:01 | *44:02 | *05:01 | *07:01 | *04:01 | *04:01 | *02:01 | *03:01 | *03:01 | *04:01 | NI | NI |
| **66** | A | f | 28 | mRNA Vaccine | n.A. | 50 | *02:01 | *02:01 | *15:01 | *37:01 | *04:01 | *12:03 | *02:01 | *02:01 | *03:01 | *03:03 | *07:01 | *11:01 | NI | NI |
| **67** | A | f | 27 | mRNA Vaccine | n.A. | 86 | *01:01 | *02:01 | *08:01 | *51:01 | *07:01 | *15:02 | *04:01 | *04:01 | *02:01 | *03:02 | *03:01 | *04:04 | NI | NI |
| **68** | A | m | 32 | mRNA-1273 | n.A. | 65 | *02:01 | *26:01 | *38:01 | *51:01 | *12:03 | *12:03 | NI | NI | NI | NI | *16:01 | *16:01 | NI | NI |
| **69** | A | f | 32 | BNT162b2 | n.A. | 128 | *01:01 | *24:02 | *08:01 | *55:01 | *03:03 | *07:01 | *01:01 | *05:01 | *02:01 | *06:04 | *03:01 | *13:02 | NI | NI |
| **70** | A | f | 26 | BNT162b2 | n.A. | 110 | *02:01 | *02:01 | *14:01 | *44:02 | *05:01 | *08:02 | *04:01 | *14:01 | *02:01 | *06:02 | *07:01 | *15:01 | NI | NI |
| **71** | A | f | 28 | BNT162b2 | n.A. | 94 | *03:01 | *32:01 | *07:02 | *40:02 | *02:02 | *07:02 | *11:01 | *11:01 | *02:01 | *06:02 | *07:01 | *15:01 | NI | NI |
| **72** | A | f | 62 | AZD1222 | n.A. | 113 | *03:01 | *74:03 | *35:01 | *44:03 | *04:01 | *04:01 | NI | NI | *05:01 | *06:02 | *01:01 | *15:01 | NI | NI |
| **73** | A | f | 42 | AZD1222/BNT162b2 | n.A. | 82 | *03:01 | *03:01 | *14:01 | *40:01 | *03:04 | *15:05 | *03:01 | *11:01 | *02:02 | *03:02 | *04:01 | *07:01 | NI | NI |
| **74** | D | f | NI | NI | short disease | NI | *02:01 | *02:01 | *27:05 | *50:01 | *02:02 | *06:02 | NI | NI | *02:01 | *03:02 | *03:01 | *04:04 | 05MZ | *03:01 |
| **75** | D | f | NI | NI | short disease | NI | *01:01 | *02:01 | *07:02 | *37:01 | *07:02 | *06:02 | NI | NI | *03:02 | *06:02 | *04:01 | *13:01 | 03*01 | *01:03 |
| **76** | D | m | NI | NI | short disease | NI | *01:01 | *32:01 | *08:01 | *44:01 | *07:01 | *16:04 | NI | NI | *02:01 | *06:03 | *03:01 | *13:01 | 05MZ | *01:03 |
| **77** | D | f | NI | NI | short disease | NI | *02:01 | *11:01 | *15:01 | *51:01 | *03:03 | *15:02 | NI | NI | *03:01 | *06:03 | *11:01 | *13:01 | 05MZ | *01:03 |
| **78** | D | f | NI | NI | short disease | NI | *02:01 | *24:02 | *44:02 | *07:02 | *05:01 | *07:02 | NI | NI | *04:02 | *06:03 | *08:01 | *13:01 | *04:01 | *01:03 |
| **79** | D | f | NI | NI | short disease | NI | *02:01 | *24:02 | *27:05 | *39:06 | *02:02 | *07:02 | NI | NI | *03:03 | *03:01 | *09:01 | *11:01 | 03BC | 05MZ |
| **80** | D | f | NI | NI | short disease | NI | *02:01 | *02:01 | *15:01 | *49:01 | *03:03 | *07:01 | NI | NI | *03:01 | *06:03 | *11:01 | *13:01 | 05MZ | *01:03 |
| **81** | D | f | NI | NI | short disease | NI | *11:01 | *01:01 | *35:01 | *27:05 | *04:01 | *02:02 | NI | NI | *02:01 | *03:01 | *03:01 | *13:05 | NI | NI |
| **82** | D | m | NI | NI | long disease | NI | *01:01 | *26:01 | *08:01 | *45:01 | *07:01 | *06:02 | NI | NI | *04:02 | *03:01 | *08:01 | *13:03 | *04:01 |  |
| **83** | D | m | NI | NI | long disease | NI | *24:02 | *66:01 | *41:02 | *07:04 | *17:01 | *07:02 | NI | NI | *03:01 | *06:02 | *13:03 | *15:01 | *01:02 |  |
| **84** | D | f | NI | NI | long disease | NI | *03:01 | *01:01 | *07:02 | *57:01 | *07:02 | *06:02 | NI | NI | *03:03 | *06:02 | *07:01 | *15:01 | *02:01 | *01:02 |
| **85** | D | f | NI | NI | long disease | NI | *03:01 | *02:17 | *07:02 | *18:01 | *07:02 | *07:01 | NI | NI | *02:02 | *06:02 | *07:01 | *15:01 | *02:01 | *01:02 |
| **86** | D | f | NI | NI | long disease | NI | *03:01 | *02:01 | *07:02 | *35:01 | *07:02 | *04:01 | NI | NI | *05:01 | *06:02 | *01:01 | *15:01 | *01:01 | *01:02 |
| **87** | D | f | NI | NI | long disease | NI | *02:01 | *02:01 | *44:27 | *40:01 | *07:04 | *03:04 | NI | NI | *06:02 | *06:02 | *15:01 | *16:01 | *01:02 | *01:02 |
| **88** | D | f | NI | NI | long disease | NI | *03:01 | *02:01 | *07:02 | *41:02 | *07:02 | *17:01 | NI | NI | *03:01 | *06:02 | *13:03 | *15:01 | *01:02 |  |
| **89** | D | m | NI | NI | long disease | NI | *11:01 | *68:02 | *35:01 | *14:02 | *04:01 | *08:02 | NI | NI | *05:01 | *06:02 | *01:01 | *15:01 | *01:01 | *01:02 |
| **90** | D | f | NI | NI | long disease | NI | *02:01 | *11:01 | *07:02 | *44:27 | *07:02 | *07:04 | NI | NI | *06:02 | *05:02 | *15:01 | *16:01 | *01:02 | *01:02 |
| **91** | D | m | NI | NI | mild disease | NI | *03:01 | *24:02 | *35:01 | *18:01 | *04:01 | *07:01 | NI | NI | *05:01 | *03:01 | *01:01 | *12:01 | *01:01 | 05MZ |
| **92** | D | m | NI | NI | mild disease | NI | *68:01 | *03:01 | *07:05 | *38:01 | *15:05 | *12:03 | NI | NI | *03:05 | *06:03 | *04:03 | *13:01 | *03 | *01:03 |
| **93** | D | m | NI | NI | mild disease | NI | *11:01 | *26:01 | *35:01 | *38:01 | *04:01 | *12:03 | NI | NI | *03:01 | *06:03 | *11:04 | *13:01 | 05MZ | *01:03 |
| **94** | D | f | NI | NI | mild disease | NI | *30:01 | *32:01 | *38:01 | *15:01 | *12:03 | *03:03 | NI | NI | *03:01 | *06:03 | *11:01 | *13:01 | 05MZ | *01:03 |
| **95** | D | f | NI | NI | mild disease | NI | *24:02 | *24:02 | *07:02 | *35:03 | *07:02 | *04:01 | NI | NI | *03:01 | *06:02 | *12:01 | *15:01 | *01:02 |  |
| **96** | D | m | NI | NI | mild disease | NI | *01:01 | *32:01 | *08:01 | *04:02 | *07:01 | *02:02 | NI | NI | *02:01 | *03:01 | *03:01 | *12:01 | 05MZ | 05MZ |
| **97** | D | m | NI | NI | mild disease | NI | *01:01 | *02:01 | *08:01 | *07:02 | *07:01 | *07:02 | NI | NI | *02:01 | *06:02 | *03:01 | *15:01 | *01:02 |  |
| **98** | D | f | NI | NI | mild disease | NI | *02:01 | *24:02 | *07:02 | *49:01 | *07:02 | *07:01 | NI | NI | *03:01 | *06:02 | *11:04 | *15:01 | 05MZ | *01:02 |
| **99** | D | f | NI | NI | mild disease | NI | *24:02 | *66:01 | *38:01 | *27:05 | *12:03 | *02:02 | NI | NI | *06:03 | *06:04 | *13:01 | *13:02 | *01:03 | *01:02 |
| **100** | D | f | NI | NI | mild disease | NI | *26:01 | *32:01 | *38:01 | *38:01 | *12:03 | *12:03 | NI | NI | *03:02 | *06:03 | *04:02 | *13:01 | *03:01 | *01:03 |
| **101** | D | m | NI | NI | mild disease | NI | *02:01 | *11:01 | *07:02 | *18:01 | *07:02 | *07:01 | NI | NI | *06:04 | *03:01 | *13:02 | *11:04 | *01:02 | 05MZ |
| **102** | D | m | NI | NI | mild disease | NI | *02:01 | *24:02 | *07:02 | *14:02 | *07:02 | *08:02 | NI | NI | *05:01 | *02:02 | *02:0 | *07:01 | *01:01 | *01:02 |
| **103** | D | f | NI | NI | mild disease | NI | *02:01 | *02:01 | *07:02 | *51:01 | *07:02 | *14:02 | NI | NI | *03:01 | *03:01 | *11:01 | *11:04 | 05MZ | 05MZ |
| **104** | D | m | NI | NI | mild disease | NI | *02:01 | *30:02 | *44:02 | *18:01 | *05:01 | *05:01 | NI | NI | *02:01 | *03:01 | *03:01 | *12:01 | 05MZ | 05MZ |
| **105** | D | f | NI | NI | moderate disease | NI | *32:01 | *68:01 | *57:01 | *44:02 | *06:02 | *05:01 | NI | NI | *03:03 | *03:01 | *07:01 | *11:01 | *02:01 | 05MZ |
| **106** | D | m | NI | NI | moderate disease | NI | *01:01 | *02:01 | *57:01 | *07:02 | *06:02 | *07:02 | NI | NI | *02:01 | *06:02 | *03:01 | *15:01 | *01:02 |  |
| **107** | D | f | NI | NI | moderate disease | NI | *01:01 | *02:01 | *15:01 | *57:01 | *03:04 | *06:02 | NI | NI | *03:02 | *06:02 | *04:01 | *15:01 | *03:01 | *01:02 |
| **108** | D | m | NI | NI | moderate disease | NI | *24:02 | *29:01 | *35:02 | *57:01 | *04:01 | *06:02 | NI | NI | *03:01 | *06:04 | *11:01 | *13:02 | 05MZ | *01:02 |
| **109** | D | f | NI | NI | moderate disease | NI | *11:01 | *02:01 | *35:01 | *40:01 | *04:01 | *03:02 | NI | NI | *05:01 | *03:03 | *01:01 | *07:01 | *01:01 | *02:01 |
| **110** | D | m | NI | NI | moderate disease | NI | *02:01 | *03:01 | *57:01 | *35:01 | *06:02 | *04:01 | NI | NI | *05:01 | *03:01 | *01:01 | *04:08 | *01:01 | 03BC |
| **111** | D | f | NI | NI | moderate disease | NI | NI | NI | NI | NI | NI | NI | NI | NI | *03:03 | *03:01 | *07:01 | *11:01 | *02:01 | 05MZ |
| **112** | D | m | NI | NI | moderate disease | NI | *30:01 | *32:01 | *08:01 | *57:01 | *07:01 | *06:02 | NI | NI | *02:01 | *04:02 | *03:01 | *08:01 | 05MZ | *04:01 |
| **113** | D | f | NI | NI | moderate disease | NI | *02:05 | *24:02 | *50:01 | *40:01 | *06:02 | *03:04 | NI | NI | *02:02 | *03:03 | *07:01 | *07:01 | *02:01 | *02:01 |

* vaccinated individuals are considered as fully vaccinated having received at least two vaccine doses

**Supplementary table 2. *In silico* tested HLA Class I and Class II alleles using NetMHCpan for the evaluation of the total amount of potential SARS-CoV-2 binding peptides.** HLA Class I (1^st^ – 3^rd^ column) and HLA Class II alleles (4^th^ column) included in the *in silico* prediction of potential binding peptides derived from the SARS-CoV-2 Spike, Nucleocapsid and Membrane proteins.

| **HLA Class I alleles** | | | **HLA Class II alleles** |
| --- | --- | --- | --- |
| **HLA-A alleles** | **HLA-B alleles** | **HLA-C alleles** | **HLA-DRB1 alleles** |
| HLA-A*0101 | HLA-B*0702 | HLA-C*0102 | HLA-DRB1*0101 |
| HLA-A*0201 | HLA-B*0801 | HLA-C*0202 | HLA-DRB1*0301 |
| HLA-A*0301 | HLA-B*1402 | HLA-C*0303 | HLA-DRB1*0401 |
| HLA-A*1101 | HLA-B*1501 | HLA-C*0304 | HLA-DRB1*0701 |
| HLA-A*2402 | HLA-B*1502 | HLA-C*0401 | HLA-DRB1*0901 |
| HLA-A*2601 | HLA-B*1801 | HLA-C*0501 | HLA-DRB1*1501 |
| HLA-A*2902 | HLA-B*2705 | HLA-C*0602 | HLA-DRB1*1101 |
| HLA-A*3303 | HLA-B*3501 | HLA-C*0701 | HLA-DRB1*1301 |
| HLA-A*6801 | HLA-B*4001 | HLA-C*0702 | HLA-DRB1*1401 |
|  | HLA-B*4002 | HLA-C*0801 | HLA-DRB1*0404 |
|  | HLA-B*4402 |  | HLA-DRB1*0405 |
|  | HLA-B*4403 |  | HLA-DRB1*0407 |
|  | HLA-B*4501 |  | HLA-DRB1*0411 |
|  |  |  | HLA-DRB1*0803 |
|  |  |  | HLA-DRB1*1302 |

**Supplementary Table 3. Statistical assessment of the most dominant haplotypes for different parameters of mild vs. moderate disease courses divided into male and female.** Distinct parameters have been identified as hazardous (unfavorable) for the disease course upon correlation of the parameters: age, sex, blood group, CCR5 expression with the disease duration.

|  |  |  | **OR** | **2.5 %** | **97.5 %** | p |
| --- | --- | --- | --- | --- | --- | --- |
| **All** | (Intercept) |  | 0.3244 | 0.1896 | 0.5509 | < 0.001*** |
|  | Age_sympt |  | 10159 | 10057 | 10262 | 0.0022 ** |
|  | male1 |  | 12086 | 0.9397 | 15542 | 0.1398 |
|  | BG0_allele |  | 0.9375 | 0.7995 | 10995 | 0.4267 |
|  | CCR5_del |  | 0.9487 | 0.6879 | 13026 | 0.7463 |
|  | HLA_ABC_04 | A*02:01_B*07:02_C*07:02 | 0.2411 | 0.1221 | 0.4373 | < 0.001*** |
|  | HLA_ABC_05 |  | 0.8534 | 0.4688 | 15171 | 0.5947 |
|  | HLA_ABC_07 | A*24:02_B*07:02_C*07:02 | 23871 | 11931 | 48621 | 0.0145 * |
|  | HLA_DRB_DQA_DQB_04 |  | 0.9385 | 0.6833 | 12831 | 0.6926 |
|  | HLA_DRB_DQA_DQB_05 | DRB1*11:01_DQA1*05:01_DQB1*03:01 | 11028 | 0.7806 | 15511 | 0.5760 |
|  | HLA_DRB_DQA_DQB_06 | DRB1*07:01_DQA1*02:01_DQB1*03:01 | 22051 | 13671 | 35853 | 0.0013 ** |
|  | HLA_DRB_DQA_DQB_15 | DRB1*12:01_DQA1*05:01_DQB1*03:01 | 0.2475 | 0.0912 | 0.5670 | 0.0023 ** |
| **Male** | (Intercept) |  | 0.2741 | 0.1127 | 0.6502 | 0.0037 ** |
|  | Age_sympt |  | 10.262 | 10.100 | 10.431 | 0.0016 ** |
|  | BG0_allele |  | 0.7845 | 0.6029 | 10.190 | 0.0694 . |
|  | CCR5_del |  | 10.038 | 0.6155 | 16.255 | 0.9876 |
|  | HLA_ABC_04 | A*02:01_B*07:02_C*07:02 | 0.3323 | 0.1054 | 0.8730 | 0.0372 * |
|  | HLA_ABC_05 |  | 0.2303 | 0.0352 | 0.8734 | 0.0589 . |
|  | HLA_ABC_07 | A*24:02_B*07:02_C*07:02 | 118.633 | 27.603 | 859.343 | 0.0033 ** |
|  | HLA_DRB_DQA_DQB_04 |  | 10.582 | 0.6550 | 17.009 | 0.8158 |
|  | HLA_DRB_DQA_DQB_05 | DRB1*11:01_DQA1*05:01_DQB1*03:01 | 19.117 | 10.667 | 34.509 | 0.0299 * |
|  | HLA_DRB_DQA_DQB_06 | DRB1*07:01_DQA1*02:01_DQB1*03:01 | 16.645 | 0.7134 | 38.649 | 0.2325 |
|  | HLA_DRB_DQA_DQB_15 | DRB1*12:01_DQA1*05:01_DQB1*03:01 | 0.2950 | 0.0919 | 0.7794 | 0.0224 * |

|  |  |  | **OR** | **2.5 %** | **97.5 %** | p |
| --- | --- | --- | --- | --- | --- | --- |
| **Female** | (Intercept) |  | 0.4276 | 0.2118 | 0.8544 | 0.0168 * |
|  | Age_sympt |  | 10.092 | 0.9955 | 10.231 | 0.1899 |
|  | BG0_allele |  | 10.142 | 0.8248 | 12.484 | 0.8938 |
|  | CCR5_del |  | 0.8472 | 0.5386 | 13.168 | 0.4660 |
|  | HLA_ABC_04 | A*02:01_B*07:02_C*07:02 | 0.1976 | 0.0800 | 0.4196 | < .001*** |
|  | HLA_ABC_05 |  | 12.615 | 0.6370 | 24.803 | 0.5000 |
|  | HLA_ABC_07 | A*24:02_B*07:02_C*07:02 | 12.533 | 0.5114 | 29.912 | 0.6123 |
|  | HLA_DRB_DQA_DQB_04 |  | 0.8418 | 0.5425 | 12.898 | 0.4346 |
|  | HLA_DRB_DQA_DQB_05 | DRB1*11:01_DQA1*05:01_DQB1*03:01 | 0.8613 | 0.5508 | 13.307 | 0.5059 |
|  | HLA_DRB_DQA_DQB_06 | DRB1*07:01_DQA1*02:01_DQB1*03:01 | 28.228 | 15.525 | 52.540 | < .001*** |
|  | HLA_DRB_DQA_DQB_15 | DRB1*12:01_DQA1*05:01_DQB1*03:01 | 0.0000 | NA | 7.634.917.220.000 | 0.9755 |

*Signif*. codes: 0 ' *** ' 0.001 ' ** ' 0.01 ' * ' 0.05 '.' 0.1 ' ' 1

**Supplementary Table 4. Correlation analysis of the most dominant haplotypes for different parameters of short vs. long disease courses.** Distinct parameters have been identified as hazardous (unfavorable) for the disease course upon correlation of the parameters: age, sex, blood group, CCR5 expression with the disease duration.

|  | **HLA halotype** | **estimate** | **std.error** | **statistic** | **p.value** | **conf.low** | **conf.high** |
| --- | --- | --- | --- | --- | --- | --- | --- |
| **All** | Age_sympt | 0.9870 | 0.0025 | -52204 | < .001*** | 0.9822 | 0.9919 |
|  | male1 | 11737 | 0.0653 | 24511 | 0.0142 * | 10326 | 13341 |
|  | BG0_allele | 0.9824 | 0.0407 | -0.4372 | 0.6620 | 0.9071 | 10639 |
|  | CCR5_del | 0.9234 | 0.0800 | -0.9958 | 0.3194 | 0.7894 | 10802 |
|  | A*03:01_B*35:01_C*04:01 | 16083 | 0.2255 | 21068 | 0.0351 * | 10337 | 25023 |
|  | A*01:01_B*07:02_C*07:02 | 18291 | 0.2236 | 26999 | 0.0069 ** | 11800 | 28354 |
|  | A*29:02_B*44:03_C*16:01 | 13498 | 0.2747 | 10919 | 0.2749 | 0.7878 | 23125 |
|  | DRB1*15:01_DQA1*01:02_DQB1*06:02 | 0.8887 | 0.0738 | -15990 | 0.1098 | 0.7690 | 10270 |
|  | DRB1*09:01_DQA1*03:02_DQB1*03:03 | 0.9003 | 0.1898 | -0.5534 | 0.5800 | 0.6206 | 13060 |
|  | DRB1*11:01_DQA1*05:01_DQB1*03:01 | 10586 | 0.0873 | 0.6520 | 0.5144 | 0.8921 | 12562 |
|  | DRB1*04:01_DQA1*03:01_DQB1*03:02 | 0.9674 | 0.1206 | -0.2752 | 0.7832 | 0.7638 | 12252 |
|  | DRB1*13:02_DQA1*01:02_DQB1*06:04 | 10217 | 0.1255 | 0.1713 | 0.8640 | 0.7990 | 13066 |
|  | DRB1*16:01_DQA1*01:02_DQB1*05:02 | 0.6987 | 0.1953 | -18361 | 0.0663 . | 0.4765 | 10245 |

|  | | **HLA haptotypes** | | **estimate** | | **std.error** | | **statistic** | **p.value** | **conf.low** | **conf.high** |  |
| --- | --- | --- | --- | --- | --- | --- | --- | --- | --- | --- | --- | --- |
| **Male** | | Age_sympt | | 0.9803 | | 0.0041 | | -48290 | < .001*** | 0.9725 | 0.9883 |  |
|  |  | BG0_allele | | 0.9019 | | 0.0653 | | -15820 | 0.1137 | 0.7936 | 10250 |  |
|  |  | CCR5_del | | 0.6481 | | 0.1264 | | -34317 | < .001*** | 0.5060 | 0.8303 |  |
|  |  | A*03:01_B*35:01_C*04:01 | | 12580 | | 0.2800 | | 0.8198 | 0.4123 | 0.7267 | 21778 |  |
|  |  | A*01:01_B*07:02_C*07:02 | | 11612 | | 0.3138 | | 0.4763 | 0.6339 | 0.6278 | 21477 |  |
|  |  | A*29:02_B*44:03_C*16:01 | | 10328 | | 0.3147 | | 0.1024 | 0.9184 | 0.5574 | 19137 |  |
|  |  | DRB1*15:01_DQA1*01:02_DQB1*06:02 | | 10499 | | 0.1221 | | 0.3990 | 0.6899 | 0.8265 | 13338 |  |
|  |  | DRB1*09:01_DQA1*03:02_DQB1*03:03 | | 20961 | | 0.2916 | | 25382 | 0.0111 * | 11836 | 37119 |  |
|  |  | DRB1*11:01_DQA1*05:01_DQB1*03:01 | | 0.7436 | | 0.1461 | | -20279 | 0.0426 * | 0.5584 | 0.9901 |  |
|  |  | DRB1*04:01_DQA1*03:01_DQB1*03:02 | | 22617 | | 0.2243 | | 36383 | < .001*** | 14571 | 35105 |  |
|  |  | DRB1*13:02_DQA1*01:02_DQB1*06:04 | | 14862 | | 0.2038 | | 19444 | 0.0518 . | 0.9968 | 22158 |  |
|  |  | DRB1*16:01_DQA1*01:02_DQB1*05:02 | | 12658 | | 0.2627 | | 0.8970 | 0.3697 | 0.7563 | 21184 |  |
| **Female** | | Age_sympt | | 0.9898 | | 0.0034 | | -30569 | | 0.0022 ** | 0.9833 | 0.9963 |
|  |  | BG0_allele | | 10367 | | 0.0543 | | 0.6640 | | 0.5067 | 0.9321 | 11531 |
|  |  | CCR5_del | | 12196 | | 0.1104 | | 17990 | | 0.0720 . | 0.9824 | 15141 |
|  |  | A*03:01_B*35:01_C*04:01 | | 53324 | | 0.4591 | | 36459 | | < .001*** | 21684 | 131132 |
|  |  | A*01:01_B*07:02_C*07:02 | | 35314 | | 0.3450 | | 36573 | | < .001*** | 17960 | 69436 |
|  |  | A*29:02_B*44:03_C*16:01 | | 42794 | | 0.5950 | | 24434 | | 0.0145 * | 13333 | 137357 |
|  |  | DRB1*15:01_DQA1*01:02_DQB1*06:02 | | 0.7867 | | 0.0949 | | -25272 | | 0.0115 * | 0.6531 | 0.9476 |
|  |  | DRB1*09:01_DQA1*03:02_DQB1*03:03 | | 0.6110 | | 0.2508 | | -19644 | | 0.0495 * | 0.3738 | 0.9989 |
|  |  | DRB1*11:01_DQA1*05:01_DQB1*03:01 | | 14206 | | 0.1104 | | 31811 | | 0.0015 ** | 11443 | 17636 |
|  |  | DRB1*04:01_DQA1*03:01_DQB1*03:02 | | 0.8559 | | 0.1460 | | -10658 | | 0.2865 | 0.6428 | 11395 |
|  |  | DRB1*13:02_DQA1*01:02_DQB1*06:04 | | 0.8339 | | 0.1640 | | -11078 | | 0.2680 | 0.6046 | 11500 |
|  |  | DRB1*16:01_DQA1*01:02_DQB1*05:02 | | 0.3713 | | 0.3280 | | -30207 | | 0.0025 ** | 0.1952 | 0.7062 |

*Signif*. codes: 0 ' *** ' 0.001 ' ** ' 0.01 ' * ' 0.05 '.' 0.1 ' ' 1

**Supplementary Table 5.** **Unfavorable and favorable HLA Class I and Class II haplotypes**

| **Unfavorable HLA-Class I** | **Haplotypes** |
| --- | --- |
| HLA_ABC_04 | **#02:01_07:02_07:02** |
| HLA_ABC_05 | **#02:01_40:01_03:04** |
| HLA_ABC_11 | **#03:01_35:01_04:01** |
| HLA_ABC_17 | **#01:01_07:02_07:02** |
| HLA_ABC_22 | **#29:02_44:03_16:01** |
| **Unfavorable HLA-Class II** | **Haplotypes** |
| HLA_DRB_DQA_DQB_02 | **#15:01_01:02_06:02** |
| HLA_DRB_DQA_DQB_05 | **#11:01_05:01_03:01** |
| HLA_DRB_DQA_DQB_06 | **#07:01_02:01_03:03** |
| HLA_DRB_DQA_DQB_07 | **#13:01_01:03_06:03** |
| HLA_DRB_DQA_DQB_10 | **#04:01_03:01_03:01** |
| HLA_DRB_DQA_DQB_12 | **#13:02_01:02_06:04** |
| HLA_DRB_DQA_DQB_15 | **#12:01_05:01_03:01** |
| HLA_DRB_DQA_DQB_16 | **#13:03_05:01_03:01** |
| HLA_DRB_DQA_DQB_17 | **#16:01_01:02_05:02** |
| HLA_DRB_DQA_DQB_18 | **#09:01_03:01_03:03** |
|  |  |
| **Favorable HLA-Class I** | **Haplotypes** |
| HLA_ABC_01 | **#01:01_08:01_07:01** |
| HLA_ABC_02 | **#03:01_07:02_07:02** |
| HLA_ABC_03 | **#02:01_44:02_05:01** |
| HLA_ABC_06 | **#02:01_13:02_06:02** |
| HLA_ABC_07 | **#24:02_07:02_07:02** |
| HLA_ABC_08 | **#02:01_35:01_04:01** |
